# Hypothalamic-Pituitary Axis Regulates Hydrogen Sulfide Production

**DOI:** 10.1016/j.cmet.2017.05.003

**Published:** 2017-06-06

**Authors:** Christopher Hine, Hyo-Jeong Kim, Yan Zhu, Eylul Harputlugil, Alban Longchamp, Marina Souza Matos, Preeti Ramadoss, Kevin Bauerle, Lear Brace, John M. Asara, C. Keith Ozaki, Sheue-yann Cheng, Subhankar Singha, Kyo Han Ahn, Alec Kimmelman, Ffolliott M. Fisher, Pavlos Pissios, Dominic J. Withers, Colin Selman, Rui Wang, Kelvin Yen, Valter D. Longo, Pinchas Cohen, Andrzej Bartke, John J. Kopchick, Richard Miller, Anthony N. Hollenberg, James R. Mitchell

**Affiliations:** 1 Department of Genetics and Complex Diseases, Harvard T.H. Chan School of Public Health, Boston, MA 02115, USA; 2 Division of Endocrinology, Diabetes and Metabolism, Beth Israel Deaconess Medical Center, Harvard Medical School, Boston, MA 02215, USA; 3 Division of Signal Transduction, Department of Medicine, Beth Israel Deaconess Medical Center, Harvard Medical School, Boston, MA 02215, USA; 4 Department of Surgery, Heart and Vascular Center Brigham and Women's Hospital, Harvard Medical School, Boston, MA 02115, USA; 5 Laboratory of Molecular Biology, Center for Cancer Research, National Cancer Institute, National Institutes of Health, Bethesda, MD 20892, USA; 6 Department of Chemistry, Center for Electro-Photo Behaviors in Advanced Molecular Systems, POSTECH, 77 Cheongam-Ro, Nam-Gu, Pohang 790-784, Republic of Korea; 7 Department of Radiation Oncology, Dana-Farber Cancer Institute, Boston, MA 02215, USA; 8 Medical Research Council Clinical Science Centre, Imperial College, London W12 0NN, UK; 9 Glasgow Ageing Research Network, Institute of Biodiversity, Animal Health and Comparative Medicine, College of Medical, Veterinary and Life Sciences, University of Glasgow, Glasgow G12 8QQ, UK; 10 Department of Biology, Lakehead University, Thunder Bay, ON P7B 5E1, Canada; 11 Department of Biological Sciences, Longevity Institute, School of Gerontology, University of Southern California, Los Angeles, CA 90089, USA; 12 Department of Internal Medicine, Southern Illinois University School of Medicine, Springfield, IL 62794, USA; 13 Edison Biotechnology Institute, Heritage College of Osteopathic Medicine, Ohio University, Athens, OH 45701, USA; 14 Department of Pathology & Geriatrics Center, University of Michigan, Ann Arbor, MI 48109, USA

**Keywords:** hydrogen sulfide, growth hormone, thyroid hormone, IGF-1, FGF21, IRS-1, longevity, hypopituitary dwarfism, autophagy, cystathionine γ-lyase

## Abstract

Decreased growth hormone (GH) and thyroid hormone (TH) signaling are associated with longevity and metabolic fitness. The mechanisms underlying these benefits are poorly understood, but may overlap with those of dietary restriction (DR), which imparts similar benefits. Recently we discovered that hydrogen sulfide (H_2_S) is increased upon DR and plays an essential role in mediating DR benefits across evolutionary boundaries. Here we found increased hepatic H_2_S production in long-lived mouse strains of reduced GH and/or TH action, and in a cell-autonomous manner upon serum withdrawal in vitro. Negative regulation of hepatic H_2_S production by GH and TH was additive and occurred via distinct mechanisms, namely direct transcriptional repression of the H_2_S-producing enzyme cystathionine γ-lyase (CGL) by TH, and substrate-level control of H_2_S production by GH. Mice lacking CGL failed to downregulate systemic T_4_ metabolism and circulating IGF-1, revealing an essential role for H_2_S in the regulation of key longevity-associated hormones.

## Highlights

• Hepatic H_2_S production capacity is elevated in long-lived hypopituitary mouse models
• Growth hormone (GH) represses hepatic H_2_S production post-transcriptionally
• Thyroid hormone (TH) acts via TRβ to repress cystathionine γ-lyase and H_2_S levels
• H_2_S negatively regulates circulating TH and IGF-1 levels

## Graphical Abstract

**Figure:**
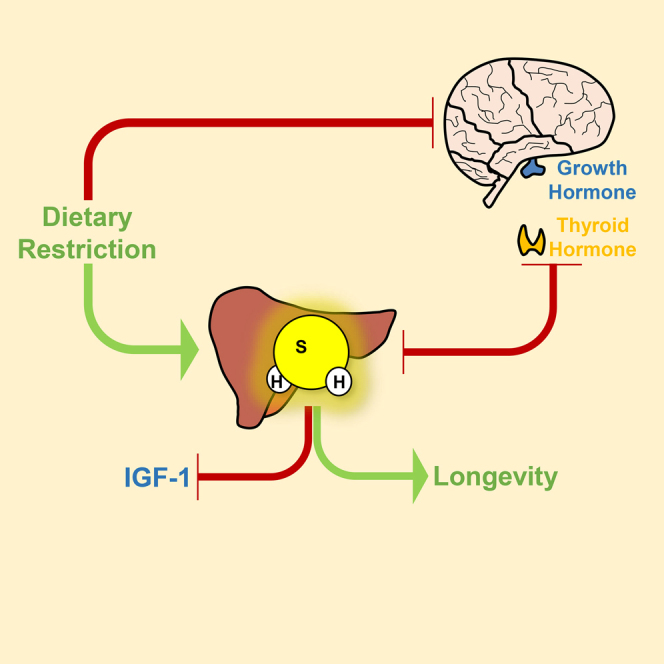

## Teaser

Reduced thyroid hormone (TH) and growth hormone (GH) activity are hallmarks of genetic models of longevity in mice. Here, Hine et al. find that TH and GH negatively regulate hepatic production of the longevity-associated gas hydrogen sulfide, which feeds back to negatively regulate circulating TH and IGF-1 levels.

## Introduction

Hydrogen sulfide (H_2_S) affects numerous aspects of animal physiology (Wang, 2012), including long-term potentiation in the nervous system (Abe and Kimura, 1996), vasorelaxation (Zhao et al., 2001), oxygen sensing (Olson et al., 2006), angiogenesis in the cardiovascular system (Cai et al., 2007, Papapetropoulos et al., 2009), and insulin secretion from endocrine cells in the pancreas (Yang et al., 2005). While H_2_S at high concentrations is toxic, low levels impart numerous benefits including resistance to hypoxia (Blackstone and Roth, 2007), neuroprotection (Kimura and Kimura, 2004), protection from myocardial ischemia reperfusion injury (Bian et al., 2006, Elrod et al., 2007), modulation of inflammation (Zanardo et al., 2006), and extension of longevity (Miller and Roth, 2007). H_2_S is also produced endogenously by several enzymes, including 3-MST and the transsulfuration pathway (TSP) enzymes cystathionine β-lyase (CBS) and cystathionine γ-lyase (CGL) (Kabil et al., 2011). Mice lacking functional TSP activity are hypertensive (Yang et al., 2008), display reduced angiogenic potential (Szabó and Papapetropoulos, 2011), and are susceptible to aging-related neurodegeneration and osteoporosis (Liu et al., 2014, Paul et al., 2014). Despite the implications for its pleiotropic beneficial effects, little is known about the systemic regulation of endogenous H_2_S production.

Previously, we reported that dietary restriction (DR), best known for increasing lifespan, stress resistance, and metabolic fitness in organisms across evolutionary boundaries, works partially through increasing endogenous H_2_S production (Hine et al., 2015). In rodents, DR-mediated protection from hepatic ischemia reperfusion injury requires H_2_S generation by CGL, which is subject to regulation by sulfur amino acid intake (Hine et al., 2015, Nakano et al., 2015, Sikalidis and Stipanuk, 2010). Downstream mechanisms of H_2_S action in this context, and whether these are specific to DR or shared with other anti-aging interventions, remain unknown.

Like DR, reduced hypothalamic-pituitary axis activity is associated with resistance to age-related diseases, extended longevity, and improved metabolic fitness in rodents and humans. Long-lived rodent strains include hypopituitary Snell and Ames dwarf mice that lack growth hormone (GH) and thyroid-stimulating hormone (TSH) (Bartke and Brown-Borg, 2004). Specific ablation of the GH receptor (GHR) in GHR knockout mice (GHRKO) also increases lifespan, suggesting the specificity of the GH pathway in aging (Coschigano et al., 2003). However, TH levels are also reduced in GHRKO mice, complicating the functional dissection of GH and TH activity in longevity control in vivo (Hauck et al., 2001). Subclinical hypothyroidism is associated with longevity in human centenarian studies (Atzmon et al., 2009, Rozing et al., 2010), and inactivating mutations in GHR are associated with metabolic fitness and reduced cancer incidence in humans (Guevara-Aguirre et al., 2011).

GHRKO mice are recalcitrant to further increase in lifespan or insulin sensitivity upon DR, consistent with at least partially overlapping mechanisms of action (Bonkowski et al., 2006). One such candidate mechanism is the insulin-like growth factor-1 (IGF-1) pathway, which is reduced upon DR as well as in hypopituitary/GHRKO longevity models. Here, we tested the hypothesis that increased H_2_S production is a shared phenotype in genetic models of longevity involving decreased GH/TH signaling with the potential to contribute to metabolic benefits. We found that GH and TH negatively regulate hepatic H_2_S production through distinct mechanisms, with functional consequences on feedback control of hepatic IGF-1 and TH production.

## Results

### Increased Hepatic H_2_S Production in Long-Lived Hypopituitary Dwarf Mice In Vivo

**Figure 1:**
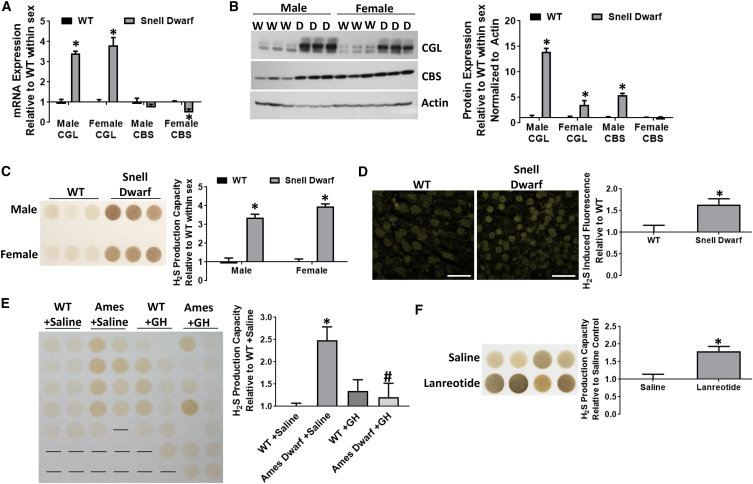
Increased Hepatic H_2_S Production in Long-Lived Hypopituitary Dwarf Mice In Vivo (A–D) Hepatic CGL and CBS mRNA expression (n = 3/group) (A), protein expression (n = 3/group) (B), H_2_S production capacity via the lead sulfide method (n = 3/group) (C), and endogenous H_2_S production via two-photon florescence microscopy (n = 3/group) (D) in male and female WT or Snell dwarf mice as indicated. Scale bar, 25 μm. Asterisk indicates the significance of the difference between genotypes within sex; ^∗^p < 0.05. (E) Liver H_2_S production capacity in 18-month-old female Ames dwarf or WT mice treated +/− growth hormone during postnatal development at weeks 2-8 (n = 9–14/group). The asterisk indicates the significance of the difference between the WT+Saline control group and experimental group, and the # sign indicates the significance of the difference between +Saline and +GH; ^∗^/^#^p < 0.05. (F) Liver H_2_S production capacity in mice treated with saline control or lanreotide (n = 4/group). The asterisks indicate the significance of the difference between treatment groups; ^∗^p < 0.05. Error bars are ± SEM. See also Figure S1.

We examined the impact of reduced GH and TSH signaling on hepatic CGL and CBS mRNA and protein expression and H_2_S production capacity in Snell dwarf mice lacking these hormones (Figures 1A–1D). Male and female Snell dwarf mice had increases in hepatic CGL mRNA (Figure 1A) and protein (upper band) compared with wild-type (WT) littermates (Figure 1B). Hepatic CBS mRNA was not increased in either male or female mice (Figure 1A), but CBS protein levels were increased in male mice compared with WT littermates (Figure 1B). Consistent with enzyme levels, H_2_S production capacity, as measured by the lead sulfide method, was increased in liver homogenates in both male and female Snell dwarf mice (Figure 1C). Importantly, endogenous H_2_S levels, as detected by two-photon microscopy using the H_2_S-specific chemo-fluorescent probe P3 (Singha et al., 2015), were also increased in Snell dwarf livers (Figure 1D).

To test if increased H_2_S production could have functional consequences on hypopituitary dwarf mice, we made use of the fact that GH treatment of Ames dwarf mice during early post-natal development (weeks 2-8) reverses lifespan and metabolic effects (Panici et al., 2010). Early GH treatment normalized (reduced) hepatic H_2_S production capacity (Figure 1E) and CGL protein levels (Figure S1A) measured later in life at 18 months of age. These data demonstrate a correlation between hepatic H_2_S production capacity and longevity in the Ames dwarf model.

Because Snell and Ames dwarf mice lack GH and TH signals from birth, we next tested the plasticity of H_2_S regulation by GH/TH in WT adult mice by pharmacological inhibition with the somatostatin analog, lanreotide (Kuhn et al., 1994). Treatment for 8 days increased liver H_2_S production capacity (Figure 1F) and CGL protein levels (Figure S1B) without affecting body weight (Figure S1C) or food intake (Figure S1D). These data demonstrate that GH and TSH deficiency/inhibition promote hepatic H_2_S production in vivo.

### GH Signaling Inhibits Hepatic H_2_S Production In Vivo

**Figure 2:**
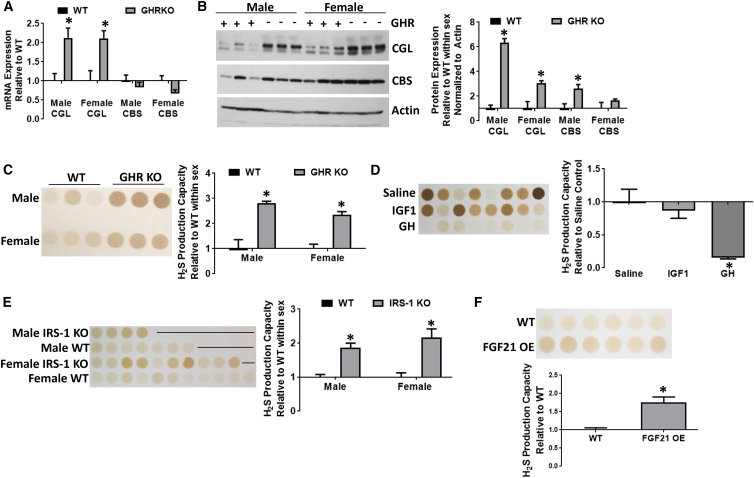
Growth Hormone Signaling Inhibits Hepatic H_2_S Production In vivo (A–C) Hepatic CGL and CBS mRNA expression (n = 3/group) (A), protein expression (n = 3/group) (B), and H_2_S production capacity (n = 3/group) (C) in male and female growth hormone receptor knockout (GHRKO) mice. The asterisk indicates the significance of the difference between genotypes within sex; ^∗^p < 0.05. (D) Liver H_2_S production capacity (n = 8/group) in WT mice treated for 2 weeks with recombinant IGF-1, GH, or saline vehicle as indicated. The asterisk indicates the significance of the difference between GH and saline treatment; ^∗^p < 0.05. (E and F) Liver H_2_S production capacity in male and female IRS-1 WT or KO mice (n = 4–11/group) (E) and in male FGF21 WT or overexpressing (OE) mice (n = 6/group) (F). The asterisk indicates the significance of the difference between genotypes within sex; ^∗^p < 0.05. Error bars are ± SEM. See also Figure S2.

We next focused specifically on the potential of GH to regulate H_2_S production using global GHRKO mice. Male and female GHRKO mice had increases in hepatic CGL mRNA and protein relative to WT littermates (Figures 2A and 2B). As in Snell dwarf mice, CBS protein was increased in GHRKO males, while CBS mRNA expression was unchanged (Figures 2A and 2B). Hepatic H_2_S production capacity was increased in both male and female GHRKO mice compared with WT littermates (Figure 2C). Thus, the lack of GH signaling due to global deletion of its receptor results in increased hepatic H_2_S production capacity in vivo.

As IGF-1 is a major downstream effector of hepatic GH signaling, we tested the effects of 2 weeks of recombinant human IGF-1 or human GH injections on hepatic H_2_S production capacity and CGL protein expression. While IGF-1 had no effect relative to mock treatment, GH injection reduced hepatic H_2_S production capacity (Figure 2D) and hepatic CGL protein expression (Figure S2A). Consistent with GH-mediated repression of H_2_S production capacity, GH injection suppresses CGL mRNA expression according to independent data obtained from NCBI GeoProfile GDS862/8.2.2.10/Cth (Ahluwalia et al., 2004) (Figure S2B). Together, these data indicate that GH and GHR signaling suppress hepatic H_2_S production capacity in vivo independent of IGF-1.

To confirm the specific role of GHR signaling in H_2_S regulation, we tested the potential of two intracellular mediators of hepatic GHR signaling, IRS-1 and FGF21, to alter H_2_S production. IRS-1 is an adaptor protein involved in both insulin and GHR signaling (Liang et al., 1999), and global IRS-1KO mice are long lived (Selman et al., 2011). IRS-1KO mice had increased hepatic H_2_S production capacity (Figure 2E) and elevated CGL protein expression (Figure S2C). Because IRS-1KO mice are insulin resistant (Biddinger et al., 2008), we tested the potential contribution of insulin receptor (IR) signaling using liver-specific insulin receptor knockout (LIRKO mice). However, LIRKO mice displayed decreased hepatic H_2_S production capacity (Figure S2D), consistent with GH signaling rather than IR signaling in negative regulation of hepatic H_2_S production. In addition, long-lived mice overexpressing the fasting hormone FGF21 (Zhang et al., 2012), an intracellular inhibitor of GH signaling (Inagaki et al., 2008), displayed increased hepatic H_2_S production capacity (Figure 2F) and CGL protein (Figure 2E). These data are consistent with GH/GHR as a negative regulator of H_2_S production capacity, and show a positive correlation between increased hepatic H_2_S production capacity and extended longevity in vivo.

### GH/GHR Signaling Inhibits Hepatic H_2_S Production In Vitro

To elucidate how GHR signaling controls H_2_S production, we turned to overnight serum withdrawal with or without added recombinant GH in cell culture as a cell-autonomous model. Endogenous H_2_S production was measured using the fluorescent P3 probe and visualized/quantitated by two-photon microscopy or UV spectrophotometry. We first established the ability of overnight serum withdrawal to induce robust endogenous H_2_S production, and the specific contribution of TSP enzymes CGL and CBS to this process using WT versus CGLKO fibroblasts with or without PAG and AOAA, inhibitors of CGL and CBS, respectively (Figures S3A and S3B). Interestingly, while CGL is the predominant H_2_S producer in liver in vivo (Kabil et al., 2011), and responsible for the increase in H_2_S production capacity in response to DR (Hine et al., 2015), CBS also contributed to the increase in H_2_S production upon serum withdrawal in primary fibroblasts in vitro (Figure S3A).

**Figure 3:**
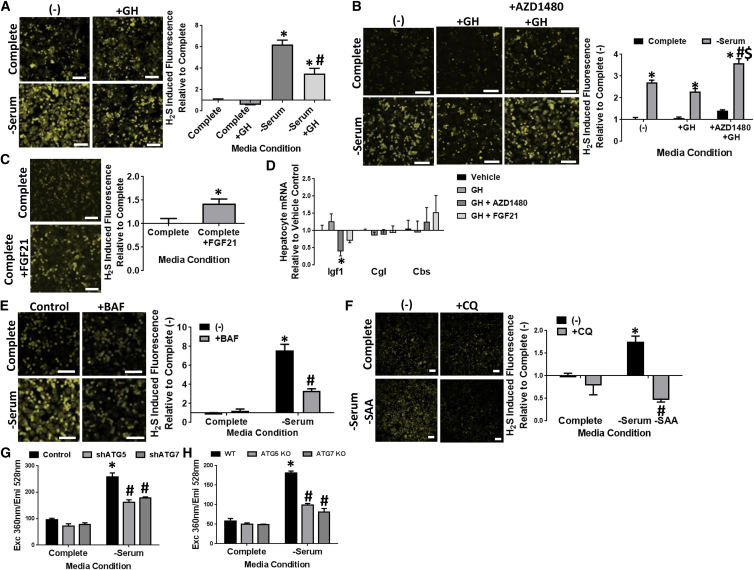
Growth Hormone Receptor Signaling Inhibits Hepatic H_2_S Production In Vitro (A–C) Endogenous H_2_S production in primary mouse hepatocytes as measured via two-photon fluorescent microscopy under different medium conditions: (A) +/−growth serum and +/−GH, with the asterisk indicating the significance of the difference between Complete and −Serum, and the # sign indicating the significance of the difference between −Serum and −Serum+GH, ^∗^^/#^p < 0.05; (B) +/−growth serum, +/−GH, +/−AZD1480, with the asterisk indicating the significance of the difference between Complete and −Serum in each group, and the # sign indicating the significance of the difference between −Serum (−) (no addition) and −Serum+ZD1480; and the $ sign indicating the significance of the difference between +GH and +GH+ZD1480 for both Complete and −Serum conditions, ^∗^^/#/$^p < 0.05; (C) +/−FGF21 in Complete medium containing serum, with the asterisk indicating the significance of the difference between Complete and Complete+FGF21; ^∗^p < 0.05. (D) mRNA expression of *Igf-I*, Cgl, and Cbs in mouse primary hepatocytes. The asterisk indicates the significance of the difference between +GH and +GH+ZD1480; ^∗^p < 0.05. (E and F) Endogenous H_2_S production in mouse primary hepatocytes by florescent microscopy after overnight treatment under the indicated medium conditions followed by addition of the P3 probe for 1 hr. The asterisk indicates the significance of the difference between Complete and −Serum, and the # sign indicates the significance of the difference between −Serum and −Serum+BAF (E), or −Serum−SAA and −Serum−SAA+CQ (F); ^∗^^#^p < 0.05. (G and H) Endogenous H_2_S production in Hepa1-6 cells with or without knockdown of autophagy components ATG5 and ATG7 by shRNA (G) or in MEFs by genetic knockout of ATG5 and ATG7 (H) detected by UV spectrophotometry after overnight treatment with or without serum followed by addition of the P3 probe for 1 hr. The asterisk indicates the significance of the difference between Complete and −Serum in the Control group, and the # sign indicates the significance of the difference between −Serum Control and −Serum ATG5 deficient or −Serum ATG7 deficient; ^∗^^/#^p < 0.05. Each experiment was repeated at least three times. Scale bars, 100 μm (A–C, E, and F). Error bars are ± SEM. See also Figure S3.

In cultured mouse primary hepatocytes, H_2_S production was also significantly increased upon serum deprivation (Figures 3A and S3C). Importantly, addition of recombinant GH at the level required to induce robust phosphorylation of Stat5 (p-Stat5), and transcription of *Igf-1* (Figure S3D–S3F), dampened the increase in H_2_S production induced by serum deprivation (Figures 3A and S3C).

Canonical GH-induced intracellular signaling begins with its binding to and dimerization of the GHR on the plasma membrane, leading to stimulation of numerous signaling cascades, including Jak2/Stat5, followed by the transcriptional/translational regulation of target genes such as *Igf-1*. While addition of GH into medium containing serum did not change H_2_S production, blocking GH signaling with the Jak2 inhibitor AZD1480 (Gu et al., 2013) moderately increased endogenous H_2_S in hepatocytes cultured in Complete medium with additional GH (Figures 3B and S3G). Furthermore, the increase in H_2_S production induced by serum deprivation and blocked by GH was fully restored with addition of AZD1480 (Figures 3B and S3G). Exogenous FGF21 increased H_2_S production despite the presence of full serum (Figures 3C and S3H). Together, these data suggest that GH acts through the Jak2/Stat5 pathway to repress H_2_S production in cells.

Because of increased TSP mRNA/protein expression in Snell/Ames and GHRKO livers in vivo, we next asked if GH regulates hepatic H_2_S production in a cell-autonomous manner via transcriptional control of TSP gene expression, in a manner similar to control of Igf-1 (Figure 3D). Surprisingly, serum withdrawal from primary hepatocytes failed to increase CGL mRNA or protein to the same levels as observed in vivo (Figures S3I and S3J). Similarly, pharmacological inhibition of GH/GHR action with AZD1480 or FGF21 failed to significantly affect TSP gene expression as they did IGF-1 expression (Figure 3D). Together, these data suggest that neither transcriptional nor translational control of CBS or CGL are the major mechanisms of hepatic H_2_S regulation by GH in cells.

Regulation of endogenous H_2_S production via CBS and CGL in cells could instead occur post-translationally or be driven by substrate availability (Kabil et al., 2011, Majtan et al., 2014, Zhao et al., 2014). While the endogenous source of free cysteine for H_2_S production is currently unknown, cellular autophagy is increased in long-lived dwarf mice (Wang and Miller, 2012), thus potentially fueling the increase in H_2_S production observed upon GH signaling inhibition. Consistent with this notion, pharmacological inhibition of autophagy with bafilomycin (BAF) or chloroquine (CQ) blocked H_2_S production induced by serum removal (Figures 3E, 3F, S3K, and S3L), even in the presence of media lacking sulfur amino acids (SAA) (Figure 3F). Similarly, inhibition of autophagy by genetic knockdown (Figures 3G, S3M) or knockout (Figure 3H and S3N) of Autophagy Protein 5 (ATG5) or 7 (ATG7) decreased H_2_S production upon serum withdrawal. Taken together, these data suggest that GH is a negative regulator of H_2_S production in vitro through autophagy-dependent substrate-level and/or enzymatic activity control rather than transcriptional control of CGL and CBS expression.

### Hypothyroidism Increases Hepatic H_2_S Production In Vivo

We next considered the potential of TH to explain the transcriptional control of hepatic CGL expression observed in these models in vivo but not readily attributable to GHR activity in vitro. Adult male mice were made hypo-, eu-, or hyperthyroid by inhibiting endogenous thyroid function with a PTU-containing low iodine diet (PTU/LID) with or without TH supplementation (in the form of T_3_). To validate the system, we confirmed that known TH target genes were regulated as expected in the hypothyroid (PTU/LID), euthyroid (PTU/LID + low dose T3), and hyperthyroid (PTU/LID + high dose T_3_) states (Figure S4A).

**Figure 4:**
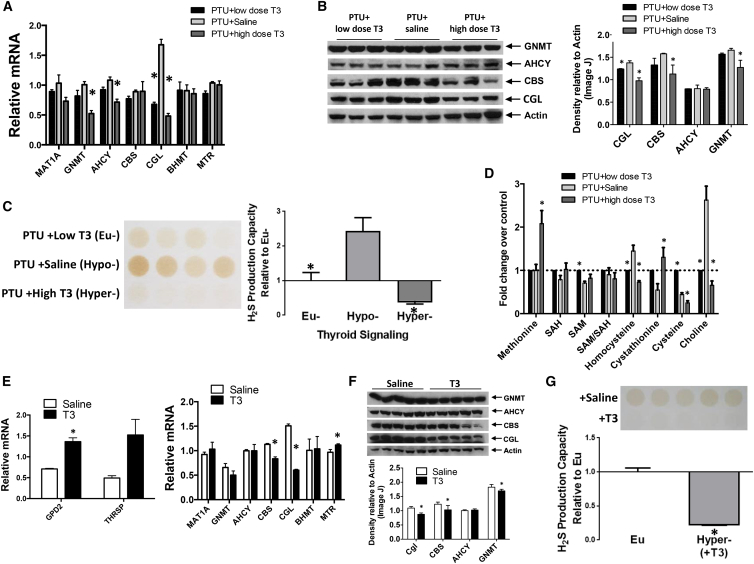
Hypothyroidism Increases and Thyroid Hormone Represses Hepatic H_2_S Production In Vivo (A–D) mRNA expression (n = 4) (A), protein expression (n = 3) (B), H_2_S production capacity (n = 4) (C), and transmethylation/transsulfuration metabolite levels by liquid chromatography-tandem mass spectrometry (n = 4) (D) in livers of mice under hypo- (PTU+Saline), hyper- (PTU+high dose T3), and eu-(PTU+low dose T3) thyroid states. The asterisks indicate the significance of the difference from the hypothyroid state (PTU+Saline); ^∗^p < 0.05. (E–G) Analysis of TH-responsive and sulfur amino acid metabolism-associated mRNA levels (n = 4–6) (E), protein expression (n = 5) (F), and H_2_S production capacity (n = 5) (G) in livers of mice treated with T3 (hyperthyroid) versus vehicle (saline) control (euthyroid). The asterisk indicates the significance of the difference between vehicle and +T3 groups; ^∗^p < 0.05. Error bars are ± SEM. See also Figure S4.

Circulating T_3_ levels strongly correlated with expression of genes in the methionine cycle and TSP including CGL, which was activated in the hypothyroid state and strongly repressed by increasing doses of T_3_ (Figure 4A). A similar correlation was observed between T_3_ and hepatic protein levels of CGL and CBS (Figure 4B) as well as hepatic H_2_S production capacity (Figures 4C and S4B). Metabolomics revealed broader effects of circulating T_3_ on hepatic methionine cycle and TSP metabolites, including regulation of homocysteine, cystathionine, and cysteine (Figure 4D). Finally, we confirmed that T_3_ alone, without the induction of hypothyroidism via a PTU/LID, reduced hepatic CGL and CBS mRNA (Figure 4E), correlating with protein levels (Figure 4F), hepatic H_2_S production capacity (Figure 4G) and characteristic changes in hepatic metabolites, including cystathionine and cysteine (Figure S4C).

### Thyroid Hormone Signaling through TRβ Suppresses Hepatic H_2_S Production

**Figure 5:**
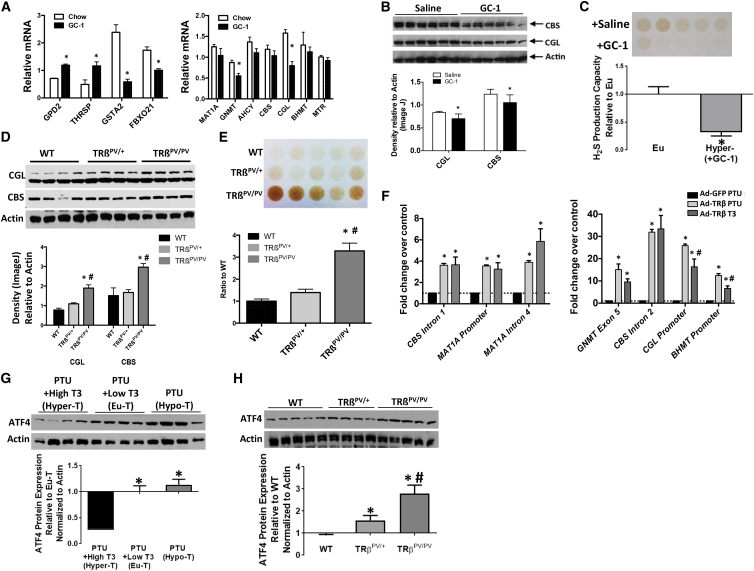
Thyroid Hormone Signaling through TRβ Represses Hepatic H_2_S Production In Vivo (A–C) Analysis of TH-responsive and sulfur amino acid metabolism-associated mRNA levels (n = 4–6) (A), protein expression (n = 6) (B), and H_2_S production capacity (n = 5) (C) in livers of mice treated with GC-1 versus vehicle (saline) control. The asterisk indicates the significance of the difference between vehicle (saline) control (euthyroid) and +GC-1 groups (hyperthyroid); ^∗^p < 0.05. (D and E) Liver CBS and CGL protein expression (D) and H_2_S production capacity (E) in mice with indicated TRβ status (WT, homozygous WT; TRβ^PV/+^, Het; TRβ^PV/PV^, homozygous mutant; n = 4–5/group). The asterisk indicates the significance of the difference between WT and TRβ^PV/PV^, and the # sign indicates the significance of the difference between TRβ^PV/+^ and TRβ^PV/PV^; ^∗^^/#^p < 0.05. (F) Fold enrichment of TRβ binding to genetic regulator elements in sulfur amino acid metabolism and H_2_S producing genes in the livers of mice infected with Ad-GFP (control) or Ad-TRβ while on PTU diets +/−T3 injection as indicated (n = 5/group). The asterisk indicates the significance of the difference between the Ad-GFP PTU and Ad-TRβ PTU or Ad-TRβ T3 groups, and the # sign indicates the significance of the difference between the Ad-TRβ PTU and Ad-TRβ T3 groups; ^∗^^/#^p < 0.05. (G and H) Liver ATF4 protein expression in mice due to PTU/T3 administration (G) (n = 4/group) or TRβ mutations (H) (n = 4–5/group). The asterisks indicate the significance of the difference between Hyper-T and Eu-T or Hypo-T (G), or WT and TRβ^PV/+^ or TRβ^PV/PV^ (H), and the # sign indicates the significance of the difference between TRβ^PV/+^ and TRβ^PV/PV^ (H), ^∗^^/#^p < 0.05. Error bars are ± SEM. See also Figure S5.

While T_3_ acts broadly in vivo through multiple TH receptors, its action in liver depends primarily on TH receptor β1 (TRβ1). To test if negative regulation of TSP expression and H_2_S production capacity by T_3_ is organ autonomous or the result of systemic T_3_ action, we took advantage of the T_3_ analog GC-1, which preferentially acts on the liver via its uptake and specificity for TRβ1. GC-1 predictably modulated known T_3_ target genes in the liver (Figure 5A, left) and negatively regulated components of the hepatic methionine cycle and TSP, including CGL and CBS gene and protein expression (Figures 5A and 5B). GC-1 also reduced hepatic H_2_S production capacity (Figure 5C) and altered hepatic TSP metabolites, including cystathionine, similar to T_3_ (Figure S5A).

The genetic requirement for TRβ1 in repression of hepatic TSP expression and H_2_S production was tested in mice with a mutant and defective TRβ1 isoform (TRβ^PV/PV^) (Zhao et al., 2012). TRβ^PV/PV^ mice had higher CGL and CBS protein levels in the liver and enhanced H_2_S production capacity than heterozygote or WT control animals (Figures 5D and 5E). Interestingly, neither CGL nor CBS mRNA levels were significantly altered in the TRβ^PV/PV^ mice compared with controls (Figure S5B), possibly due to long-term upregulation of this pathway in these animals. Thus, an intact TRβ regulates H_2_S production, but it also remains possible that TRα plays a role in TRβ^PV/PV^ mutants.

Finally, to test the hypothesis that TRβ1 regulates H_2_S production capacity by direct transcriptional control of TSP genes in vivo, we examined the TRβ cistrome that we generated previously (Ramadoss et al., 2014) for binding sites within regulatory regions of the CBS and CGL genes. Chromatin immunoprecipitation analysis revealed enrichment of TRβ binding at several loci on the CBS and CGL genes, as well as other genes of the methionine cycle (Figure 5F). Interestingly, T_3_ injection resulted in a significant reduction in occupancy of TRβ on CGL and Bhmt promoters/enhancers, but not CBS binding sites. While the detailed mechanisms of negative regulation of hepatic TSP gene expression by T_3_ and TRβ1 remain to be elucidated, these data suggest regulation of hepatic H_2_S production via TRβ-dependent repression of TSP gene expression.

In addition to negative regulation by T_3_/TRβ as described here, CGL gene expression is positively regulated by the stress response transcription factor ATF4 in reaction to cysteine restriction on the cell-autonomous level (Lee et al., 2008, Sikalidis et al., 2011) and in livers of mouse models of DR and Snell dwarfism (Li et al., 2014). Surprisingly, we found that reduction of global TH signaling via a PTU/LID diet (Figure 5G), or liver-specific signaling in TRβ^PV/PV^ mice (Figure 5H), increased hepatic ATF4 protein levels. Despite the increase in hepatic ATF4 protein in Snell dwarf mice (Figure S5C), we were not able to detect significantly elevated levels in GHRKO mice (Figure S5D). Thus, hyperthyroidism is associated with negative regulation of CGL expression through direct binding and repression of the CGL locus by T_3_-bound TRβ1, while hypothyroidism is associated with increased CGL expression indirectly through derepression of the transcriptional activator ATF4, possibly by hypothyroidism-induced endoplasmic reticulum stress (Zhou et al., 2016).

### Additive Suppression of Hepatic H_2_S Production by TH and GH In Vitro

**Figure 6:**
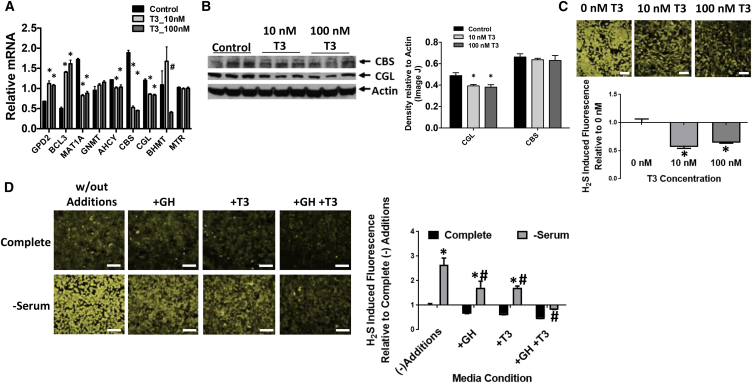
Additive Suppression of Hepatic H_2_S Production by TH and GH In Vitro (A–C) mRNA levels (n = 3) (A), protein expression (n = 3) (B), and endogenous H_2_S production (n = 3) (C) in TH-responsive Hepa1-6 cells treated with T3 at the indicated concentration. The asterisk indicates the significance of the difference between vehicle control and T3 treatment and the hash indicates the significance of the difference between different T3 dosage groups; ^∗^^/#^p < 0.05. (D) Endogenous H_2_S production in TH-responsive Hepa1-6 cells grown in Complete medium or −Serum medium +/−GH +/−T3. The asterisk indicates the significance of the difference between Complete and −Serum within the GH/T3 treatment group, and the # sign indicates the significance of the difference between “w/out Additions” and +GH, +T3, or +GH+T3 groups within the −Serum grouping; ^∗^/^#^p < 0.05. Scale bars, 100 μm (C and D). Error bars are ± SEM. See also Figure S6.

Having identified GH- and TH-dependent control of hepatic H_2_S production capacity, we next sought to determine the potential interaction between the two in a tractable in vitro system. Because primary murine hepatocytes do not respond well to T_3_, we employed a murine hepatic cell line, Hepa1-6, stably transfected with the TRβ isoform. Upon addition of T_3_ to this cell line, known T_3_ targets including gpd2 and bcl3 were induced, while methionine cycle genes mat1a and ahcy were repressed (Figure 6A). Importantly, both CGL and CBS were downregulated by T_3_ in these cells (Figure 6A), although only CGL was regulated in a similar fashion at the protein level (Figure 6B). Endogenous H_2_S production assessed using the P3 probe was reduced upon T_3_ addition, confirming its direct ability to regulate endogenous H_2_S through repression of CGL mRNA expression (Figure 6C).

We next tested the individual and combined contributions of GH and TH in regulation of endogenous H_2_S production in this cell line. Overnight serum withdrawal resulted in a 2.5-fold increase in endogenous H_2_S levels (Figure 6D). Addition of GH or TH individually each resulted in a 50% decrease in H_2_S levels, while together they restored H_2_S to baseline levels seen in cells maintained in full serum (Figure 6D). Finally, gene expression analysis confirmed the effects of TH, but not GH, on TSP gene expression (Figure S6). Taken together, GH and TH work additively to suppress hepatic H_2_S production in a cell-autonomous manner by distinct mechanisms.

### CGL/H_2_S Required for Negative Regulation of TH and GH/IGF-1 Signaling

**Figure 7:**
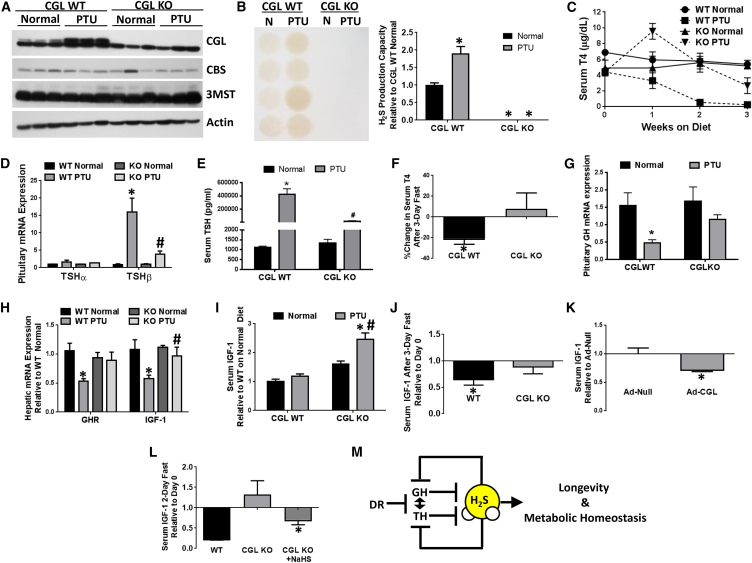
CGL/H_2_S Is Required for Negative Regulation of TH and GH/IGF-I Signaling (A and B) Western blot analysis of H_2_S-producing enzymes CGL, CBS, and 3MST (n = 3) (A) and H_2_S production capacity in livers of CGL WT and KO mice on Normal and PTU diets (n = 4–5) (B). The asterisk indicates the significance of the difference relative to CGL WT mice on the Normal diet; ^∗^p < 0.05. (C) Serum T4 levels over a 3-week time course of Normal versus PTU diets in CGL WT and KO mice as indicated (n = 4–5). (D) Pituitary mRNA expression of TSHα and TSHβ in CGL WT and KO mice after 3 weeks of Normal or PTU diet (n = 4–5). The asterisk indicates the significance of difference between diets within genotype, and the # sign indicates the significance of difference between CGL WT and CGL KO mice on the PTU diet; ^∗^/^#^p < 0.05. (E) Serum TSH levels after 3 weeks of Normal or PTU diet in CGL WT and KO mice (n = 4–5). The asterisk indicates the significance of difference between diets within genotype, and the # sign indicates the significance of difference between CGL WT and CGL KO mice on the PTU diet; ^∗^/^#^p < 0.05. (F) Percent change in serum T4 levels after 3 days of fasting in CGL WT and CGL KO mice (n = 3–4). The asterisk indicates the significance of the difference between day 0 and day 3 T4 levels; ^∗^p < 0.05. (G–I) Pituitary GH mRNA expression (n = 4–5) (G), liver GHR and IGF-I mRNA expression (n = 4–5) (H), and serum IGF-1 (n = 4–5) (I) in CGL WT and KO mice after 3 weeks on a Normal or PTU diet as indicated. The asterisk indicates the significance of the difference between diets within genotype, and the # sign indicates the significance of the difference between genotypes on the PTU diet; ^∗^^/#^p < 0.05. (J) Fold change in serum IGF-I between day 0 and day 3 of a 3-day fast in CGL WT and KO mice (n = 3–4/group). The asterisk indicates the significance of the difference between day 3 and day 0; ^∗^p < 0.05. (K) Serum IGF-1 levels in mice 7 days after adenoviral infection with Ad-Null control or Ad-CGL overexpression adenovirus expressed relative to Ad-Null control (n = 4/group). The asterisk indicates the significance of the difference between Ad-Null and Ad-CGL; ^∗^p < 0.05. (L) Fold change in serum IGF-1 after a 2-day fast in female CGL WT and KO mice with +/−NaHS supplementation in the CGL KOs (n = 4–5/group). The asterisks indicate the significance of the difference between serum IGF-1 levels on day 2 compared with day 0; ^∗^p < 0.05. (M) Relationship between diet, GH/TH, and H_2_S production. Error bars are ± SEM. See also Figure S7.

What are the functional consequences of increased H_2_S production in the context of reduced GH and/or TH signaling? We first approached this question by testing the genetic requirement for CGL in increased hepatic H_2_S production observed upon hypothyroidism. CGL protein and H_2_S production capacity were increased in hypothyroid WT mice on the PTU/LID diet compared with euthyroid WT mice on the normal diet, but were undetectable in littermate CGL KO mice on either diet (Figures 7A and 7B, and Figure S7A). No differences in food intake (Figure S7B), body weight (Figure S7C), or protein levels of the other H_2_S-producting enzymes CBS and 3-MST (Figures 7A and S7A) were observed between genotypes. We conclude that CGL is required for increased hepatic H_2_S production upon decreased TH signaling.

Surprisingly, CGL KO mice failed to achieve the same degree of hypothyroidism as WT controls on the PTU/LID diet. This was first observed systemically upon measuring circulating T_4_, which remained elevated in CGL KO mice on the PTU/LID diet (Figure 7C), and was confirmed by TSHβ subunit gene expression in the pituitary (Figure 7D) and circulating TSH in the serum (Figure 7E), both of which failed to increase in CGL KO mice to WT levels on the PTU/LID diet.

These data suggest a functional role for CGL-derived H_2_S in systemic feedback control of TH signaling, possibly through modulation of circulating T_4_ levels. To test this further, CGL KO and WT mice were subject to fasting, a known suppressor of the hypothalamic-pituitary-thyroid axis on a more rapid timescale than the PTU/LID paradigm (Vella et al., 2011). While serum T_4_ levels decreased in WT mice subject to a 3-day fast, T_4_ levels actually increased slightly upon fasting in CGL KO mice (Figure 7F), despite similar weight loss between genotypes (Figure S7D). Taken together, these data suggest reciprocal negative regulation of H_2_S production by TRβ-dependent repression of hepatic CGL gene transcription, and T_4_ metabolism by CGL-derived H_2_S.

We next considered the potential role of CGL-derived H_2_S in negative regulation of global and hepatic GH action. In CGL KO mice, expression of pituitary GH mRNA (Figure 7G), hepatic GHR and IGF-1 mRNAs (Figure 7H), and circulating IGF-1 levels (Figure 7I) were inappropriately maintained and/or elevated upon PTU/LID relative to WT mice. Similar to its effects on circulating T_4_, fasting significantly decreased serum IGF-1 in WT mice, but not in CGL KO mice (Figure 7J).

Finally, we tested the sufficiency of CGL-derived H_2_S or chemical H_2_S donors to lower GH signaling independent of PTU/LID or fasting. One week after adenoviral-mediated CGL overexpression in WT mice, which resulted in increased liver H_2_S production capacity (Figure S7E), serum IGF-1 levels were significantly reduced relative to the adenoviral null control (Figure 7K). In CGL KO mice subjected to a 2-day fast, intraperitoneal administration of NaHS rescued the expected drop in serum IGF-1 levels (Figure 7L) and serum T_4_ levels (Figure S7F). In WT mice, NaHS was sufficient to reduce circulating IGF-1 levels after acute injection (Figure S7G) or long-term treatment in the drinking water in combination with the slow-releasing H_2_S donor GYY4137 (Lee et al., 2011) (Figure S7H). These findings are consistent with a role for CGL-derived endogenous H_2_S in negative regulation of GH and TH signaling.

## Discussion

### H_2_S as a Common Endpoint in Models of Longevity and Metabolic Homeostasis

Reduced GH/IGF-1 action and DR represent the most widely studied classes of anti-aging models in rodents, but mechanisms underlying pleiotropic benefits on lifespan, healthspan, and stress resistance in these models, and the relationship between them, remain unclear. Previously we found that increased endogenous H_2_S production upon DR is necessary and sufficient for surgical stress resistance in mice, and associated with extended longevity in fly, worm, and yeast models (Hine et al., 2015). Here, we found that increased endogenous hepatic H_2_S production capacity was common to multiple long-lived mouse strains. As shown in the model in Figure 7M, GH and TH signaling were identified as negative regulators of hepatic H_2_S production. Unexpectedly, CGL was required for downregulation of T_4_ and IGF-1 in response to PTU/LID or fasting. Taken together, these data are consistent with increased hepatic H_2_S as a common denominator among DR and reduced GH/IGF-1-based pro-longevity models. Further, it points to negative regulation of TH and GH/IGF-1 signaling as a potential mechanism of H_2_S action in this context.

A limitation of this study lies in the quantitation of H_2_S levels. Much of the data was based on H_2_S production capacity using the lead acetate method, a measure of H_2_S production under conditions in which exogenous substrate (L-Cys) and co-factor (VitB6) are supplied in excess. While specific for H_2_S and sensitive for use in organ extracts with high production capacity, the more sensitive P3 fluorescent probe was required for measuring H_2_S production in individual cells in tissue culture or frozen liver sections. Importantly, the P3 probe reports on free H_2_S either generated de novo or released from sulfane sulfur without the addition of substrate or co-factor, thus making it possible to probe actual H_2_S production with endogenous substrates. While neither method is suited for absolute quantitation of H_2_S, both techniques showed increases in liver of Snell dwarf mice versus WT controls. Finally, while the importance of CGL-derived H_2_S was addressed by exogenous H_2_S supplementation in the context of CGL KO mice, our data in no way rule out potential contributions of other CGL-dependent metabolites such as glutathione.

### Interactions between Nutrient Restriction, Endocrine Signaling, and H_2_S Production

DR benefits overlap those of reduced GH/TH signaling, and indeed DR has been proposed to work, at least partially, the hypothalamic-pituitary axis, through modulation of somatotropic cells (Brown-Borg, 2015). The ability of DR to further extend longevity or improve metabolic fitness is blunted in most, but not all (Bartke et al., 2001), hypopituitary or GHRKO mouse studies (Arum et al., 2009, Bonkowski et al., 2006, Brown-Borg et al., 2014). In addition, GH supplementation concurrent with DR reverses some DR-related metabolic effects (Gesing et al., 2014). Hormonally, DR alters hypothalamic activity (Dacks et al., 2013), lowers GH (Fontana et al., 2008) and TH secretion (Fontana et al., 2006, Miller et al., 2005), and reduces hepatic GHR expression (Dauncey et al., 1994).

In addition to overlap between DR and reduced TH/GH signaling, genetic models of extended longevity associated with decreased GH/IGF-1 signaling also have decreased TH activity (Gesing et al., 2012). Conversely, reduction of TH signaling results in decreased GHR production and signaling (Dieguez et al., 1986). Re-addition of these hormones into hypopituitary dwarf mice individually or in combination reverses many of the associated phenotypes (Do et al., 2015, Panici et al., 2010).

Despite these interconnections, here we found distinct, additive contributions of TH and GH to regulation of H_2_S production independent of diet. TH directly regulated CGL and CBS gene expression via TRβ acting as a transcriptional repressor, while GH via GHR controlled H_2_S production in an autophagy-dependent manner suggestive of substrate-level control. In addition, TH negatively regulated protein levels of the ATF4 transcription factor, a known direct activator of CGL expression (Mistry et al., 2016). Future experiments are required to determine the importance of these mechanisms to overall regulation of H_2_S production and the contribution of increased H_2_S to the individual and shared phenotypes associated with DR and reduced GH and/or TH signaling. Together, they represent potential targetable pathways toward harnessing endogenous H_2_S production for beneficial outcomes.

### Inhibition of TH and GH Activity as a Novel Mechanism of H_2_S Action

Reduced insulin/IGF-1 signaling, often accompanied by reduced circulating levels of these hormones and increased sensitivity to their action, is a feature shared by multiple rodent models of extended longevity. Our finding that H_2_S is required in certain contexts for downregulation of IGF-1 production suggests control of IGF-1 as a novel mechanism by which H_2_S can exert its pleiotropic effects on health and longevity.

By what mechanism could H_2_S control hepatic IGF-1 production? The observation that CGL was required for lowering circulating levels of T_4_ and IGF-1 in two different models of hypothyroidism, combined with the ability of TH to activate GHR/IGF-1 gene expression (Koenig et al., 1987, Tsukada et al., 1998), suggests that the compromised ability to reduce circulating T_4_ may be partially responsible. Consistent with the potential of H_2_S to affect TH, H_2_S donors present in garlic suppress TH signaling in rats (Tahiliani and Kar, 2003), and addition of NaHS blocks the production and/or secretion of GH from pituitary-derived cells (Mustafina et al., 2015). Similarly, we found that increased CGL expression and/or exogenous H_2_S addition were sufficient to decrease circulating TH and IGF-1 in vivo (Figures 7 and S7).

Finally, we note that while a reduction in circulating IGF-1, which is produced mainly by liver, correlates with extended longevity, hepatic IGF-1 expression (and hence circulating IGF-1) can be reduced without longevity benefits, as in liver-specific IGF-1KO mice (Yakar et al., 1999). Future studies are required to determine the relative importance of hepatic H_2_S production on lifespan, as well as the effects of TH/GH signaling on H_2_S production in other tissue types.

### Implications for Human Health

While GH and TH signaling both decrease with age, it remains unclear if these changes are adaptive or maladaptive, and if preventing this decline alters healthy aging. GH supplementation results in increased lean body mass, decreased fat mass, and gains in muscle strength. However, it is also associated with edema, carpal tunnel syndrome, joint pain, and an increase in type 2 diabetes and glucose intolerance (Liu et al., 2007). Increased TH results in DNA damage and premature senescence in vitro and in vivo (Zambrano et al., 2014). Here we show that GH and TH supplementation suppresses H_2_S production. Because H_2_S is positively correlated with improved stress resistance and health, our findings raise concerns about the use of GH and/or TH supplementation as anti-aging or performance-enhancing therapies.

### Conclusions

Previously we found that increased endogenous H_2_S production is in part responsible for the pleiotropic effects of DR. Here we found that TH and GH, two endocrine hormones associated with longevity control, are regulators of hepatic H_2_S production. TH and GH independently and additively suppressed H_2_S production through inhibition of CGL gene expression and control of substrate availability via autophagy, respectively. Unexpectedly, CGL-dependent H_2_S itself was required for feedback regulation of TH signaling via negative regulation of T_4_ levels and GH signaling through negative regulation of IGF-1. Together, these data point to H_2_S as a potential downstream mediator of benefits shared between decreased GH/TH signaling and DR.

## STAR★Methods

### Key Resources Table

**Table:** 

REAGENT or RESOURCE	SOURCE	IDENTIFIER
**Antibodies**		

Anti-CGL (Anti-Cystathionase)	Abcam	Ab151769
Anti-CBS	Abcam	Ab135626
Anti-3MST (Anti-MPST)	Sigma	HPA001240
Anti-Stat5	Santa Cruz	Sc-835
Anti-p-Stat5	Cell Signaling Technology	9359
Anti-GNMT	Aviva	ARP43565_P050
Anti-AHCY	Abcam	Ab56146
Anti-ATF4 (Anti-CREB-2)	Santa Cruz	Sc-200
Anti ATG5	Novus	NB110-53818
Anti ATG7	Sigma	A2856
Anti-beta Tubulin	Cell Signaling	2128
Anti-Actin	Cell Signaling	4970
HRP conjugated anti-rabbit	Dako	P044801-2

**Bacterial and Virus Strains**		

Ad-CMV-CGL (Ad-mCTH)	Vector Biolabs	ADV-256305
Ad-CMV-Null	Vector Biolabs	1300
Lentiviral sh-GFP	Laboratory of Dr. Alec Kimmelman	N/A
Lentiviral sh-ATG5	Laboratory of Dr. Alec Kimmelman	N/A
Lentiviral sh-ATG7	Laboratory of Dr. Alec Kimmelman	N/A

**Biological Samples**		

Livers (frozen) taken from experimental mouse strains listed in the Experimental Models: Organisms/Strains section	See Experimental Models: Organisms/Strains section	See Experimental Models: Organisms/Strains section
Serum/Plasma (frozen) taken from experimental mouse strains listed in the Experimental Models: Organisms/Strains section	See Experimental Models: Organisms/Strains section	See Experimental Models: Organisms/Strains section

**Chemicals, Peptides, and Recombinant Proteins**		

NaHS	Sigma	161527
GYY4137	Sigma	SML0100
Lanreotide	Sigma	SML0132
PTU/LID diet	Harlan Teklad	TD 95125
T3 (Triiodo-L—thyronine)	Sigma	T2752
GC-1	Laboratory of Dr. Thomas Scanlan	Chiellini et al., 1998
GH (growth hormone)	Sigma	S8648
FGF21	Genscript	Z03290
Bafilomycin	Sigma	B1793
Chloroquine	Sigma	C6628
DL-Propargylglycine	Sigma	P7888
Aminooxyacetic acid	Sigma	C13408
AZD1480	Selleckchem	S2162
Passive Lysis Buffer (5x)	Promega	E1941
PLP (Pyridoxal 5′-phosphate)	Sigma	P9255
L-cysteine	Sigma	C7352
lead (II) acetate trihydrate	Sigma	316512
P3 H2S Detection Probe	From the lab of Prof. K.H. Ahn	Singha et al., 2015

**Critical Commercial Assays**		

Mouse/Rat IGF-1 ELISA kit	R&D Systems	SMG100
T4 ELISA kit	Diagnostic Automation/ Cortez Diagnostics, Inc	3149-18
MILLIPLEX MAP Mouse Endocrine (TSH) Assay	EMD Millipore	MPTMAG-49K

**Experimental Models: Cell Lines**		

Hepa1-6 2Cl BirA/TRbeta	Laboratory of Dr. Anthony N. Hollenberg	N/A
Hepa1-6 (mouse liver hepatoma)	ATCC	CRL-1830
Primary mouse hepatocytes prepared from C57BL/6 mice (freshly isolated in the lab of Dr. James Mitchell for each experiment)	Jackson Laboratories and laboratory of Dr. James R. Mitchell	000664 and this paper
ATG5 Knockout mouse embryonic fibroblasts	From the laboratory of Dr. Gokhan Hotamisligil	Yang et al., 2010
ATG7 Knockout mouse embryonic fibroblasts	From the laboratory of Dr. Gokhan Hotamisligil	Yang et al., 2010
Primary CGL WT and KO mouse tail dermal fibroblasts	From the laboratory of Dr. James R. Mitchell	This paper

**Experimental Models: Organisms/Strains**		

129/C57BL/6 background WT and KO CGL Male and Female Mice	Laboratories of Dr. Rui Wang and Dr. James R. Mitchell	Hine et al., 2015, Yang et al., 2008
WT and LirKO Female Mice	Laboratory of Dr. James R. Mitchell	Harputlugil et al., 2014
Male and Female WT and Snell Dwarf mice	Laboratory of Dr. Richard Miller	Dozmorov et al., 2002
Female WT and Ames Dwarf mice	Laboratory of Dr. Andrzej Bartke	Panici et al., 2010
Male and Female WT and GHRKO Mice	Laboratory of Dr. Richard Miller	Wang and Miller, 2012
Male and Female WT and IRS-1 KO mice	Laboratory of Dr. Colin Selman	Selman et al., 2008
Male WT and FGF21 overexpressing mice	Laboratory of Dr. Pavlos Pissios	Kharitonenkov et al., 2005
TRbeta NI/NI, NI/PV, and mutant PV/PV mice	Laboratory of Dr. Sheu-yann Cheng	(Kato et al., 2004
C57BL/6 mice	Jackson Laboratories	000664
B6D2F1 hybrid mice	Jackson Laboratories	100006

**Oligonucleotides**		

IGF-1 F: TGCTTGCTCACCTTCACCA IGF-1 R: CAACACTCATCCACAATGCC	N/A	N/A
GHR F: ATTCACCAAGTGTCGTTCCC GHR R: TCCATTCCTGGGTCCATTCA	N/A	N/A
CGL F: TTGGATCGAAACACCCACAAA CGL R: AGCCGACTATTGAGGTCATCA	N/A	N/A
CBS F: GGGACAAGGATCGAGTCTGGA CBS R: AGCACTGTGTGATAATGTGGG	N/A	N/A
HPRT F:TTTCCCTGGTTAAGCAGTACAGCCC HPRT R:TGGCCTGTATCCAACACTTCGAGA	N/A	N/A
RPL13 F:TTCGGCTGAAGCCTACCAGAAAGT RPL13 R:TCTTCCGATAGTGCATCTTGGCCT	N/A	N/A
MAT1A F: GATAGCAGATCTGAGGCGCT MAT1A R: TGCACCATTATCCTGCATGT	N/A	N/A
GNMT F: AAGAGGGCTTCAGCGTGATG GNMT R: CTGGCAAGTGAGCAAAACTGT	N/A	N/A
AHCY F: CGCCAGCATGTCTGATAAAC AHCY R: CCTGGCATCTCATTCTCAGC	N/A	N/A
BHMT F: TTAGAACGCTTAAATGCCGGAG BHMT R: GATGAAGCTGACGAACTGCCT	N/A	N/A
*For a full list of all primers used, please see*Table S1		

**Software and Algorithms**		

ImageJ	National Institutes of Health	Windows version, https://imagej.nih.gov/ij/download.html

### Contact for Reagent and Resource Sharing

Further information and requests for resources and reagents should be directed to and will be fulfilled by the Lead Contact, James R. Mitchell (jmitchel@hsph.harvard.edu).

### Experimental Models

#### In Vivo Animal Studies

All experiments were performed with the approval of the Institutional Animal Care and Use Committee (IACUC) from the respective institutions. Except where indicated in this paper or cited in the respective references, animals were bred and maintained under standard housing conditions with *ad libitum* access to food (Purina 5058) and water, 12-hour light/12-hour dark cycles, temperature between 20–23°C with 30%–70% relative humidity, and weaned from their mothers between 3-4 weeks of age. Mice used included: young adult (10-15 week old) male and (1-year old) female CGLKO and WT control mice on a mixed 129/C57BL/6 background (Hine et al., 2015, Yang et al., 2008); female (10-16 week old) LIrKO and control mice generated by crossing *Ir*^*fl/fl*^ (WT) mice with *Ir*^*fl/fl*^*|Albumin-Cre*^*+/−*^ (LIrKO) mice as previously described (Harputlugil et al., 2014); male and female Snell Dwarf and WT littermates aged 4-5 months (Dozmorov et al., 2002); 18-month female Ames Dwarf and WT littermates treated daily with recombinant growth hormone during a 6-week period early in life (weeks 2-8) as described (Panici et al., 2010), male and female GHRKO and WT littermates aged 7-8 months (Wang and Miller, 2012), sixteen week old male and female IRS-1 KO and WT littermates (Selman et al., 2008); male FGF21 overexpressing (OE) transgenic and WT littermates (Kharitonenkov et al., 2005) and TRβ WT (NI/NI), heterozygote (NI/PV) and Mutant (PV/PV) mice as previously described (Kato et al., 2004).

Lanreotide (Sigma) was administered to 10 week old male WT B6D2F1 hybrids (Jackson Labs) via daily sub-cutaneous injection at 0.4 mg/kg (Fan et al., 1996) in sterile saline once/day for 7 days prior to euthanasia and organ harvest. CGL was overexpressed by IV injection of 10^10^ PFUs of Ad-CMV-CGL (ADV-256305) or control Ad-CMV-Null virus (Vector Biolabs) into 10 week old male WT B6D2F1 hybrids as previously described (Hine et al., 2015) 7 days prior to blood serum collection for IGF-1 determination. Hydrogen sulfide supplementation was performed in male C57BL/6 mice (Jackson) using NaHS (Sigma) (1mM) and GYY4137 (Sigma) (260μM) in the drinking water starting at 10 weeks of age for 2 weeks, with supplementation of additional NaHS every two days and GYY4137 after the first week and with blood taken for analysis on day 0, 7, and 14. Young adult (10 to 15 weeks of age) male CGL WT and KO mice were fasted of food for three days with *ad libitum* access to drinking water and blood/serum sampled at Day 0 and Day 3. 1-year old female CGL WT and KO mice were fasted of food for two-days and given *ad libitum* access to drinking water, and then blood taken on Day 0 and Day 2, with one of the two CGL KO groups receiving NaHS injection in sterile saline at a dose of 5mg/kg per injection after the first blood draw on day 0, day 1, and just prior to harvest on day 2. 10-week old male B6D2F1 hybrids were kept on an *ad libitum* rodent diet and injected with NaHS in sterile saline at 5mg/kg and blood/serum taken 24-hours later for analysis. Recombinant human IGF-1 (rhIGF-1) at 500 μg/kg/d or recombinant human growth hormone (rhGH) at 2 mg kg/day (BID) were administered by intraperitoneal injection for 20 days in 12-week-old male C57BL/6 mice as described (Lee et al., 2014). Modulation of thyroid hormone levels on a hypothyroid background was accomplished in 9-week old male C57BL6 mice maintained for 3 weeks on a PTU/LID diet (Harlan Teklad formula # TD 95125) to induce hypothyroidism followed by saline or T3 (Sigma, St. Louis, MO) intraperitoneal injection once a day for 4 days to create hypothyroid (saline), euthyroid (0.5 μg/100g bodyweight of T3) or hyperthyroid (25 μg/100g bodyweight of T3) mice. For modulation of thyroid hormone levels on a euthyroid background, 10 week old male mice fed a standard rodent diet (Labdiet Chow) were injected intraperitoneally with saline or 25 μg/100g bodyweight of T3 once a day for 4 days. GC-1 (Chiellini et al., 1998) was administered to 10-week old male C57BL6 mice fed a standard rodent diet (Labdiet Chow) by intraperitoneal injection (3 μg/100g bodyweight) once a day for 4 days.

#### Cell Lines and In Vitro Tissue Culture Studies

Mouse primary hepatocytes from 8-10 week old female WT C57BL/6 mice (Jackson Laboratories) were isolated via portal vein collagenase treatment (Liberase, Roche) followed by Percoll gradient centrifugation and culturing in William’s E media with 5% FBS at 37°C, 20% O_2_ and 5% CO_2_. The Hepa1-6 cell line, originally obtained from a C57L mouse (sex unknown), was maintained in DMEM media with 10% FBS. Hepa1-6 2Cl BirA/TRβ cells, which were generated by sequentially transfecting BirA And TRβ into Hepa 1-6 cells and selecting clones using geneticin and puromycin, were maintained in DMEM+Glutamax with 10% vol/vol FBS supplemented with Gibco Antibiotic-Antimycotic, Geneticin, and Puromycin at 37°C, 20% O_2_ and 5% CO_2_. Lentiviral infections of Hepa1-6 cells with sh-GFP, sh-ATG5 or sh-ATG7 were performed in the presence of 10% FBS and 8μg/ml Polybrene on two consecutive days; infected Hepa1-6 cells were selected with 3μg/ml puromycin for 2 weeks before use and maintained with 3μg/ml puromycin. Genetically deficient ATG5 fibroblasts originally from 129/C57BL mixed background embryos (sex unknown) and ATG7 fibroblasts originally from C57BL/6 embryos (sex unknown) were a gift from the lab of Dr. Gokhan Hotamisligil and previously reported (Yang et al., 2010). CGL WT and KO mouse dermal fibroblasts from tail skin were prepared from female CGL WT and KO mice and maintained in 20% FBS in DMEM +Penicillin/Streptomycin. For differential H_2_S determination, an equal number of cells were seeded onto 12-well plates and incubated overnight in media +/- serum with or without the following drugs/hormones: GH (0.1-1μg/mL, Sigma), FGF21 (100nM, Genscript), T3 (10&100nM, Sigma), Bafilomycin (1μM, Sigma), Chloroquine (10μM, Sigma), DL-Propargylglycine (PAG) (100μM, Sigma), Aminooxyacetic acid (AOAA) (100μM, Sigma), AZD1480 (10μM, Selleckchem).

### Method Details

#### Lead Sulfide Method for Determination of H_2_S Production Capacity

H_2_S production capacity in liver homogenates was measured as previously described (Hine et al., 2015). Briefly, fresh or flash frozen liver was homogenized in passive lysis buffer (Promega) and volume normalized to protein content. An equal volume/protein amount was added to a reaction master mix containing PBS, 1mM Pyridoxal 5′-phosphate (PLP) (Sigma) and 10mM Cys (Sigma) and placed in a well-format (12-well to 96-well) plate. H_2_S detection paper, saturated with lead acetate and then dried, was placed above the plate and incubated 1-2hrs at 37°C until H_2_S in the gas phase reacted with the paper to form dark lead sulfide. In live primary hepatocytes, growth media was supplemented with 10mM Cys and 10μM PLP, and a lead acetate H_2_S detection paper placed over the plate for 2-24hrs at 37°C in a CO_2_ incubator.

#### H_2_S Detection with Fluorescent Probe

##### Cultured Cells

The chemical H_2_S probe P3 (Singha et al., 2015) was added at 10μM final concentration directly to the cell culture media following overnight treatment of cells (e.g. +/- serum, +/- hormones/drugs). One hour after P3 addition, H_2_S-activated P3 fluorescence was quantitated on a BioTek plate reader (excitation 360nm, emission 528nm). Alternately, cells were washed 1x with PBS, fixed in ice-cold methanol for 10 minutes, dried, and stored at -20°C until imaging via 2-photon microscopy (Mai Tai, Spectra-Physics) with excitation at 880 nm and emission 520-530nm using a 10x objective following rehydration in 1x PBS. Images were analyzed using the Integrated Density (IntDen) function of ImageJ software to determine the total florescence of the entire image.

##### Liver *Ex Vivo* Analysis

Flash frozen liver from adult male WT or Snell Dwarf mice was embedded in OCT media and cryosectioned onto glass slides, thawed to room temp and incubated in PBS containing 10μM P3 at room temperature for 30 minutes prior to fixation and then subsequent imaging via 2-photon microscopy using a 60x objective. The fluorescence signal was determined using the Integrated Density (IntDen) function of ImageJ obtained from analyzing the entire image and averaging three images per animal and three animals/group. These values were then normalized to the average wildtype control value (set to 1).

#### PTU/LID Diet for Altering Thyroid State

10-15 week old WT or CGLKO male mice were given *ad libitum* access to the Normal control 5058 diet (Purina), or the low iodine diet with 0.15% PTU; PTU/LID (Harlan Teklad) for three weeks prior to assay or harvest.

#### Western Blots/Protein Analysis

Protein analysis was performed via western blot on tissue and cell homogenates in passive lysis buffer (Promega), separated by SDS-PAGE, transferred to PVDF membrane (Whatman) and blotted for CGL (ab151769 Abcam), CBS (ab135626 Abcam), 3MST (HPA001240 Sigma), Stat5 (sc-835 Santa Cruz), p-Stat5 (#9359 Cell Signaling Technology), GNMT (Aviva), AHCY (Abcam ab56146), ATF4 (aka CREB-2 C-20, Santa Cruz Biotechnology sc-200), ATG5 (Novus, NB110-53818), ATG7 (Sigma, A2856), β-Tubulin 9F3 (#2128 Cell Signaling) or Actin (#4970 Cell Signaling) followed by HRP conjugated secondary anti-rabbit antibody (Dako).

#### qPCR/mRNA Analysis

Total RNA was isolated from tissues and cells using standard phenol-chloroform/isopropanol extraction and cDNA synthesized by random hexamer priming with the Verso cDNA kit (Thermo). qRT-PCR was performed with SYBR green dye (Lonza) and TaqPro DNA polymerase (Denville) or Taqman Universal PCR Master Mix (Thermo Fisher Scientific). Fold changes were calculated by the ΔΔC_t_ method using the genes Hprt and/or Rpl13 as controls and ultimately normalized to the control for each respective experiment, or normalized to a standard curve utilizing cyclophilin as an internal control. Primer sets used for PCR are listed in the Key Resources Table and in Table S1.

#### ChIP of TRβ

TRβ1-binding sites in gnmt, cth, cbs, mat1a and bhmt were identified in a previous study that characterized the cistrome of TRβ1 in mouse liver (Ramadoss et al., 2014). ChIP-qPCR was performed as previously described. Briefly, hypothyroid mice that expressed BirA ubiquitously were transduced with an adenovirus expressing wither GFP alone or Blrp-TRβ1 and GFP together. Subsequently half the hypothyroid mice in each group were given T3 injections to render them hyperthyroid. Livers from these mice were collected for chromatin affinity precipitation using streptavidin-agarose beads and qPCR was performed using primer sequences directed against genomic sites identified by ChIP-seq analysis.

#### Liver Metabolomics

*Ex vivo* mouse liver polar metabolomics was performed using targeted tandem mass spectrometry (LC-MS/MS) with polarity switching and selected reaction monitoring (SRM) with a AB/SCIEX 5500 QTRAP Mass spectrometer as previously described in (Yuan et al., 2012).

#### Detection of Serum Hormones: IGF-1, T4, TSH

Serum IGF-1 was detected using the IGF-1 Mouse/Rat ELISA kit (R&D Systems) following the manufacturer’s recommendations. Total plasma T4 levels were measured using a commercially available ELISA Kit (Diagnostic Automation/ Cortez Diagnostics, Inc Calabasas, CA). Thyroid-stimulating hormone (TSH) was measured in plasma via Milliplex MAP (mutianalyte panels) (mouse thyroid hormone TSH panel; EMD Millipore, Billerica, MA).

### Quantification and Statistical Analysis

Data are displayed as means +/- standard error of the mean (SEM) and statistical significance assessed in GraphPad Prism and/or Microsoft Excel using Student’s t tests to compare values between two specific groups, and one-way or two-way ANOVA followed by Tukey’s or Sidak’s Multiple Comparisons Tests when comparing more than two groups/variables at a given time. A P-value of 0.05 or less was deemed statistically significant in all of these statistical tests. Statistical details and results of experiments are found in the figures and figure legends. Quantification of western blot images, lead sulfide H_2_S production capacity assays, and 2-photon H_2_S production images was done using the IntDen measurement in ImageJ software and normalized to the respective control group in each experiment when applicable. All experiments examining hepatic H_2_S production were initially performed in a blinded fashion and technical repeats were done at least twice. Snell Dwarf and GHRKO experiments were repeated twice independently for a total of 6/genotype per sex or 12 total/genotype, and PTU/LID diet experiments in CGL WT and KO mice were repeated independently three times for a total of 11-12 animals/group.

## Author Contributions

C.H., H.K., Y.Z., E.H., A.L., M.S.M., P.R., K.T.B., J.M.A., R.M., P.P., K.Y., V.L., P.C., A.B., R.M., J.R.M., and A.N.H. designed and/or performed experiments and analyzed data. J.M.A., C.K.O., S.C., S.S., K.H.A., A.K., F.M.F., P.P., D.J.W., C.S., R.W., K.Y., V.L., P.C., A.B., J.J.K., and R.M. contributed material resources. C.H., H.K., Y.Z., E.H., J.R.M., and A.N.H. wrote the manuscript. J.M.A., F.M.F., C.S., V.L., P.C., A.B., J.J.K., and R.M. edited the manuscript.

## Supplemental Information

Document S1. Figures S1–S7 and Tables S1

Document S2. Article plus Supplemental Information
