# Supplementary material for: Hypothalamic-Pituitary Axis Regulates Hydrogen Sulfide Production
Source: Cell Metab. 2017 Jun 6;25(6):1320–1333.e5. doi: 10.1016/j.cmet.2017.05.003 (PMC5722247; doi:10.1016/j.cmet.2017.05.003)
Supplement: Document S1. Figures S1–S7 and Tables S1 [file mmc1.pdf]

Cell Metabolism, Volume *25*

Supplemental Information

Hypothalamic-Pituitary Axis Regulates Hydrogen Sulfide Production

Christopher Hine, Hyo-Jeong Kim, Yan Zhu, Eylul Harputlugil, Alban Longchamp, Marina Souza Matos, Preeti Ramadoss, Kevin Bauerle, Lear Brace, John M. Asara, C. Keith Ozaki, Sheue-yann Cheng, Subhankar Singha, Kyo Han Ahn, Alec Kimmelman, Ffolliott M. Fisher, Pavlos Pissios, Dominic J. Withers, Colin Selman, Rui Wang, Kelvin Yen, Valter D. Longo, Pinchas Cohen, Andrzej Bartke, John J. Kopchick, Richard Miller, Anthony N. Hollenberg, and James R. Mitchell

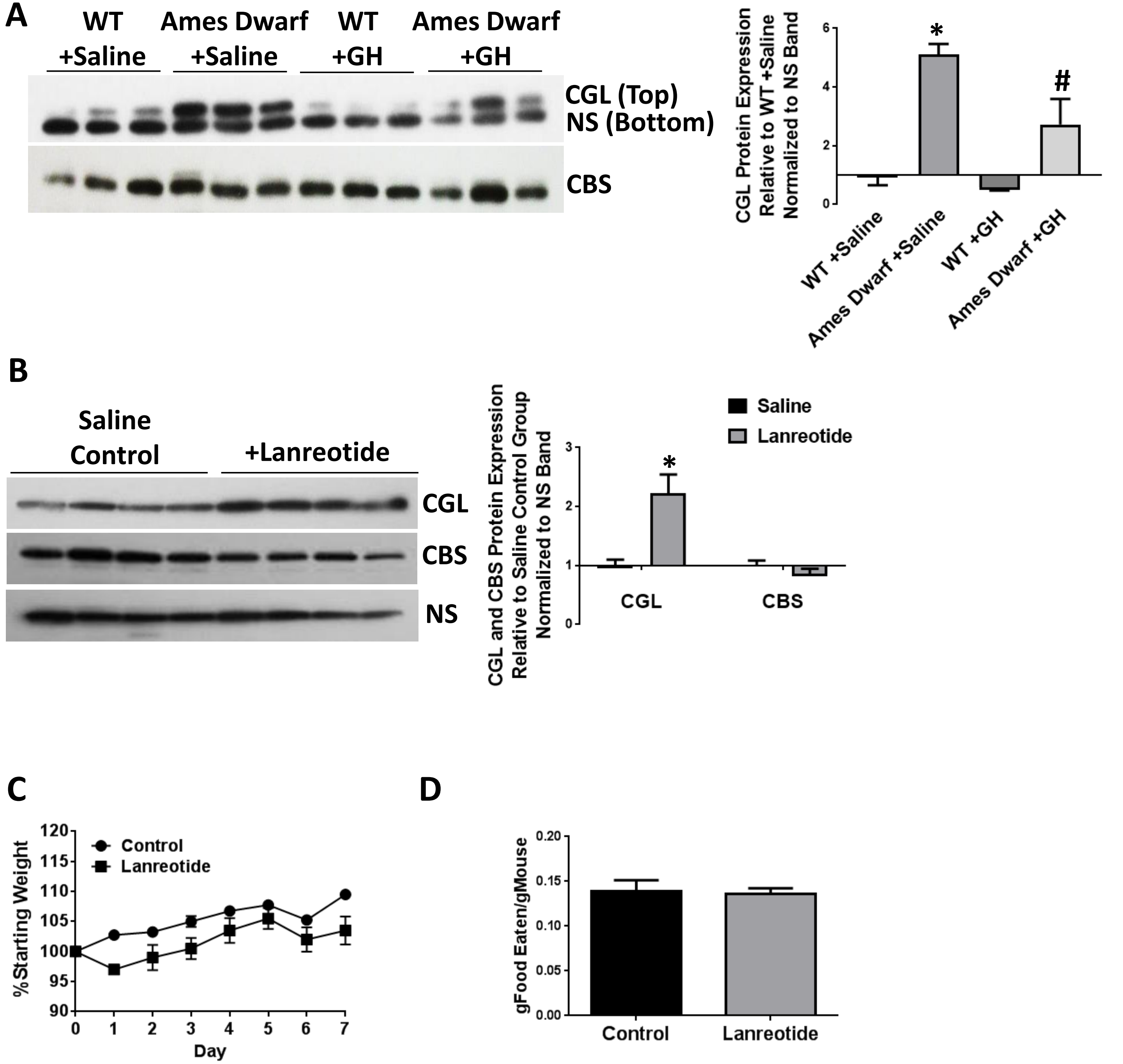
A
WT +Saline
Ames Dwarf +Saline
WT +GH
Ames Dwarf +GH
CGL (Top)
NS (Bottom)
CBS
CGL Protein Expression Relative to WT +Saline Normalized to NS Band
B
Saline Control
+Lanreotide
CGL
CBS
NS
Saline
Lanreotide
CGL and CBS Protein Expression Relative to Saline Control Group Normalized to NS Band
C
Control
Lanreotide
%Starting Weight
Day
D
gFood Eaten/gMouse
Control
Lanreotide

Figure S1

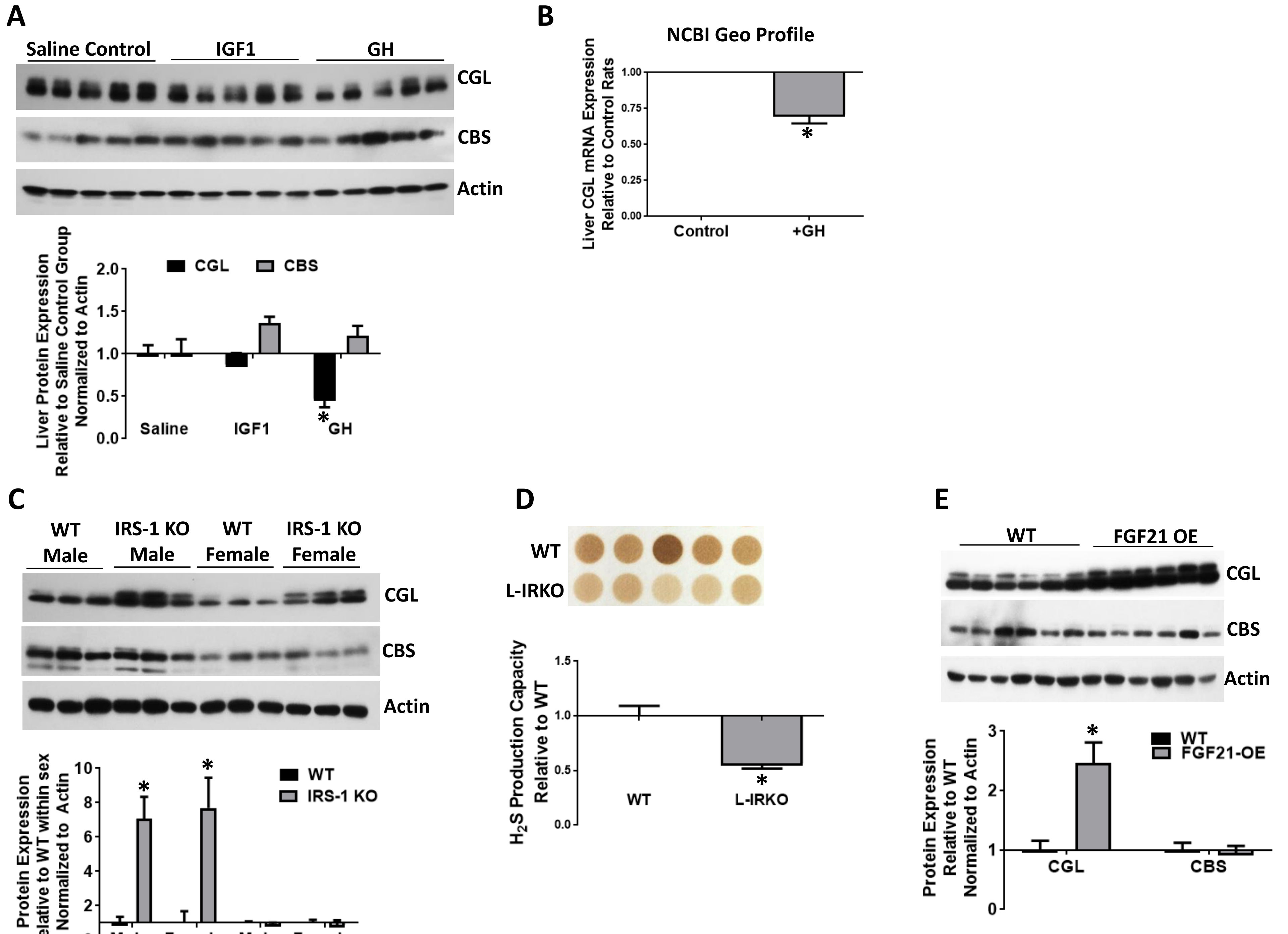
A
Saline Control
IGF1
GH
CGL
CBS
Actin
Liver Protein Expression Relative to Saline Control Group Normalized to Actin
CGL
CBS
Saline
IGF1
GH
B
NCBI Geo Profile
Liver CGL mRNA Expression Relative to Control Rats
Control
+GH
C
WT Male
IRS-1 KO Male
WT Female
IRS-1 KO Female
CGL
CBS
Actin
Protein Expression Relative to WT within sex Normalized to Actin
WT
IRS-1 KO
Male CGL
Female CGL
Male CBS
Female CBS
D
WT
L-IRKO
H2S Production Capacity Relative to WT
WT
L-IRKO
E
WT
FGF21 OE
CGL
CBS
Actin
Protein Expression Relative to WT Normalized to Actin
WT
FGF21-OE
CGL
CBS

Figure S2

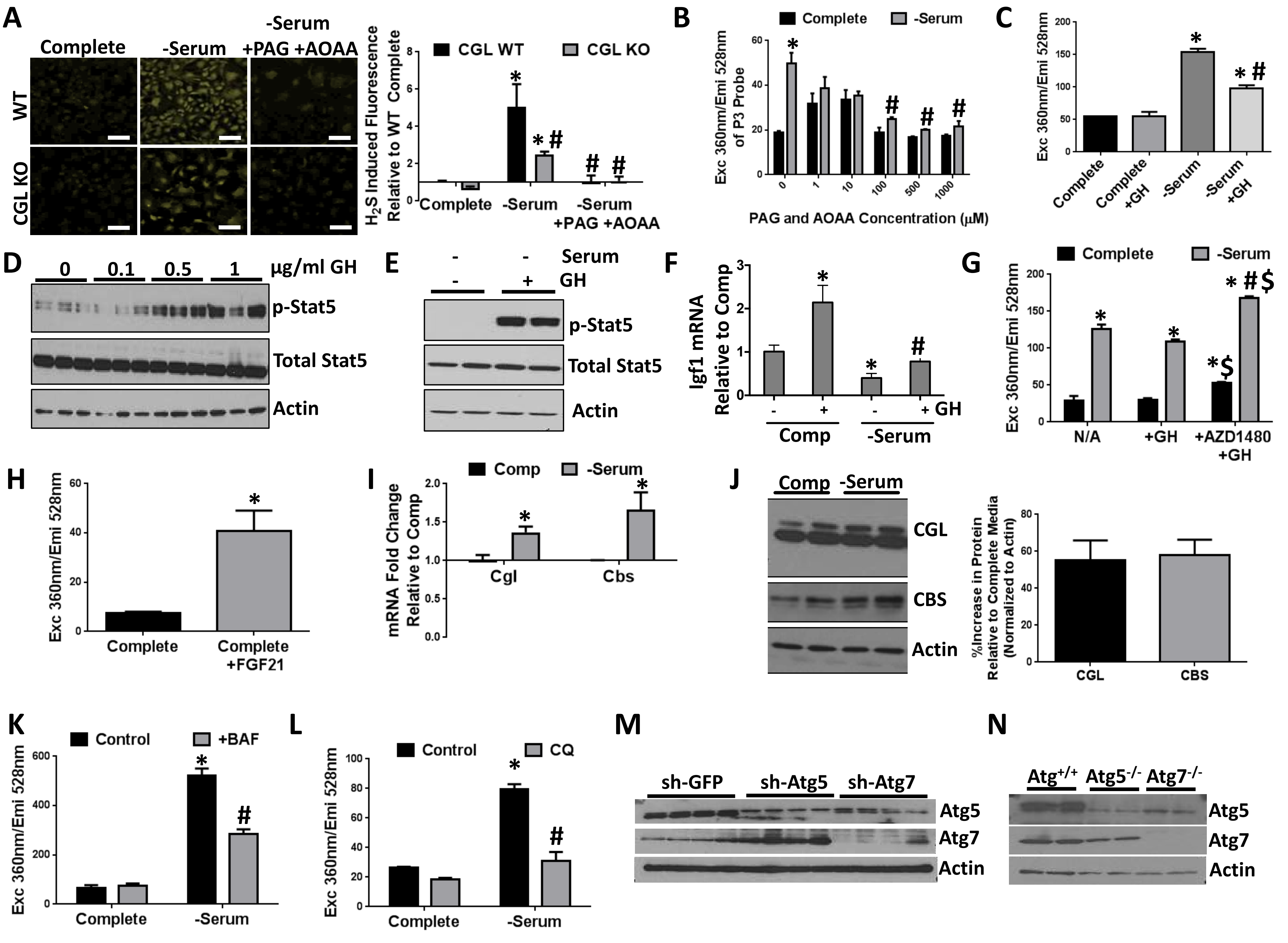

A
Complete
-Serum
-Serum +PAG +AOAA
WT
CGL KO
H2S Induced Fluorescence Relative to WT Complete
CGL WT
CGL KO
Complete
-Serum
-Serum +PAG +AOAA
B
Complete
-Serum
Exc 360nm/Emi 528nm of P3 Probe
PAG and AOAA Concentration (μM)
C
Exc 360nm/Emi 528nm
Complete
Complete +GH
-Serum
-Serum +GH
D
μg/ml GH
p-Stat5
Total Stat5
Actin
E
Serum
GH
p-Stat5
Total Stat5
Actin
F
Igf1 mRNA Relative to Comp
GH
Comp
-Serum
G
Complete
-Serum
Exc 360nm/Emi 528nm
N/A
+GH
+AZD1480 +GH
H
Exc 360nm/Emi 528nm
Complete
Complete +FGF21
I
Comp
-Serum
mRNA Fold Change Relative to Comp
Cgl
Cbs
J
Comp
-Serum
CGL
CBS
Actin
%Increase in Protein Relative to Complete Media (Normalized to Actin)
CGL
CBS
K
Control
+BAF
Exc 360nm/Emi 528nm
Complete
-Serum
L
Control
CQ
Exc 360nm/Emi 528nm
Complete
-Serum
M
sh-GFP
sh-Atg5
sh-Atg7
Atg5
Atg7
Actin
N
Atg+/+
Atg5-/-
Atg7-/-
Atg5
Atg7
Actin

Figure S3

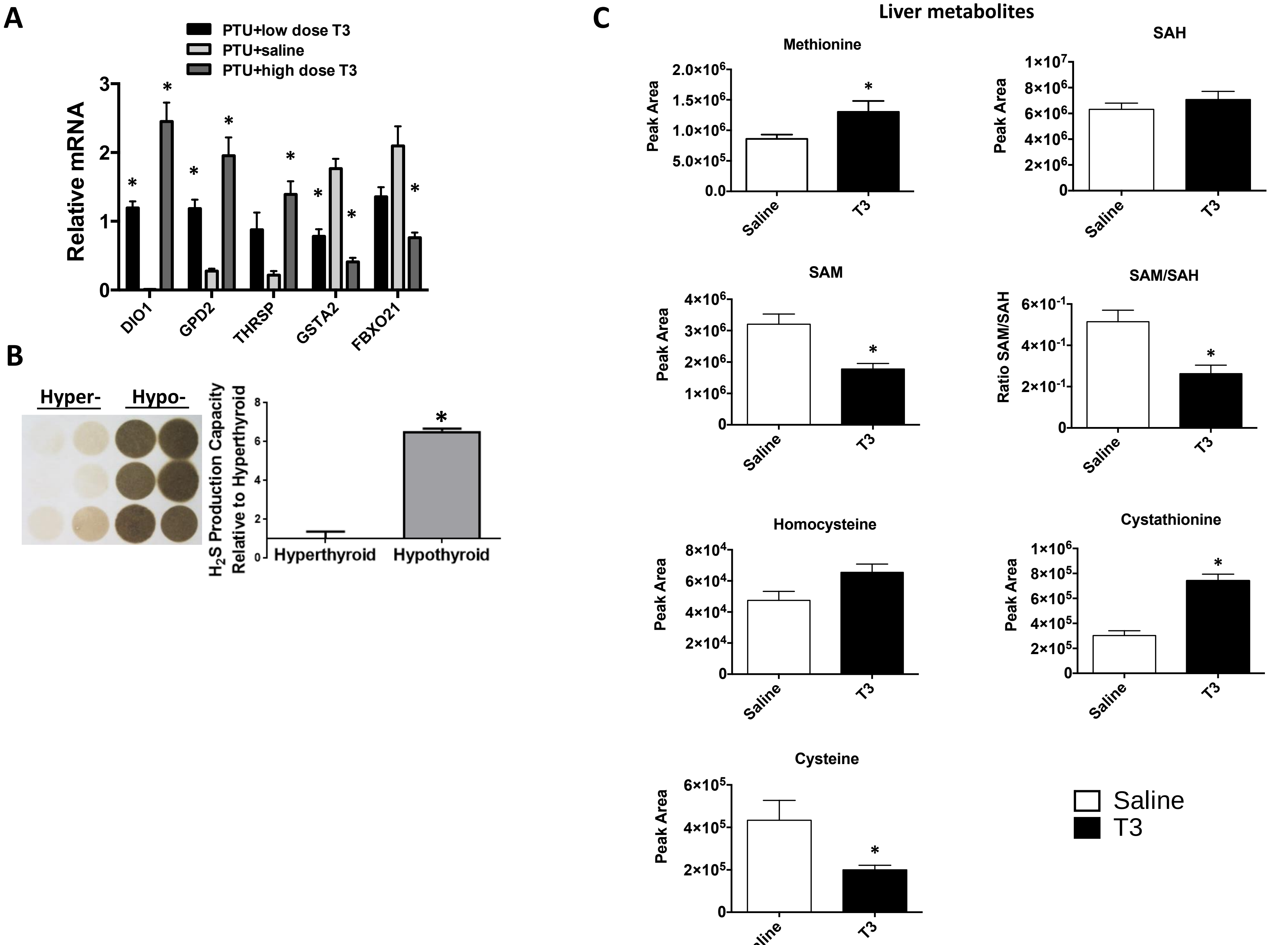

A
PTU+low dose T3
PTU+saline
PTU+high dose T3
Relative mRNA
DIO1
GPD2
THRSP
GSTA2
FBXO21
B
Hyper-
Hypo-
H2S Production Capacity Relative to Hyperthyroid
Hyperthyroid
Hypothyroid
C
Liver metabolites
Methionine
SAH
SAM
SAM/SAH
Homocysteine
Cystathionine
Cysteine
Peak Area
Ratio SAM/SAH
Saline
T3

Figure S4

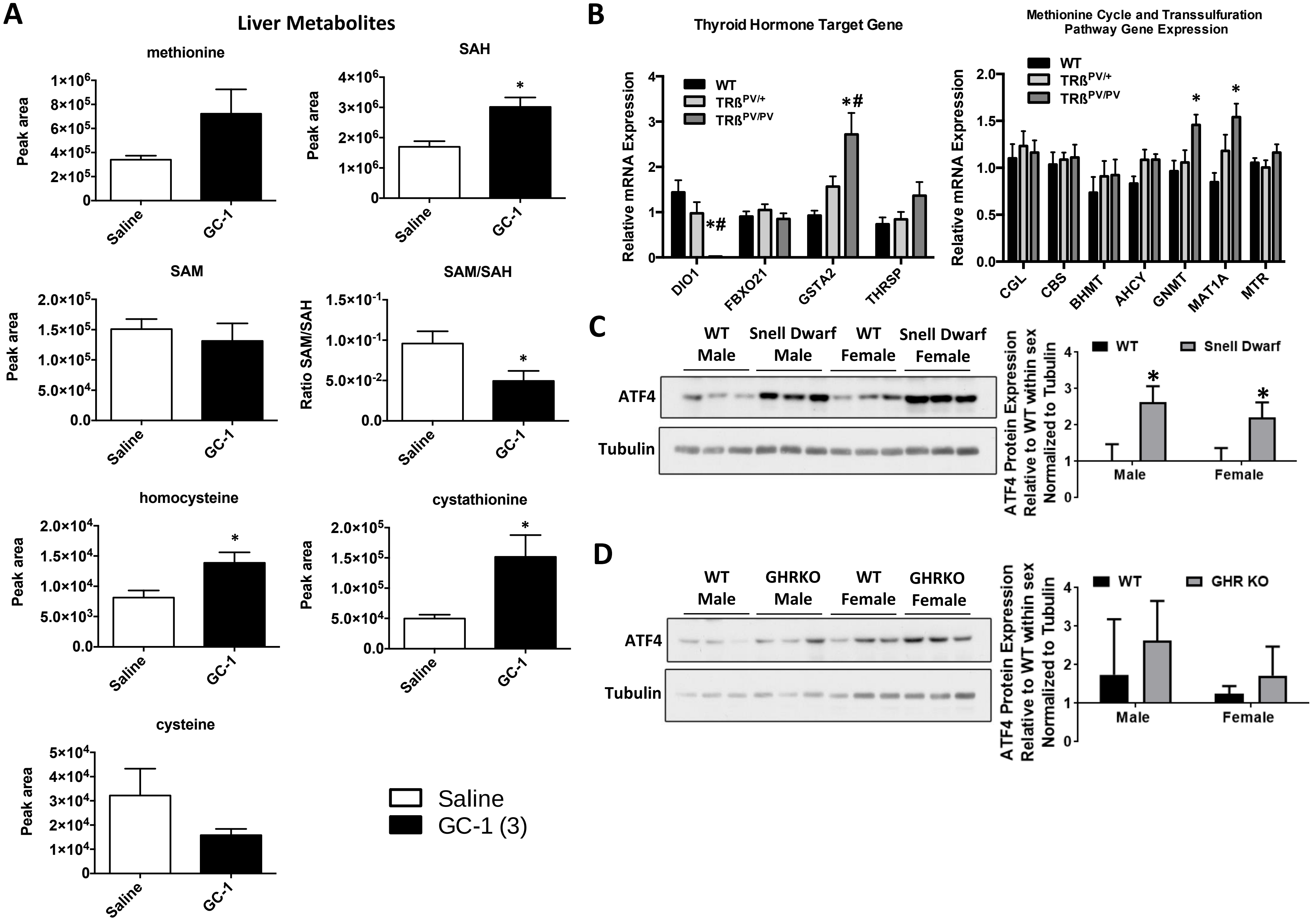
A
Liver Metabolites
methionine
SAH
SAM
SAM/SAH
homocysteine
cystathionine
cysteine
Peak area
Ratio SAM/SAH
Saline
GC-1
Saline
GC-1 (3)
B
Thyroid Hormone Target Gene
Relative mRNA Expression
WT
TRß PV/+
TRß PV/PV
DIO1
FBXO21
GSTA2
THRSP
Methionine Cycle and Transsulfuration Pathway Gene Expression
CGL
CBS
BHMT
AHCY
GNMT
MAT1A
MTR
C
WT Male
Snell Dwarf Male
WT Female
Snell Dwarf Female
ATF4
Tubulin
ATF4 Protein Expression Relative to WT within sex Normalized to Tubulin
Snell Dwarf
Male
Female
D
GHRKO Male
GHRKO Female
GHR KO

Figure S5

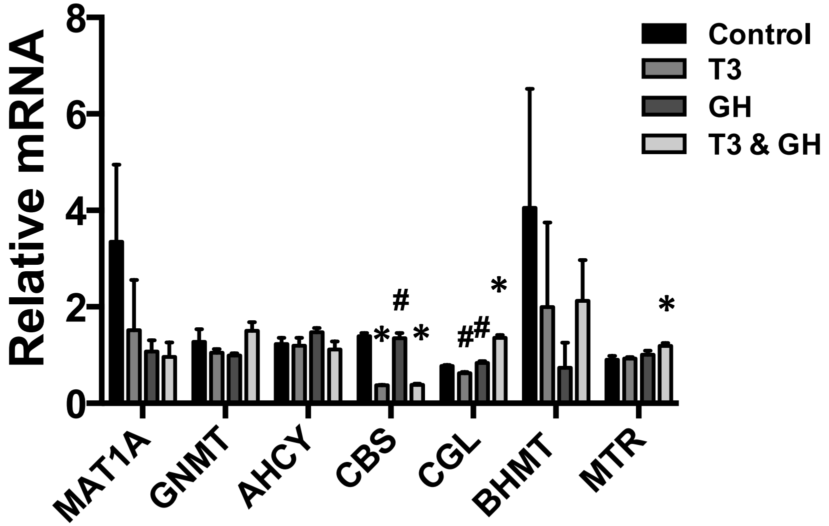

Relative mRNA
8
6
4
2
0
Control
T3
GH
T3 & GH
MAT1A
GNMT
AHCY
CBS
CGL
BHMT
MTR
#
*
*
#
#
*
*

Figure S6

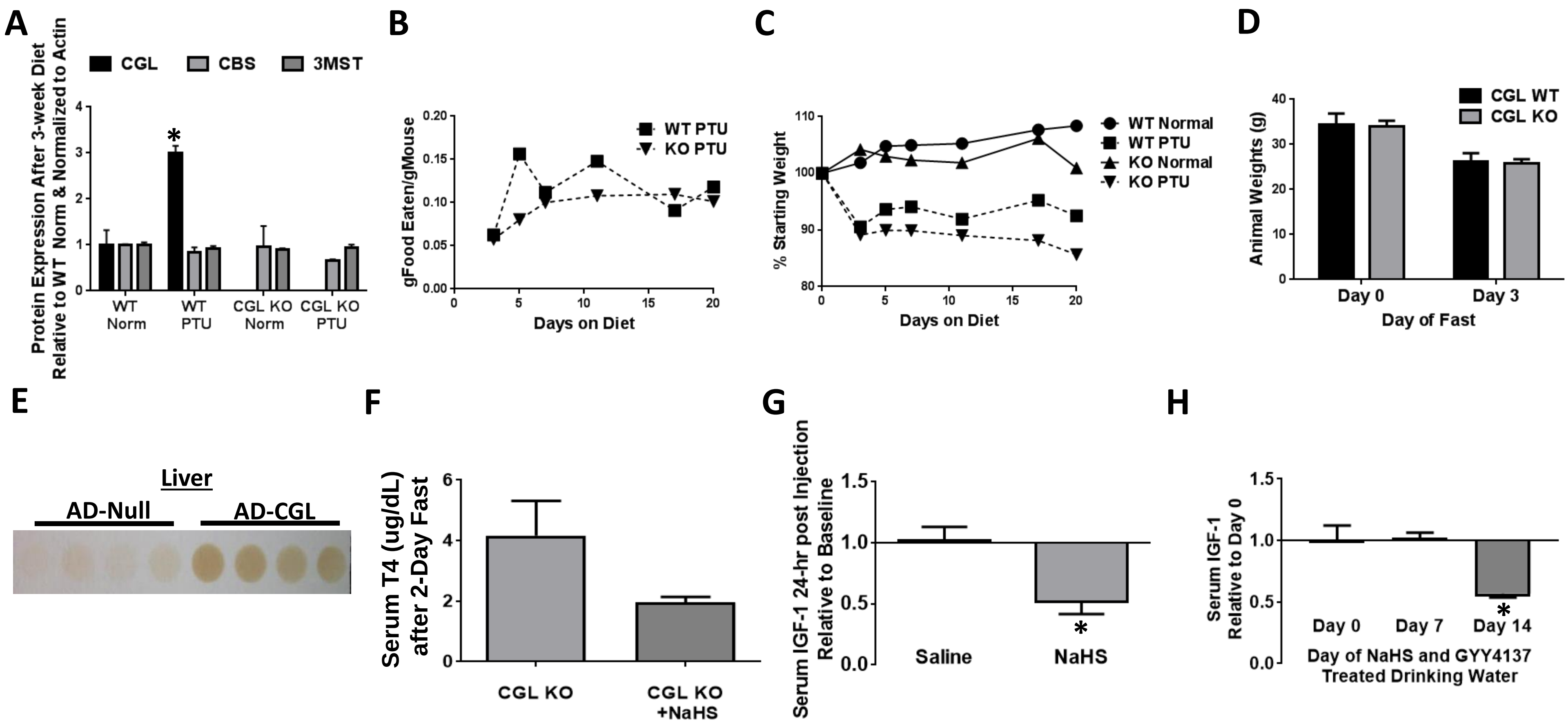

A
CGL
CBS
3MST
Protein Expression After 3-week Diet Relative to WT Norm & Normalized to Actin
WT Norm
WT PTU
CGL KO Norm
CGL KO PTU
B
WT PTU
KO PTU
gFood Eaten/gMouse
Days on Diet
C
WT Normal
WT PTU
KO Normal
KO PTU
% Starting Weight
Days on Diet
D
CGL WT
CGL KO
Animal Weights (g)
Day 0
Day 3
Day of Fast
E
Liver
AD-Null
AD-CGL
F
Serum T4 (ug/dL) after 2-Day Fast
CGL KO
CGL KO +NaHS
G
Serum IGF-1 24-hr post Injection Relative to Baseline
Saline
NaHS
H
Serum IGF-1 Relative to Day 0
Day 0
Day 7
Day 14
Day of NaHS and GYY4137 Treated Drinking Water

Figure S7

Supplemental Figure Legends:

Figure S1: Pharmacological impact on hepatic CGL and CBS expression in WT and Dwarf mice. (A) Liver CGL and CBS protein expression in18-month old Ames Dwarf and WT mice after early life saline or growth hormone (GH) injection (n=3/group). Asterisk indicates the significance of the difference between WT +Saline and Ames Dwarf +Saline and pound indicates the significance of the difference between +Saline and +GH in the Ames Dwarf groups, */#p<0.05. **(B)** Liver CGL and CBS protein expression in WT male mice after one-week of saline or lanreotide treatment (n=4/group). Asterisk indicates the significance of the difference between Saline and Lanreotide group, *p<0.05. **(C)** %Change in body mass relative to starting weight (n=4/group) and (**D**) gram of food eaten per gram of mouse body weight (n=4) during the one-week lanreotide treatment. Error bars are +/- standard error of the mean (SEM). *Related to Figure 1*

Figure S2: Growth Hormone Signaling, but Not Insulin, Represses H_2S Production Pathways *in vivo*. (A) Hepatic CGL and CBS protein expression in male mice injected for two weeks with saline, IGF-1, or GH (n=5/group). Asterisk indicates the significance of the difference between the experimental groups and the saline control group, *p<0.05. **(B)** Hepatic CGL mRNA expression in control or GH injected rats. Original source data from NCBI GeoProfile GDS862 / 8.2.2.10 / Cth (Ahluwalia, Clodfelter, & Waxman, 2004). Asterisk indicates the significance of the difference between Control and +GH, *p<0.05. **(C)** Liver CGL and CBS protein expression from male and female WT and IRS-1 KO mice (n=3/group). Asterisk indicates the significance of the difference between WT and KO within sex, *p<0.05. (**D**) Hepatic H_2S production capacity in WT or liver specific insulin receptor knockout; L-IRKO, mice (n=5). Asterisk indicates the significance of the difference between WT and L-IRKO, *p<0.05. (**E**) Analysis of H_2S producing enzymes CGL and CBS in the livers of WT or FGF21 Over Expressing; OE, mice (n=6). Asterisk indicates the significance of the difference between WT and FGF21 OE, *p<0.05. Error bars are +/- standard error of the mean (SEM). *Related to Figure 2*

Figure S3: Growth Hormone Signaling Represses H_2S Production Pathways *in vitro*. (A-B) Endogenous H_2S production in WT or CGL KO primary mouse dermal fibroblasts as measured via florescent probe and 2-photon florescent microscopy **(A)** or spectrophotometry **(B)** under different media conditions +/-PAG&AOAA (n=3). Asterisk indicates the significance of the difference between Complete and –Serum, and pound indicates the significance of the difference between CGL WT–Serum and CGL KO-Serum or the –Serum+PAG+AOAA groups, */#p<0.05. Scale bars indicate 100μm. (**C**) Measurement of endogenous H_2S production via florescent probe and spectrophotometry (n=3) in mouse primary hepatocytes under various growth conditions +/-Serum+/-GH. Asterisk indicates the significance of the difference between Complete and pound indicates the significance of the difference between –Serum and –Serum+GH; $^{*/\#}$p<0.05. (**D-E**) Western blot analysis of phospho- and total- Stat5 protein expression in primary hepatocytes grown w/ serum (**D**) and w/o serum (**E**) as a function of GH addition. (**F**) *IGF-I* mRNA expression in mouse primary hepatocytes as a function of +/-Serum and +/-GH in the media (n=3). Asterisk indicates the significance of the difference between Comp-GH, and pound indicates the significance of the difference between -Serum-GH and -Serum+GH; */#p<0.05. (**G-H**) Measurement of endogenous H_2S production via P3 florescent probe and spectrophotometry in mouse primary hepatocytes under various growth conditions: (**G**) +/-Serum +/-GH +/-AZD1480, (**H**) +/-FGF21. Asterisks indicates the significance of the difference between Complete, pound indicates the significance of the differences between -Serum, and dollar sign indicates the significance of the difference between +GH; */#/$p<0.05. (**I**) CGL and CBS mRNA expression in primary mouse hepatocytes grown in complete (Comp) media or in media lacking serum (-Serum), (n=3). (**J**) Western blot analysis of CGL and CBS expression in primary hepatocytes grown in Complete or –Serum media (n=2). (**K-L**) Measurement of endogenous H_2S production via florescent probe and spectrophotometry in mouse primary hepatocytes grown in media +/- Serum and +/- the autophagy inhibitor BAF (n=3) **(K)** or CQ (n=3) **(L)**. Asterisks indicates the significance of the difference between Complete and -Serum and pound sign indicates the significance of the difference between -Serum-BAF and -Serum+BAF **(K)**, or –Serum-CQ and –Serum+CQ **(L)**, */#<0.05. (**M-N**) Western blot analysis showing knockdown of ATG5 or ATG7 in hepa1-6 cells with lentiviral shRNA **(M)** or ATG5 and ATG7 knockout in MEF cells **(N)**. Error bars are +/- standard error of the mean (SEM). *Related to Figure 3*

Figure S4: Hypothyroidism Boosts and Thyroid Hormone Represses Hepatic H_2S Production and alters TSP Pathway *in vivo*. (**A**) Thyroid hormone receptor target gene mRNA expression (n=4) and (**B**) fresh homogenate H_2S production capacity (n=6) in livers from mice under various hypo-, hyper-, or eu-thyroid states. Asterisk indicates the significance of the difference from the hypothyroid (PTU+Saline) state; *p<0.05. **(C)** Liver metabolites related to sulfur amino acid metabolism in mice treated with T3 vs. vehicle control (saline) as indicated (n=5/group). Asterisk indicates the significance of the difference between vehicle and +T3; *p<0.05. Error bars are +/- standard error of the mean (SEM). *Related to Figure 4*

Figure S5: Hyperthyroidism Modifies the Hepatic Methionine Cycle and Transsulfuration Pathway *in vivo*. (A) Liver metabolites related to sulfur amino acid metabolism in mice treated with GC-1 vs. vehicle control (saline) as indicated (n=6-8). Asterisk indicates the significance of the difference between vehicle and +GC-1; *p<0.05. **(B)** Liver mRNA expression of thyroid hormone target related and sulfur amino acid metabolism related genes as a function of liver thyroid hormone receptor beta (TRb) status (n=4-5/group). Asterisks indicates the significance of the difference from WT and pound indicates the significance of the differences between Het ($TRb^{PV/+}$) and homo ($TRb^{PV/PV}$); $*^{/\#}$p<0.05. **(C,D)** Liver ATF4 protein expression from male and female Snell Dwarf and WT **(C**, n=3/group**)** and GHRKO and WT **(D**, n=3/group**)** mice. Asterisks indicate the significance of the difference between WT and experimental group within sex. *p<0.05. Error bars are +/- standard error of the mean (SEM). *Related to Figure 5*

Figure S6: Thyroid Hormone and Growth Hormone Effects on Hepa1 Cell Sulfur Amino Acid Metabolism Genes mRNA Expression. Analysis of mRNA expression in Hepa1 cells after overnight treatment with T3, GH, or T3 and GH (n=3). Asterisk indicates the significance of the difference from the Control group and pound indicates the significance of the difference from the T3 & GH group; $*^{/\#}$p<0.05. Error bars are +/- standard error of the mean (SEM). *Related to Figure 6*

Figure S7: CGL Status Alters the Effects of Experimental Hypothyroidism. (**A**) Quantitation of Western blot data on H_2S producing enzymes normalized to actin (n=3) in the livers of CGL WT and KO mice after 3-weeks of Normal or PTU diet. Asterisk indicates the significance of the difference between Normal and PTU diet groups within genotype; *p<0.05. (**B, C**) Food eaten per gram of mouse (n=4-5, **B**) and changes in body mass (n=4-5, **C**) in CGL WT and KO mice on Normal or PTU diets for 3-weeks. (**D**) Body mass of CGL WT and KO mice before and after a 3-day fast (n=3-4/group). **(E)** Liver H_2S production capacity from mice injected with AD-Null (control) or AD-CGL (CGL overexpression) 1-week prior (n=4/group). **(F)** Serum T4 (ug/dL) after a two day fast in CGL KO mice +/- NaHS supplementation (n=4/group). (**G-H**) Serum IGF-1 in mice treated with a single NaHS injection 24-hours prior (n=4/group , **G**) and in mice given drinking water supplemented with NaHS and GYY4137 over a two week period (n=4, **H**). Asterisks indicate the significance of the difference compared to baseline serum IGF-1 levels; *p<0.05. Error bars are +/- standard error of the mean (SEM). *Related to Figure 7*

REAGENT or RESOURCE	SOURCE	IDENTIFIER
**Oligonucleotides**		
IGF-1 F: TGCTTGCTCACCTTCACCA IGF-1 R: CAACACTCATCCACAATGCC	N/A	N/A
GHR F: ATTCACCAAGTGTCGTTCCC GHR R: TCCATTCCTGGGTCCATTCA	N/A	N/A
CGL F: TTGGATCGAAACACCCACAAA CGL R: AGCCGACTATTGAGGTCATCA	N/A	N/A
CBS F: GGGACAAGGATCGAGTCTGGA CBS R: AGCACTGTGTGATAATGTGGG	N/A	N/A
HPRT F:TTTCCCTGGTTAAGCAGTACAGCCC HPRT R:TGGCCTGTATCCAACACTTCGAGA	N/A	N/A
RPL13 F:TTCGGCTGAAGCCTACCAGAAAGT RPL13 R:TCTTCCGATAGTGCATCTTGGCCT	N/A	N/A
MAT1A F: GATAGCAGATCTGAGGCGCT MAT1A R: TGCACCATTATCCTGCATGT	N/A	N/A
GNMT F: AAGAGGGCTTCAGCGTGATG GNMT R: CTGGCAAGTGAGCAAAACTGT	N/A	N/A
AHCY F: CGCCAGCATGTCTGATAAAC AHCY R: CCTGGCATCTCATTCTCAGC	N/A	N/A
BHMT F: TTAGAACGCTTAAATGCCGGAG BHMT R: GATGAAGCTGACGAACTGCCT	N/A	N/A
Cyclophilin F: GGTGGAGAGCACCAAGACAGA Cyclophilin R: GCCGGAGTCGACAATGATG	N/A	N/A
CGL	N/A	Mm00461247_m1
CBS	N/A	Mm00460654_m1
MTR	N/A	Mm01340053_m1
DIO1	N/A	Mm00839358_m1
GPD2	N/A	Mm00439082_m1
THRSP	N/A	Mm01273967_m1
GSTA2	N/A	Mm00833353_mH
FBXO21	N/A	Mm00523921_m1

TSH alpha	N/A	Mm01209400_m1
TSH beta	N/A	Mm00437190_m1
PGC-1a	N/A	Mm01208835_m1
FGF21	N/A	Mm00840165_g1
MAT1A Promoter F: CTCCTCCACTGTCCTTGCTTG<br>MAT1A Promoter R: GGCAGATCTTTGGCAGAATCC	N/A	N/A
MAT1A intron 4 F: CACGTGCATGGGTAGAGGGAC<br>MAT1a intron 4 R: CTTTTCCACCTCCCCGAGGTC	N/A	N/A
GNMT exon 5 F: GGCTCAGGGAATGACGCAACC<br>GNMT exon 5 R: CTCTCCTGCCAAACCACGTCA	N/A	N/A
CBS intron 1 F: CCTTGTGCGGGACCCAGTTGG<br>CBS intron 1 R: GCGAATGTGGCCCAGGGTATC	N/A	N/A
CBS intron 2 F: GCACACCTGACCCTGTATCC<br>CBS intron 2 R: CTGCTGTCCTTGGTTGGACT	N/A	N/A
CGL promoter F: GGGCCTTAAGGCCTGATCTTG<br>CGL promoter R: CAGCACTGAGGTGCAGCACTC	N/A	N/A
BHMT promoter F: CGCATCCTCATGCAATGATC<br>BHMT promoter R: CTGAGGTCGCTGCTAGCTCGTC	N/A	N/A

Supplemental Table S1: List of DNA Oligos (Related to STAR Methods Key Resources Table)
