# Supplementary material for: Hypothalamic-Pituitary Axis Regulates Hydrogen Sulfide Production
Source: Cell Metab. 2017 Jun 6;25(6):1320–1333.e5. doi: 10.1016/j.cmet.2017.05.003 (PMC5722247; doi:10.1016/j.cmet.2017.05.003)
Supplement: Document S2. Article plus Supplemental Information [file mmc2.pdf]

Cell Metabolism

Article

Hypothalamic-Pituitary Axis Regulates Hydrogen Sulfide Production

Graphical Abstract

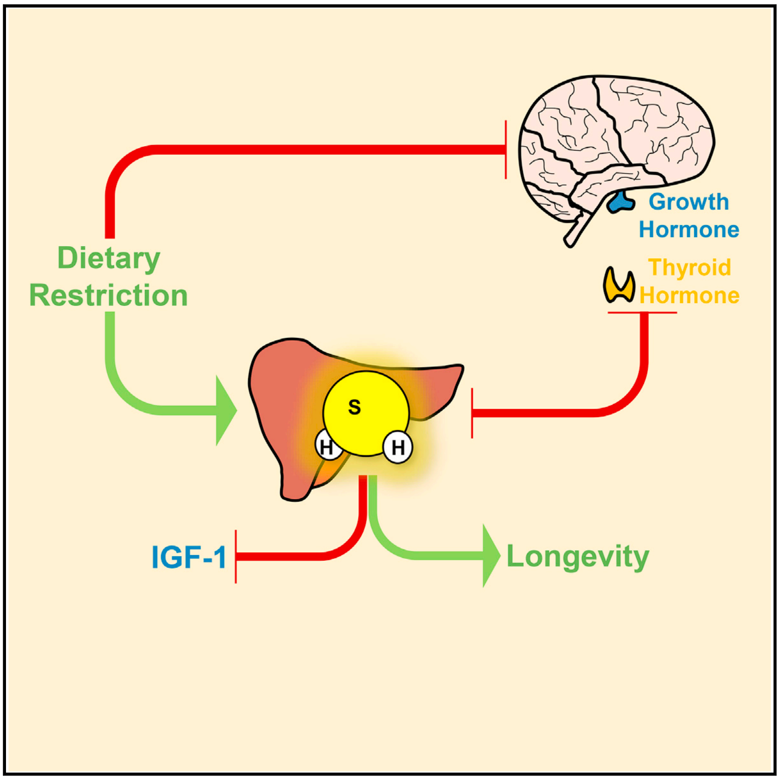

Highlights

- Hepatic H_2S production capacity is elevated in long-lived hypopituitary mouse models
- Growth hormone (GH) represses hepatic H_2S production post-transcriptionally
- Thyroid hormone (TH) acts via TRβ to repress cystathionine γ-lyase and H_2S levels
- H_2S negatively regulates circulating TH and IGF-1 levels

Authors

Christopher Hine, Hyo-Jeong Kim, Yan Zhu, ..., Richard Miller, Anthony N. Hollenberg, James R. Mitchell

Correspondence

thollenb@bidmc.harvard.edu (A.N.H.), jmitchel@hsph.harvard.edu (J.R.M.)

In Brief

Reduced thyroid hormone (TH) and growth hormone (GH) activity are hallmarks of genetic models of longevity in mice. Here, Hine et al. find that TH and GH negatively regulate hepatic production of the longevity-associated gas hydrogen sulfide, which feeds back to negatively regulate circulating TH and IGF-1 levels.

Hine et al., 2017, Cell Metabolism *25*, 1320–1333
June 6, 2017 © 2017 The Authors. Published by Elsevier Inc.
http://dx.doi.org/10.1016/j.cmet.2017.05.003

CellPress

Hypothalamic-Pituitary Axis Regulates Hydrogen Sulfide Production

Christopher Hine,[1,15,16] Hyo-Jeong Kim,[2,15] Yan Zhu,[2,15] Eylul Harputlugil,[1] Alban Longchamp,[1,4] Marina Souza Matos,[2] Preeti Ramadoss,[2] Kevin Bauerle,[2] Lear Brace,[1] John M. Asara,[3] C. Keith Ozaki,[4] Sheue-yann Cheng,[5] Subhankar Singha,[6] Kyo Han Ahn,[6] Alec Kimmelman,[7] Ffolliott M. Fisher,[2] Pavlos Pissios,[2] Dominic J. Withers,[8] Colin Selman,[9] Rui Wang,[10] Kelvin Yen,[11] Valter D. Longo,[11] Pinchas Cohen,[11] Andrzej Bartke,[12] John J. Kopchick,[13] Richard Miller,[14] Anthony N. Hollenberg,[2,*] and James R. Mitchell[1,17,*]
[1]Department of Genetics and Complex Diseases, Harvard T.H. Chan School of Public Health, Boston, MA 02115, USA
[2]Division of Endocrinology, Diabetes and Metabolism
[3]Division of Signal Transduction, Department of Medicine
Beth Israel Deaconess Medical Center, Harvard Medical School, Boston, MA 02215, USA
[4]Department of Surgery, Heart and Vascular Center Brigham and Women's Hospital, Harvard Medical School, Boston, MA 02115, USA
[5]Laboratory of Molecular Biology, Center for Cancer Research, National Cancer Institute, National Institutes of Health, Bethesda, MD 20892, USA
[6]Department of Chemistry, Center for Electro-Photo Behaviors in Advanced Molecular Systems, POSTECH, 77 Cheongam-Ro, Nam-Gu, Pohang 790-784, Republic of Korea
[7]Department of Radiation Oncology, Dana-Farber Cancer Institute, Boston, MA 02215, USA
[8]Medical Research Council Clinical Science Centre, Imperial College, London W12 0NN, UK
[9]Glasgow Ageing Research Network, Institute of Biodiversity, Animal Health and Comparative Medicine, College of Medical, Veterinary and Life Sciences, University of Glasgow, Glasgow G12 8QQ, UK
[10]Department of Biology, Lakehead University, Thunder Bay, ON P7B 5E1, Canada
[11]Department of Biological Sciences, Longevity Institute, School of Gerontology, University of Southern California, Los Angeles, CA 90089, USA
[12]Department of Internal Medicine, Southern Illinois University School of Medicine, Springfield, IL 62794, USA
[13]Edison Biotechnology Institute, Heritage College of Osteopathic Medicine, Ohio University, Athens, OH 45701, USA
[14]Department of Pathology & Geriatrics Center, University of Michigan, Ann Arbor, MI 48109, USA
[15]These authors contributed equally
[16]Present address: Department of Cellular and Molecular Medicine, Cleveland Clinic, Cleveland, OH 44195, USA
[17]Lead Contact
*Correspondence: thollenb@bidmc.harvard.edu (A.N.H.), jmitchel@hsph.harvard.edu (J.R.M.)
http://dx.doi.org/10.1016/j.cmet.2017.05.003

SUMMARY

Decreased growth hormone (GH) and thyroid hormone (TH) signaling are associated with longevity and metabolic fitness. The mechanisms underlying these benefits are poorly understood, but may overlap with those of dietary restriction (DR), which imparts similar benefits. Recently we discovered that hydrogen sulfide (H_2S) is increased upon DR and plays an essential role in mediating DR benefits across evolutionary boundaries. Here we found increased hepatic H_2S production in long-lived mouse strains of reduced GH and/or TH action, and in a cell-autonomous manner upon serum withdrawal in vitro. Negative regulation of hepatic H_2S production by GH and TH was additive and occurred via distinct mechanisms, namely direct transcriptional repression of the H_2S-producing enzyme cystathionine γ-lyase (CGL) by TH, and substrate-level control of H_2S production by GH. Mice lacking CGL failed to downregulate systemic T_4 metabolism and circulating IGF-1, revealing an essential role for H_2S in the regulation of key longevity-associated hormones.

INTRODUCTION

Hydrogen sulfide (H_2S) affects numerous aspects of animal physiology (Wang, 2012), including long-term potentiation in the nervous system (Abe and Kimura, 1996), vasorelaxation (Zhao et al., 2001), oxygen sensing (Olson et al., 2006), angiogenesis in the cardiovascular system (Cai et al., 2007; Papapetropoulos et al., 2009), and insulin secretion from endocrine cells in the pancreas (Yang et al., 2005). While H_2S at high concentrations is toxic, low levels impart numerous benefits including resistance to hypoxia (Blackstone and Roth, 2007), neuroprotection (Kimura and Kimura, 2004), protection from myocardial ischemia reperfusion injury (Bian et al., 2006; Elrod et al., 2007), modulation of inflammation (Zanardo et al., 2006), and extension of longevity (Miller and Roth, 2007). H_2S is also produced endogenously by several enzymes, including 3-MST and the transsulfuration pathway (TSP) enzymes cystathionine β-lyase (CBS) and cystathionine γ-lyase (CGL) (Kabil et al., 2011). Mice lacking functional TSP activity are hypertensive (Yang et al., 2008), display reduced angiogenic potential (Szabó and Papapetropoulos, 2011), and are susceptible to aging-related neurodegeneration and osteoporosis (Liu et al., 2014; Paul et al., 2014). Despite the implications for its pleiotropic beneficial

 © 2017 The Authors. Published by Elsevier Inc.
This is an open access article under the CC BY license (http://creativecommons.org/licenses/by/4.0/).

effects, little is known about the systemic regulation of endogenous H_2S production.

Previously, we reported that dietary restriction (DR), best known for increasing lifespan, stress resistance, and metabolic fitness in organisms across evolutionary boundaries, works partially through increasing endogenous H_2S production (Hine et al., 2015). In rodents, DR-mediated protection from hepatic ischemia reperfusion injury requires H_2S generation by CGL, which is subject to regulation by sulfur amino acid intake (Hine et al., 2015; Nakano et al., 2015; Sikalidis and Stipanuk, 2010). Downstream mechanisms of H_2S action in this context, and whether these are specific to DR or shared with other anti-aging interventions, remain unknown.

Like DR, reduced hypothalamic-pituitary axis activity is associated with resistance to age-related diseases, extended longevity, and improved metabolic fitness in rodents and humans. Long-lived rodent strains include hypopituitary Snell and Ames dwarf mice that lack growth hormone (GH) and thyroid-stimulating hormone (TSH) (Bartke and Brown-Borg, 2004). Specific ablation of the GH receptor (GHR) in GHR knockout mice (GHRKO) also increases lifespan, suggesting the specificity of the GH pathway in aging (Coschigano et al., 2003). However, TH levels are also reduced in GHRKO mice, complicating the functional dissection of GH and TH activity in longevity control in vivo (Hauck et al., 2001). Subclinical hypothyroidism is associated with longevity in human centenarian studies (Atzmon et al., 2009; Rozing et al., 2010), and inactivating mutations in GHR are associated with metabolic fitness and reduced cancer incidence in humans (Guevara-Aguirre et al., 2011).

GHRKO mice are recalcitrant to further increase in lifespan or insulin sensitivity upon DR, consistent with at least partially overlapping mechanisms of action (Bonkowski et al., 2006). One such candidate mechanism is the insulin-like growth factor-1 (IGF-1) pathway, which is reduced upon DR as well as in hypopituitary/GHRKO longevity models. Here, we tested the hypothesis that increased H_2S production is a shared phenotype in genetic models of longevity involving decreased GH/TH signaling with the potential to contribute to metabolic benefits. We found that GH and TH negatively regulate hepatic H_2S production through distinct mechanisms, with functional consequences on feedback control of hepatic IGF-1 and TH production.

RESULTS

Increased Hepatic H_2S Production in Long-Lived Hypopituitary Dwarf Mice In Vivo

We examined the impact of reduced GH and TSH signaling on hepatic CGL and CBS mRNA and protein expression and H_2S production capacity in Snell dwarf mice lacking these hormones (Figures 1A–1D). Male and female Snell dwarf mice had increases in hepatic CGL mRNA (Figure 1A) and protein (upper band) compared with wild-type (WT) littermates (Figure 1B). Hepatic CBS mRNA was not increased in either male or female mice (Figure 1A), but CBS protein levels were increased in male mice compared with WT littermates (Figure 1B). Consistent with enzyme levels, H_2S production capacity, as measured by the lead sulfide method, was increased in liver homogenates in both male and female Snell dwarf mice (Figure 1C). Importantly, endogenous H_2S levels, as detected by two-photon microscopy using the H_2S-specific chemo-fluorescent probe P3 (Singha et al., 2015), were also increased in Snell dwarf livers (Figure 1D).

To test if increased H_2S production could have functional consequences on hypopituitary dwarf mice, we made use of the fact that GH treatment of Ames dwarf mice during early post-natal development (weeks 2-8) reverses lifespan and metabolic effects (Panici et al., 2010). Early GH treatment normalized (reduced) hepatic H_2S production capacity (Figure 1E) and CGL protein levels (Figure S1A) measured later in life at 18 months of age. These data demonstrate a correlation between hepatic H_2S production capacity and longevity in the Ames dwarf model.

Because Snell and Ames dwarf mice lack GH and TH signals from birth, we next tested the plasticity of H_2S regulation by GH/TH in WT adult mice by pharmacological inhibition with the somatostatin analog, lanreotide (Kuhn et al., 1994). Treatment for 8 days increased liver H_2S production capacity (Figure 1F) and CGL protein levels (Figure S1B) without affecting body weight (Figure S1C) or food intake (Figure S1D). These data demonstrate that GH and TSH deficiency/inhibition promote hepatic H_2S production in vivo.

GH Signaling Inhibits Hepatic H_2S Production In Vivo

We next focused specifically on the potential of GH to regulate H_2S production using global GHRKO mice. Male and female GHRKO mice had increases in hepatic CGL mRNA and protein relative to WT littermates (Figures 2A and 2B). As in Snell dwarf mice, CBS protein was increased in GHRKO males, while CBS mRNA expression was unchanged (Figures 2A and 2B). Hepatic H_2S production capacity was increased in both male and female GHRKO mice compared with WT littermates (Figure 2C). Thus, the lack of GH signaling due to global deletion of its receptor results in increased hepatic H_2S production capacity in vivo.

As IGF-1 is a major downstream effector of hepatic GH signaling, we tested the effects of 2 weeks of recombinant human IGF-1 or human GH injections on hepatic H_2S production capacity and CGL protein expression. While IGF-1 had no effect relative to mock treatment, GH injection reduced hepatic H_2S production capacity (Figure 2D) and hepatic CGL protein expression (Figure S2A). Consistent with GH-mediated repression of H_2S production capacity, GH injection suppresses CGL mRNA expression according to independent data obtained from NCBI GeoProfile GDS862/8.2.2.10/Cth (Ahluwalia et al., 2004) (Figure S2B). Together, these data indicate that GH and GHR signaling suppress hepatic H_2S production capacity in vivo independent of IGF-1.

To confirm the specific role of GHR signaling in H_2S regulation, we tested the potential of two intracellular mediators of hepatic GHR signaling, IRS-1 and FGF21, to alter H_2S production. IRS-1 is an adaptor protein involved in both insulin and GHR signaling (Liang et al., 1999), and global IRS-1KO mice are long lived (Selman et al., 2011). IRS-1KO mice had increased hepatic H_2S production capacity (Figure 2E) and elevated CGL protein expression (Figure S2C). Because IRS-1KO mice are insulin resistant (Biddinger et al., 2008),

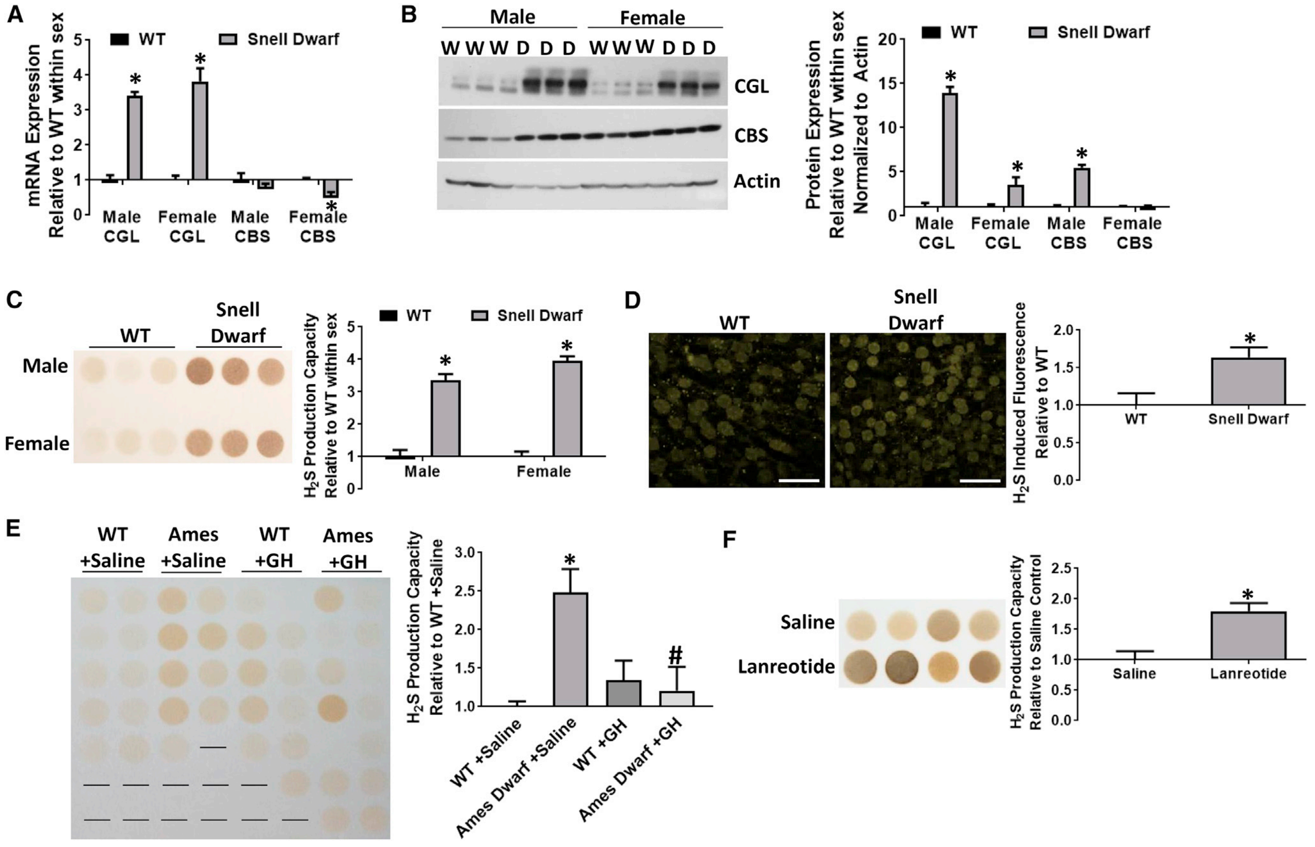

Figure 1. Increased Hepatic H_2S Production in Long-Lived Hypopituitary Dwarf Mice In Vivo

(A–D) Hepatic CGL and CBS mRNA expression (n = 3/group) (A), protein expression (n = 3/group) (B), H_2S production capacity via the lead sulfide method (n = 3/group) (C), and endogenous H_2S production via two-photon florescence microscopy (n = 3/group) (D) in male and female WT or Snell dwarf mice as indicated. Scale bar, 25 μm. Asterisk indicates the significance of the difference between genotypes within sex; $^*p < 0.05$.

(E) Liver H_2S production capacity in 18-month-old female Ames dwarf or WT mice treated +/− growth hormone during postnatal development at weeks 2-8 (n = 9–14/group). The asterisk indicates the significance of the difference between the WT+Saline control group and experimental group, and the # sign indicates the significance of the difference between +Saline and +GH; $^{*/\#}p < 0.05$.

(F) Liver H_2S production capacity in mice treated with saline control or lanreotide (n = 4/group). The asterisks indicate the significance of the difference between treatment groups; $^*p < 0.05$. Error bars are ± SEM. See also Figure S1.

we tested the potential contribution of insulin receptor (IR) signaling using liver-specific insulin receptor knockout (LIRKO mice). However, LIRKO mice displayed decreased hepatic H_2S production capacity (Figure S2D), consistent with GH signaling rather than IR signaling in negative regulation of hepatic H_2S production. In addition, long-lived mice overexpressing the fasting hormone FGF21 (Zhang et al., 2012), an intracellular inhibitor of GH signaling (Inagaki et al., 2008), displayed increased hepatic H_2S production capacity (Figure 2F) and CGL protein (Figure 2E). These data are consistent with GH/GHR as a negative regulator of H_2S production capacity, and show a positive correlation between increased hepatic H_2S production capacity and extended longevity in vivo.

GH/GHR Signaling Inhibits Hepatic H_2S Production In Vitro

To elucidate how GHR signaling controls H_2S production, we turned to overnight serum withdrawal with or without added recombinant GH in cell culture as a cell-autonomous model. Endogenous H_2S production was measured using the fluorescent P3 probe and visualized/quantitated by two-photon microscopy or UV spectrophotometry. We first established the ability of overnight serum withdrawal to induce robust endogenous H_2S production, and the specific contribution of TSP enzymes CGL and CBS to this process using WT versus CGLKO fibroblasts with or without PAG and AOAA, inhibitors of CGL and CBS, respectively (Figures S3A and S3B). Interestingly, while CGL is the predominant H_2S producer in liver in vivo (Kabil et al., 2011), and responsible for the increase in H_2S production capacity in response to DR (Hine et al., 2015), CBS also contributed to the increase in H_2S production upon serum withdrawal in primary fibroblasts in vitro (Figure S3A).

In cultured mouse primary hepatocytes, H_2S production was also significantly increased upon serum deprivation (Figures 3A and S3C). Importantly, addition of recombinant GH at the level required to induce robust phosphorylation of Stat5 (p-Stat5), and transcription of *Igf-1* (Figure S3D–S3F), dampened the increase in H_2S production induced by serum deprivation (Figures 3A and S3C).

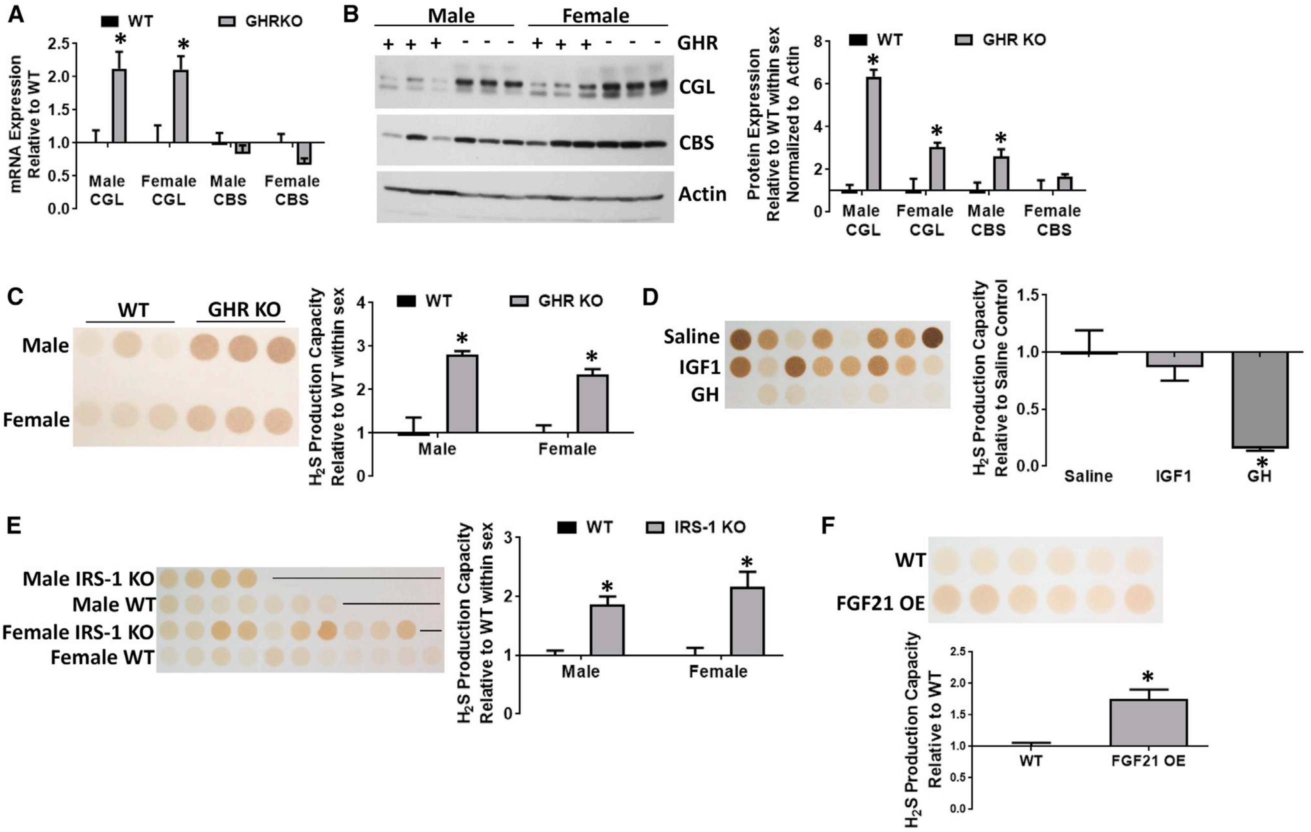

Figure 2. Growth Hormone Signaling Inhibits Hepatic H_2S Production In vivo

(A–C) Hepatic CGL and CBS mRNA expression (n = 3/group) (A), protein expression (n = 3/group) (B), and H_2S production capacity (n = 3/group) (C) in male and female growth hormone receptor knockout (GHRKO) mice. The asterisk indicates the significance of the difference between genotypes within sex; $^*p < 0.05$.

(D) Liver H_2S production capacity (n = 8/group) in WT mice treated for 2 weeks with recombinant IGF-1, GH, or saline vehicle as indicated. The asterisk indicates the significance of the difference between GH and saline treatment; $^*p < 0.05$.

(E and F) Liver H_2S production capacity in male and female IRS-1 WT or KO mice (n = 4–11/group) (E) and in male FGF21 WT or overexpressing (OE) mice (n = 6/group) (F). The asterisk indicates the significance of the difference between genotypes within sex; $^*p < 0.05$. Error bars are ± SEM. See also Figure S2.

Canonical GH-induced intracellular signaling begins with its binding to and dimerization of the GHR on the plasma membrane, leading to stimulation of numerous signaling cascades, including Jak2/Stat5, followed by the transcriptional/translational regulation of target genes such as *Igf-1*. While addition of GH into medium containing serum did not change H_2S production, blocking GH signaling with the Jak2 inhibitor AZD1480 (Gu et al., 2013) moderately increased endogenous H_2S in hepatocytes cultured in Complete medium with additional GH (Figures 3B and S3G). Furthermore, the increase in H_2S production induced by serum deprivation and blocked by GH was fully restored with addition of AZD1480 (Figures 3B and S3G). Exogenous FGF21 increased H_2S production despite the presence of full serum (Figures 3C and S3H). Together, these data suggest that GH acts through the Jak2/Stat5 pathway to repress H_2S production in cells.

Because of increased TSP mRNA/protein expression in Snell/Ames and GHRKO livers in vivo, we next asked if GH regulates hepatic H_2S production in a cell-autonomous manner via transcriptional control of TSP gene expression, in a manner similar to control of Igf-1 (Figure 3D). Surprisingly, serum withdrawal from primary hepatocytes failed to increase CGL mRNA or protein to the same levels as observed in vivo (Figures S3I and S3J). Similarly, pharmacological inhibition of GH/GHR action with AZD1480 or FGF21 failed to significantly affect TSP gene expression as they did IGF-1 expression (Figure 3D). Together, these data suggest that neither transcriptional nor translational control of CBS or CGL are the major mechanisms of hepatic H_2S regulation by GH in cells.

Regulation of endogenous H_2S production via CBS and CGL in cells could instead occur post-translationally or be driven by substrate availability (Kabil et al., 2011; Majtan et al., 2014; Zhao et al., 2014). While the endogenous source of free cysteine for H_2S production is currently unknown, cellular autophagy is increased in long-lived dwarf mice (Wang and Miller, 2012), thus potentially fueling the increase in H_2S production observed upon GH signaling inhibition. Consistent with this notion, pharmacological inhibition of autophagy with bafilomycin (BAF) or chloroquine (CQ) blocked H_2S production induced by serum removal (Figures 3E, 3F, S3K, and S3L), even in the presence of media lacking sulfur amino acids (SAA) (Figure 3F). Similarly, inhibition of autophagy by genetic knockdown (Figures 3G, S3M) or knockout (Figure 3H and S3N) of Autophagy Protein 5 (ATG5) or 7

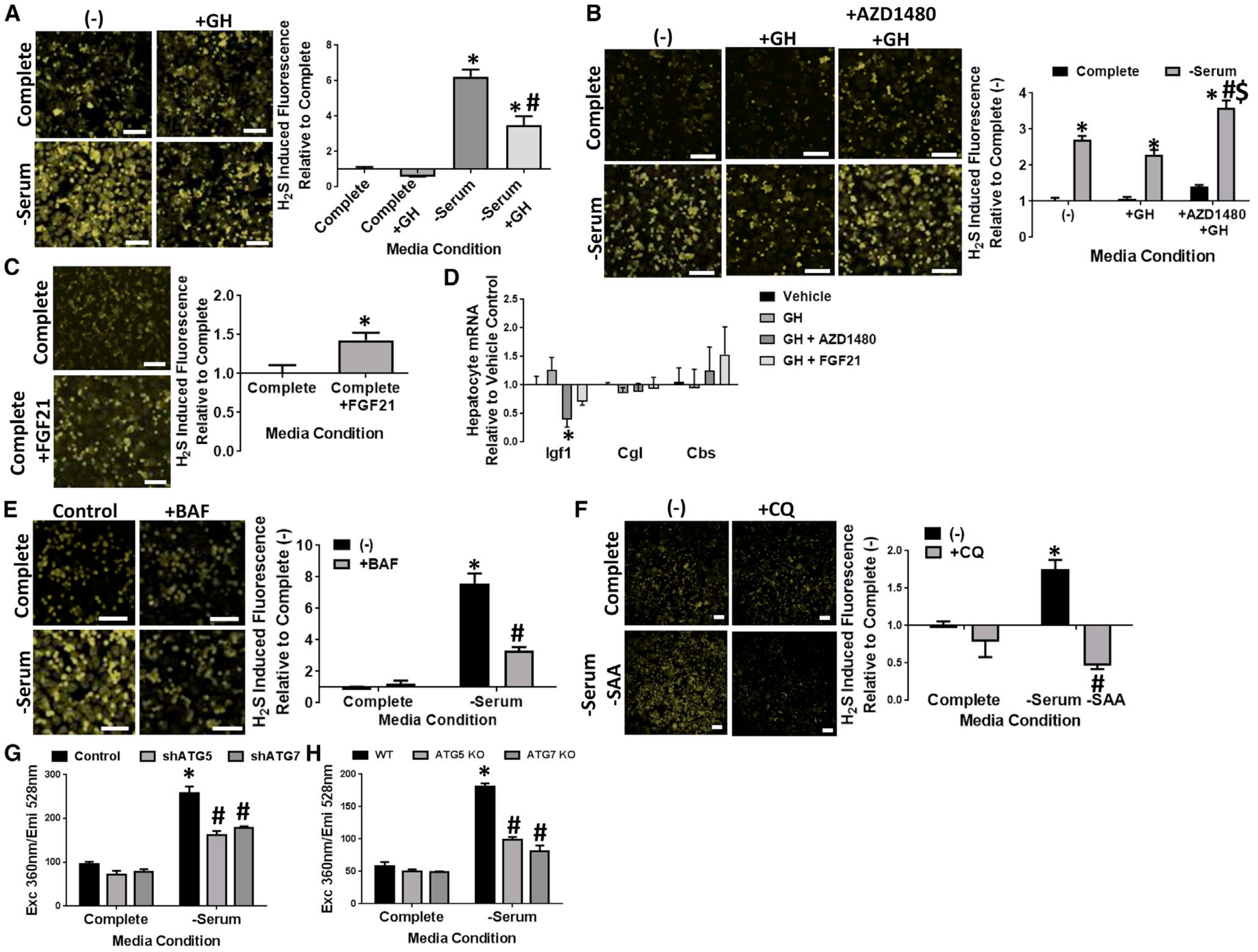

Figure 3. Growth Hormone Receptor Signaling Inhibits Hepatic H_2S Production In Vitro

(A–C) Endogenous H_2S production in primary mouse hepatocytes as measured via two-photon fluorescent microscopy under different medium conditions: (A) +/−growth serum and +/−GH, with the asterisk indicating the significance of the difference between Complete and −Serum, and the # sign indicating the significance of the difference between −Serum and −Serum+GH, $^{*/\#}p < 0.05$; (B) +/−growth serum, +/−GH, +/−AZD1480, with the asterisk indicating the significance of the difference between Complete and −Serum in each group, and the # sign indicating the significance of the difference between −Serum (−) (no addition) and −Serum+ZD1480; and the $ sign indicating the significance of the difference between +GH and +GH+ZD1480 for both Complete and −Serum conditions, $^{*/\#/\$}p < 0.05$; (C) +/−FGF21 in Complete medium containing serum, with the asterisk indicating the significance of the difference between Complete and Complete+FGF21; $^{*}p < 0.05$.

(D) mRNA expression of *Igf-I*, Cgl, and Cbs in mouse primary hepatocytes. The asterisk indicates the significance of the difference between +GH and +GH+ZD1480; $^{*}p < 0.05$.

(E and F) Endogenous H_2S production in mouse primary hepatocytes by florescent microscopy after overnight treatment under the indicated medium conditions followed by addition of the P3 probe for 1 hr. The asterisk indicates the significance of the difference between Complete and −Serum, and the # sign indicates the significance of the difference between −Serum and −Serum+BAF (E), or −Serum−SAA and −Serum−SAA+CQ (F); $^{*\#}p < 0.05$.

(G and H) Endogenous H_2S production in Hepa1-6 cells with or without knockdown of autophagy components ATG5 and ATG7 by shRNA (G) or in MEFs by genetic knockout of ATG5 and ATG7 (H) detected by UV spectrophotometry after overnight treatment with or without serum followed by addition of the P3 probe for 1 hr. The asterisk indicates the significance of the difference between Complete and −Serum in the Control group, and the # sign indicates the significance of the difference between −Serum Control and −Serum ATG5 deficient or −Serum ATG7 deficient; $^{*/\#}p < 0.05$. Each experiment was repeated at least three times. Scale bars, 100 μm (A–C, E, and F). Error bars are ± SEM. See also Figure S3.

(ATG7) decreased H_2S production upon serum withdrawal. Taken together, these data suggest that GH is a negative regulator of H_2S production in vitro through autophagy-dependent substrate-level and/or enzymatic activity control rather than transcriptional control of CGL and CBS expression.

Hypothyroidism Increases Hepatic H_2S Production In Vivo

We next considered the potential of TH to explain the transcriptional control of hepatic CGL expression observed in these models in vivo but not readily attributable to GHR activity in vitro. Adult male mice were made hypo-, eu-, or hyperthyroid

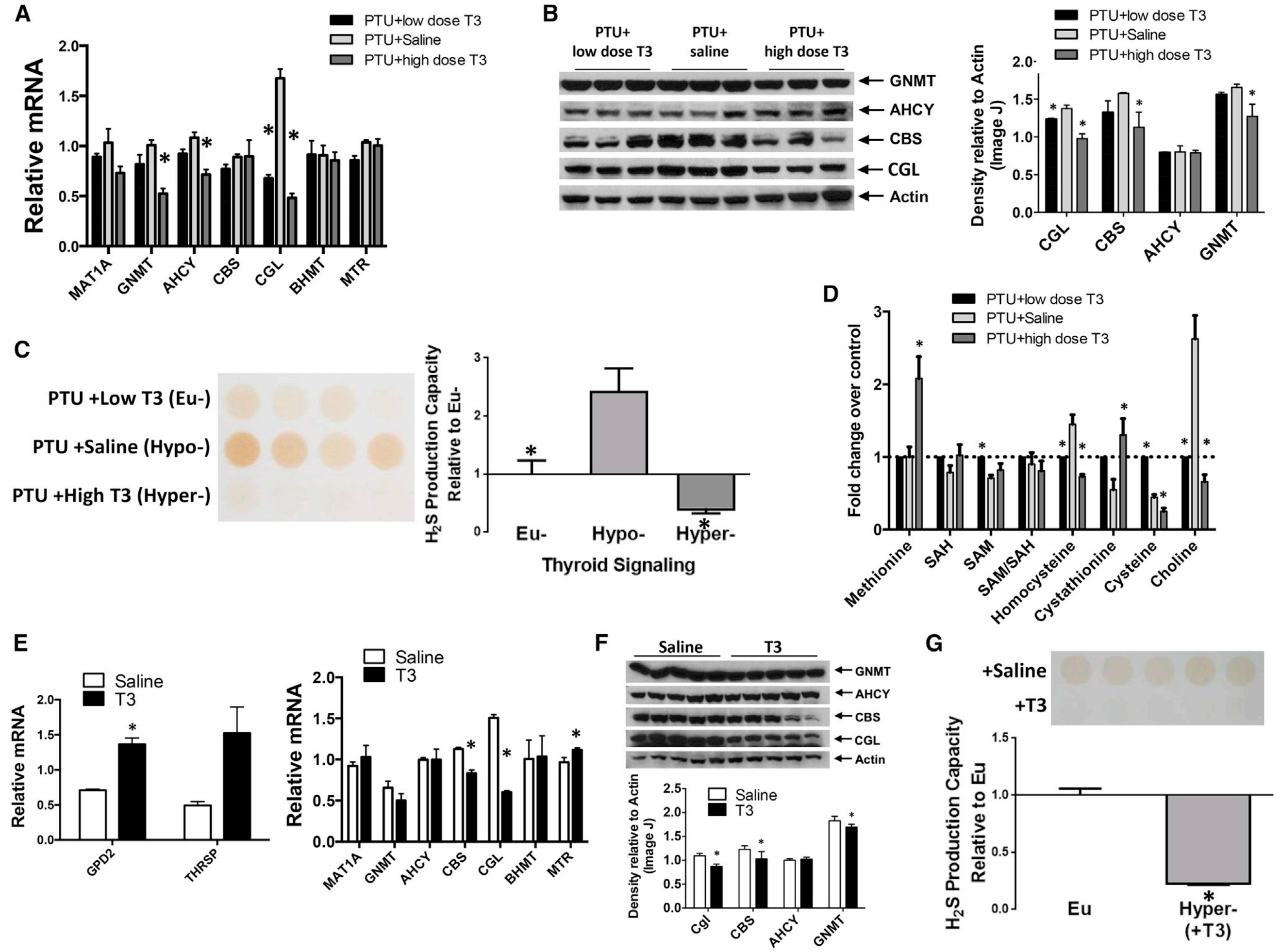

Figure 4. Hypothyroidism Increases and Thyroid Hormone Represses Hepatic H_2S Production In Vivo

(A–D) mRNA expression (n = 4) (A), protein expression (n = 3) (B), H_2S production capacity (n = 4) (C), and transmethylation/transsulfuration metabolite levels by liquid chromatography-tandem mass spectrometry (n = 4) (D) in livers of mice under hypo- (PTU+Saline), hyper- (PTU+high dose T3), and eu-(PTU+low dose T3) thyroid states. The asterisks indicate the significance of the difference from the hypothyroid state (PTU+Saline); $^*p < 0.05$.

(E–G) Analysis of TH-responsive and sulfur amino acid metabolism-associated mRNA levels (n = 4–6) (E), protein expression (n = 5) (F), and H_2S production capacity (n = 5) (G) in livers of mice treated with T3 (hyperthyroid) versus vehicle (saline) control (euthyroid). The asterisk indicates the significance of the difference between vehicle and +T3 groups; $^*p < 0.05$. Error bars are ± SEM. See also Figure S4.

by inhibiting endogenous thyroid function with a PTU-containing low iodine diet (PTU/LID) with or without TH supplementation (in the form of T_3). To validate the system, we confirmed that known TH target genes were regulated as expected in the hypothyroid (PTU/LID), euthyroid (PTU/LID + low dose T3), and hyperthyroid (PTU/LID + high dose T_3) states (Figure S4A).

Circulating T_3 levels strongly correlated with expression of genes in the methionine cycle and TSP including CGL, which was activated in the hypothyroid state and strongly repressed by increasing doses of T_3 (Figure 4A). A similar correlation was observed between T_3 and hepatic protein levels of CGL and CBS (Figure 4B) as well as hepatic H_2S production capacity (Figures 4C and S4B). Metabolomics revealed broader effects of circulating T_3 on hepatic methionine cycle and TSP metabolites, including regulation of homocysteine, cystathionine, and cysteine (Figure 4D). Finally, we confirmed that T_3 alone, without the induction of hypothyroidism via a PTU/LID, reduced hepatic CGL and CBS mRNA (Figure 4E), correlating with protein levels (Figure 4F), hepatic H_2S production capacity (Figure 4G) and characteristic changes in hepatic metabolites, including cystathionine and cysteine (Figure S4C).

Thyroid Hormone Signaling through TRβ Suppresses Hepatic H_2S Production

While T_3 acts broadly in vivo through multiple TH receptors, its action in liver depends primarily on TH receptor β1 (TRβ1). To test if negative regulation of TSP expression and H_2S production capacity by T_3 is organ autonomous or the result of systemic T_3 action, we took advantage of the T_3 analog GC-1, which preferentially acts on the liver via its uptake and specificity for TRβ1. GC-1 predictably modulated known T_3 target genes in the liver (Figure 5A, left) and negatively regulated components of the hepatic methionine cycle and TSP, including CGL and CBS gene and protein expression (Figures 5A and 5B). GC-1 also reduced

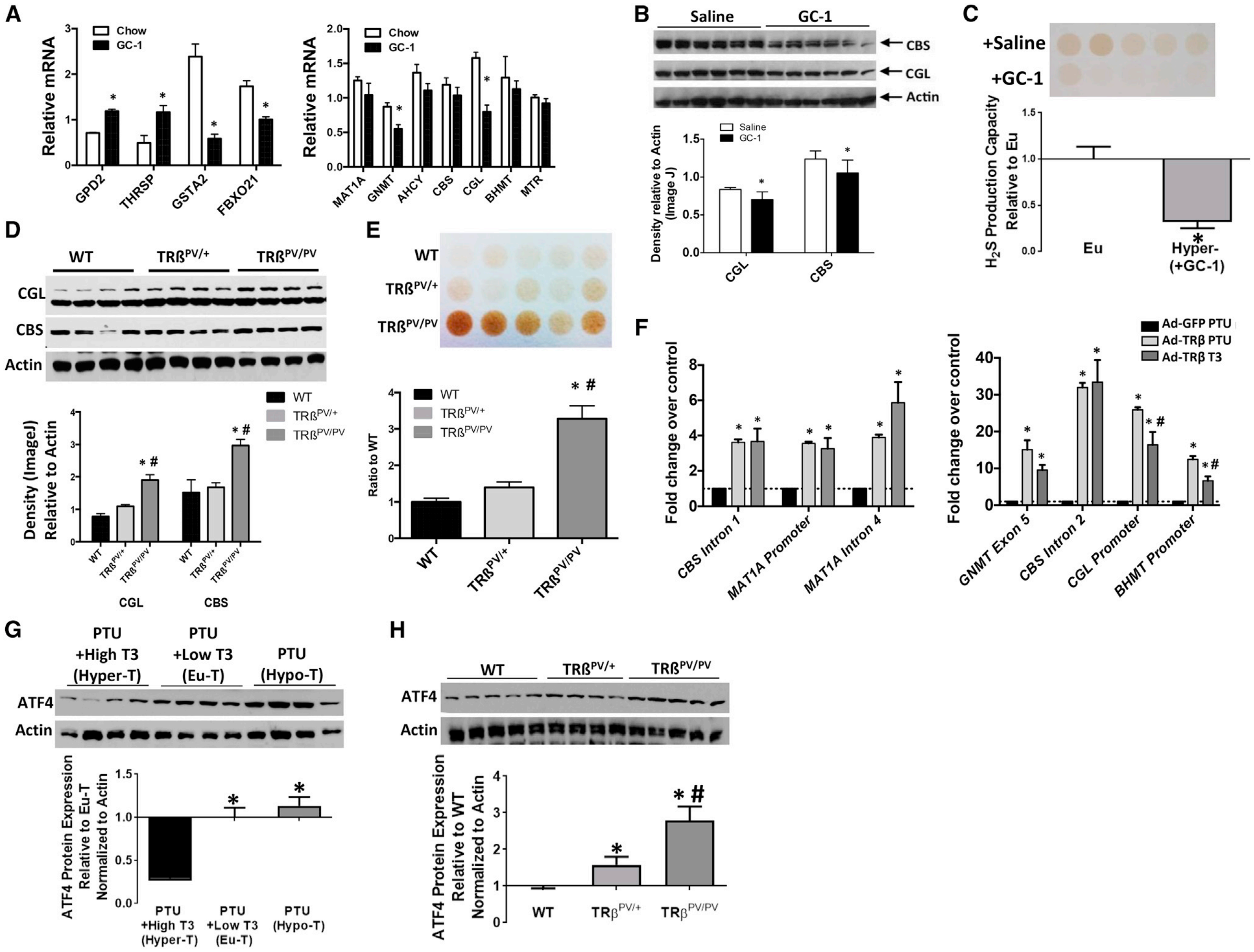

Figure 5. Thyroid Hormone Signaling through TRβ Represses Hepatic H_2S Production In Vivo

(A–C) Analysis of TH-responsive and sulfur amino acid metabolism-associated mRNA levels (n = 4–6) (A), protein expression (n = 6) (B), and H_2S production capacity (n = 5) (C) in livers of mice treated with GC-1 versus vehicle (saline) control. The asterisk indicates the significance of the difference between vehicle (saline) control (euthyroid) and +GC-1 groups (hyperthyroid); $^*p < 0.05$.

(D and E) Liver CBS and CGL protein expression (D) and H_2S production capacity (E) in mice with indicated TRβ status (WT, homozygous WT; $TR\beta^{PV/+}$, Het; $TR\beta^{PV/PV}$, homozygous mutant; n = 4–5/group). The asterisk indicates the significance of the difference between WT and $TR\beta^{PV/PV}$, and the # sign indicates the significance of the difference between $TR\beta^{PV/+}$ and $TR\beta^{PV/PV}$; $^{*/\#}p < 0.05$.

(F) Fold enrichment of TRβ binding to genetic regulator elements in sulfur amino acid metabolism and H_2S producing genes in the livers of mice infected with Ad-GFP (control) or Ad-TRβ while on PTU diets +/−T3 injection as indicated (n = 5/group). The asterisk indicates the significance of the difference between the Ad-GFP PTU and Ad-TRβ PTU or Ad-TRβ T3 groups, and the # sign indicates the significance of the difference between the Ad-TRβ PTU and Ad-TRβ T3 groups; $^{*/\#}p < 0.05$.

(G and H) Liver ATF4 protein expression in mice due to PTU/T3 administration (G) (n = 4/group) or TRβ mutations (H) (n = 4–5/group). The asterisks indicate the significance of the difference between Hyper-T and Eu-T or Hypo-T (G), or WT and $TR\beta^{PV/+}$ or $TR\beta^{PV/PV}$ (H), and the # sign indicates the significance of the difference between $TR\beta^{PV/+}$ and $TR\beta^{PV/PV}$ (H), $^{*/\#}p < 0.05$. Error bars are ± SEM. See also Figure S5.

hepatic H_2S production capacity (Figure 5C) and altered hepatic TSP metabolites, including cystathionine, similar to T_3 (Figure S5A).

The genetic requirement for TRβ1 in repression of hepatic TSP expression and H_2S production was tested in mice with a mutant and defective TRβ1 isoform ($TR\beta^{PV/PV}$) (Zhao et al., 2012). $TR\beta^{PV/PV}$ mice had higher CGL and CBS protein levels in the liver and enhanced H_2S production capacity than heterozygote or WT control animals (Figures 5D and 5E). Interestingly, neither CGL nor CBS mRNA levels were significantly altered in the $TR\beta^{PV/PV}$ mice compared with controls (Figure S5B), possibly due to long-term upregulation of this pathway in these animals. Thus, an intact TRβ regulates H_2S production, but it also remains possible that TRα plays a role in $TR\beta^{PV/PV}$ mutants.

Finally, to test the hypothesis that TRβ1 regulates H_2S production capacity by direct transcriptional control of TSP genes in vivo, we examined the TRβ cistrome that we generated previously (Ramadoss et al., 2014) for binding sites within regulatory regions of the CBS and CGL genes. Chromatin immunoprecipitation analysis revealed enrichment of TRβ binding at several

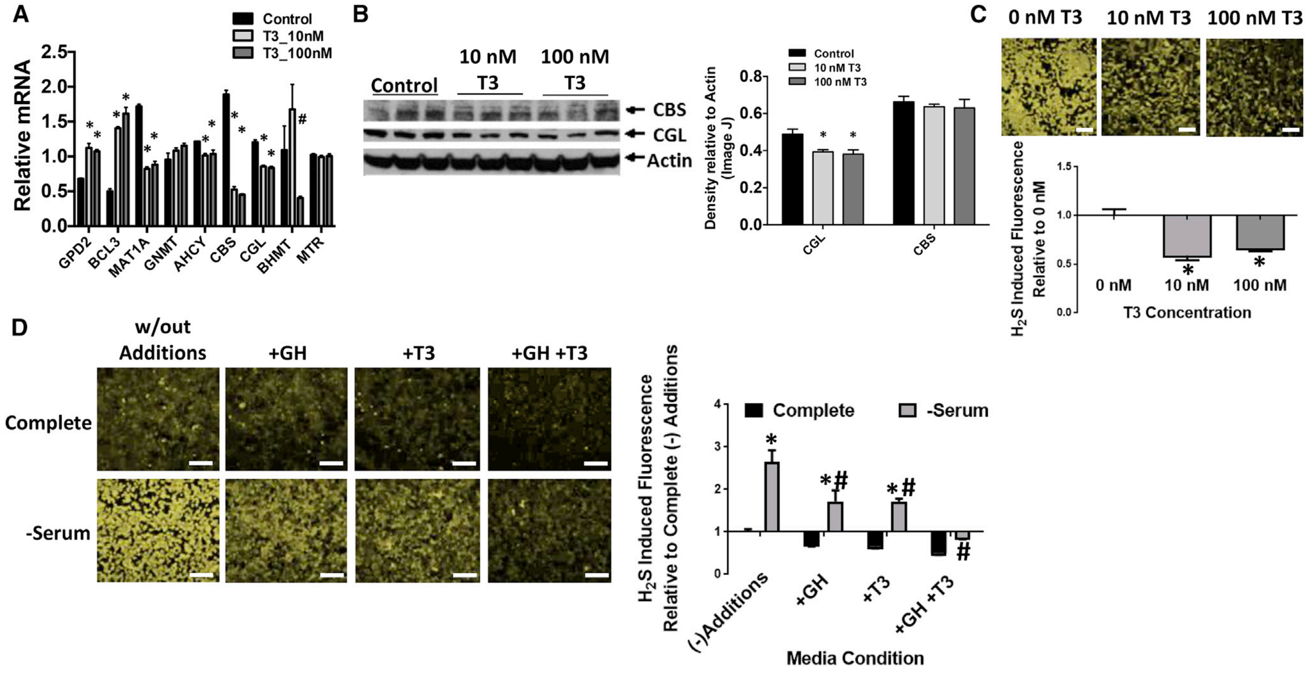

Figure 6. Additive Suppression of Hepatic H_2S Production by TH and GH In Vitro

(A–C) mRNA levels (n = 3) (A), protein expression (n = 3) (B), and endogenous H_2S production (n = 3) (C) in TH-responsive Hepa1-6 cells treated with T3 at the indicated concentration. The asterisk indicates the significance of the difference between vehicle control and T3 treatment and the hash indicates the significance of the difference between different T3 dosage groups; $^{*/\#}p < 0.05$.

(D) Endogenous H_2S production in TH-responsive Hepa1-6 cells grown in Complete medium or −Serum medium +/−GH +/−T3. The asterisk indicates the significance of the difference between Complete and −Serum within the GH/T3 treatment group, and the # sign indicates the significance of the difference between "w/out Additions" and +GH, +T3, or +GH+T3 groups within the −Serum grouping; $^{*/\#}p < 0.05$. Scale bars, 100 μm (C and D). Error bars are ± SEM. See also Figure S6.

loci on the CBS and CGL genes, as well as other genes of the methionine cycle (Figure 5F). Interestingly, T_3 injection resulted in a significant reduction in occupancy of TRβ on CGL and Bhmt promoters/enhancers, but not CBS binding sites. While the detailed mechanisms of negative regulation of hepatic TSP gene expression by T_3 and TRβ1 remain to be elucidated, these data suggest regulation of hepatic H_2S production via TRβ-dependent repression of TSP gene expression.

In addition to negative regulation by T_3/TRβ as described here, CGL gene expression is positively regulated by the stress response transcription factor ATF4 in reaction to cysteine restriction on the cell-autonomous level (Lee et al., 2008; Sikalidis et al., 2011) and in livers of mouse models of DR and Snell dwarfism (Li et al., 2014). Surprisingly, we found that reduction of global TH signaling via a PTU/LID diet (Figure 5G), or liver-specific signaling in $TR\beta^{PV/PV}$ mice (Figure 5H), increased hepatic ATF4 protein levels. Despite the increase in hepatic ATF4 protein in Snell dwarf mice (Figure S5C), we were not able to detect significantly elevated levels in GHRKO mice (Figure S5D). Thus, hyperthyroidism is associated with negative regulation of CGL expression through direct binding and repression of the CGL locus by T_3-bound TRβ1, while hypothyroidism is associated with increased CGL expression indirectly through derepression of the transcriptional activator ATF4, possibly by hypothyroidism-induced endoplasmic reticulum stress (Zhou et al., 2016).

Additive Suppression of Hepatic H_2S Production by TH and GH In Vitro

Having identified GH- and TH-dependent control of hepatic H_2S production capacity, we next sought to determine the potential interaction between the two in a tractable in vitro system. Because primary murine hepatocytes do not respond well to T_3, we employed a murine hepatic cell line, Hepa1-6, stably transfected with the TRβ isoform. Upon addition of T_3 to this cell line, known T_3 targets including gpd2 and bcl3 were induced, while methionine cycle genes mat1a and ahcy were repressed (Figure 6A). Importantly, both CGL and CBS were downregulated by T_3 in these cells (Figure 6A), although only CGL was regulated in a similar fashion at the protein level (Figure 6B). Endogenous H_2S production assessed using the P3 probe was reduced upon T_3 addition, confirming its direct ability to regulate endogenous H_2S through repression of CGL mRNA expression (Figure 6C).

We next tested the individual and combined contributions of GH and TH in regulation of endogenous H_2S production in this cell line. Overnight serum withdrawal resulted in a 2.5-fold increase in endogenous H_2S levels (Figure 6D). Addition of GH or TH individually each resulted in a 50% decrease in H_2S levels, while together they restored H_2S to baseline levels seen in cells maintained in full serum (Figure 6D). Finally, gene expression analysis confirmed the effects of TH, but not GH, on TSP gene expression (Figure S6). Taken together, GH and TH work

additively to suppress hepatic H_2S production in a cell-autonomous manner by distinct mechanisms.

CGL/H_2S Required for Negative Regulation of TH and GH/IGF-1 Signaling

What are the functional consequences of increased H_2S production in the context of reduced GH and/or TH signaling? We first approached this question by testing the genetic requirement for CGL in increased hepatic H_2S production observed upon hypothyroidism. CGL protein and H_2S production capacity were increased in hypothyroid WT mice on the PTU/LID diet compared with euthyroid WT mice on the normal diet, but were undetectable in littermate CGL KO mice on either diet (Figures 7A and 7B, and Figure S7A). No differences in food intake (Figure S7B), body weight (Figure S7C), or protein levels of the other H_2S-producting enzymes CBS and 3-MST (Figures 7A and S7A) were observed between genotypes. We conclude that CGL is required for increased hepatic H_2S production upon decreased TH signaling.

Surprisingly, CGL KO mice failed to achieve the same degree of hypothyroidism as WT controls on the PTU/LID diet. This was first observed systemically upon measuring circulating T_4, which remained elevated in CGL KO mice on the PTU/LID diet (Figure 7C), and was confirmed by TSHβ subunit gene expression in the pituitary (Figure 7D) and circulating TSH in the serum (Figure 7E), both of which failed to increase in CGL KO mice to WT levels on the PTU/LID diet.

These data suggest a functional role for CGL-derived H_2S in systemic feedback control of TH signaling, possibly through modulation of circulating T_4 levels. To test this further, CGL KO and WT mice were subject to fasting, a known suppressor of the hypothalamic-pituitary-thyroid axis on a more rapid timescale than the PTU/LID paradigm (Vella et al., 2011). While serum T_4 levels decreased in WT mice subject to a 3-day fast, T_4 levels actually increased slightly upon fasting in CGL KO mice (Figure 7F), despite similar weight loss between genotypes (Figure S7D). Taken together, these data suggest reciprocal negative regulation of H_2S production by TRβ-dependent repression of hepatic CGL gene transcription, and T_4 metabolism by CGL-derived H_2S.

We next considered the potential role of CGL-derived H_2S in negative regulation of global and hepatic GH action. In CGL KO mice, expression of pituitary GH mRNA (Figure 7G), hepatic GHR and IGF-1 mRNAs (Figure 7H), and circulating IGF-1 levels (Figure 7I) were inappropriately maintained and/or elevated upon PTU/LID relative to WT mice. Similar to its effects on circulating T_4, fasting significantly decreased serum IGF-1 in WT mice, but not in CGL KO mice (Figure 7J).

Finally, we tested the sufficiency of CGL-derived H_2S or chemical H_2S donors to lower GH signaling independent of PTU/LID or fasting. One week after adenoviral-mediated CGL overexpression in WT mice, which resulted in increased liver H_2S production capacity (Figure S7E), serum IGF-1 levels were significantly reduced relative to the adenoviral null control (Figure 7K). In CGL KO mice subjected to a 2-day fast, intraperitoneal administration of NaHS rescued the expected drop in serum IGF-1 levels (Figure 7L) and serum T_4 levels (Figure S7F). In WT mice, NaHS was sufficient to reduce circulating IGF-1 levels after acute injection (Figure S7G) or long-term treatment in the drinking water in combination with the slow-releasing H_2S donor GYY4137 (Lee et al., 2011) (Figure S7H). These findings are consistent with a role for CGL-derived endogenous H_2S in negative regulation of GH and TH signaling.

DISCUSSION

H_2S as a Common Endpoint in Models of Longevity and Metabolic Homeostasis

Reduced GH/IGF-1 action and DR represent the most widely studied classes of anti-aging models in rodents, but mechanisms underlying pleiotropic benefits on lifespan, healthspan, and stress resistance in these models, and the relationship between them, remain unclear. Previously we found that increased endogenous H_2S production upon DR is necessary and sufficient for surgical stress resistance in mice, and associated with extended longevity in fly, worm, and yeast models (Hine et al., 2015). Here, we found that increased endogenous hepatic H_2S production capacity was common to multiple long-lived mouse strains. As shown in the model in Figure 7M, GH and TH signaling were identified as negative regulators of hepatic H_2S production. Unexpectedly, CGL was required for downregulation of T_4 and IGF-1 in response to PTU/LID or fasting. Taken together, these data are consistent with increased hepatic H_2S as a common denominator among DR and reduced GH/IGF-1-based pro-longevity models. Further, it points to negative regulation of TH and GH/IGF-1 signaling as a potential mechanism of H_2S action in this context.

A limitation of this study lies in the quantitation of H_2S levels. Much of the data was based on H_2S production capacity using the lead acetate method, a measure of H_2S production under conditions in which exogenous substrate (L-Cys) and co-factor (VitB6) are supplied in excess. While specific for H_2S and sensitive for use in organ extracts with high production capacity, the more sensitive P3 fluorescent probe was required for measuring H_2S production in individual cells in tissue culture or frozen liver sections. Importantly, the P3 probe reports on free H_2S either generated de novo or released from sulfane sulfur without the addition of substrate or co-factor, thus making it possible to probe actual H_2S production with endogenous substrates. While neither method is suited for absolute quantitation of H_2S, both techniques showed increases in liver of Snell dwarf mice versus WT controls. Finally, while the importance of CGL-derived H_2S was addressed by exogenous H_2S supplementation in the context of CGL KO mice, our data in no way rule out potential contributions of other CGL-dependent metabolites such as glutathione.

Interactions between Nutrient Restriction, Endocrine Signaling, and H_2S Production

DR benefits overlap those of reduced GH/TH signaling, and indeed DR has been proposed to work, at least partially, the hypothalamic-pituitary axis, through modulation of somatotropic cells (Brown-Borg, 2015). The ability of DR to further extend longevity or improve metabolic fitness is blunted in most, but not all (Bartke et al., 2001), hypopituitary or GHRKO mouse studies (Arum et al., 2009; Bonkowski et al., 2006; Brown-Borg et al., 2014). In addition, GH supplementation concurrent with DR reverses some DR-related metabolic effects (Gesing et al.,

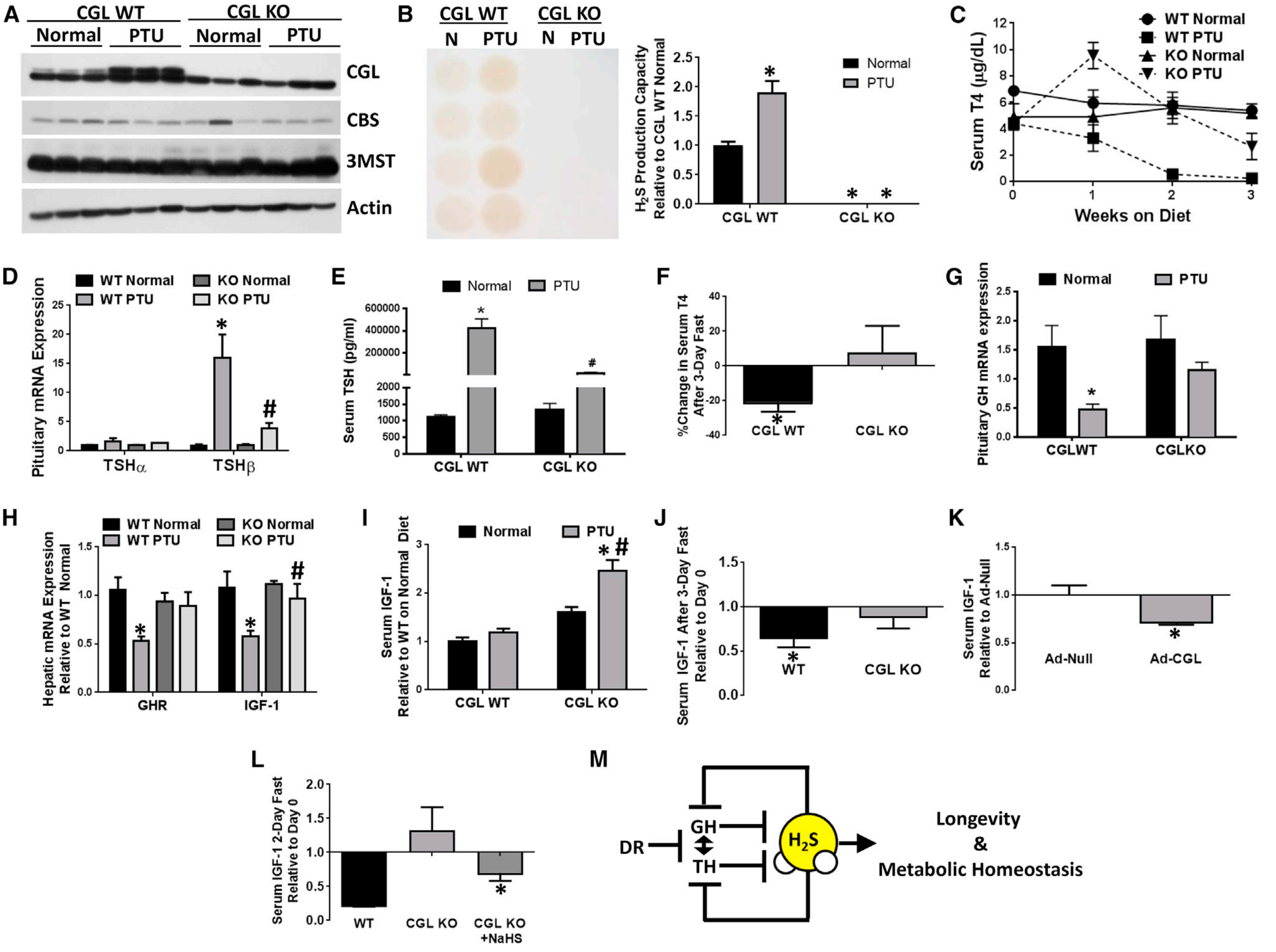

Figure 7. CGL/H_2S Is Required for Negative Regulation of TH and GH/IGF-I Signaling

(A and B) Western blot analysis of H_2S-producing enzymes CGL, CBS, and 3MST (n = 3) (A) and H_2S production capacity in livers of CGL WT and KO mice on Normal and PTU diets (n = 4–5) (B). The asterisk indicates the significance of the difference relative to CGL WT mice on the Normal diet; *$p < 0.05$.

(C) Serum T4 levels over a 3-week time course of Normal versus PTU diets in CGL WT and KO mice as indicated (n = 4–5).

(D) Pituitary mRNA expression of TSHα and TSHβ in CGL WT and KO mice after 3 weeks of Normal or PTU diet (n = 4–5). The asterisk indicates the significance of difference between diets within genotype, and the # sign indicates the significance of difference between CGL WT and CGL KO mice on the PTU diet; $^{*/\#}p < 0.05$.

(E) Serum TSH levels after 3 weeks of Normal or PTU diet in CGL WT and KO mice (n = 4–5). The asterisk indicates the significance of difference between diets within genotype, and the # sign indicates the significance of difference between CGL WT and CGL KO mice on the PTU diet; $^{*/\#}p < 0.05$.

(F) Percent change in serum T4 levels after 3 days of fasting in CGL WT and CGL KO mice (n = 3–4). The asterisk indicates the significance of the difference between day 0 and day 3 T4 levels; *$p < 0.05$.

(G–I) Pituitary GH mRNA expression (n = 4–5) (G), liver GHR and IGF-I mRNA expression (n = 4–5) (H), and serum IGF-1 (n = 4–5) (I) in CGL WT and KO mice after 3 weeks on a Normal or PTU diet as indicated. The asterisk indicates the significance of the difference between diets within genotype, and the # sign indicates the significance of the difference between genotypes on the PTU diet; $^{*/\#}p < 0.05$.

(J) Fold change in serum IGF-I between day 0 and day 3 of a 3-day fast in CGL WT and KO mice (n = 3–4/group). The asterisk indicates the significance of the difference between day 3 and day 0; *$p < 0.05$.

(K) Serum IGF-1 levels in mice 7 days after adenoviral infection with Ad-Null control or Ad-CGL overexpression adenovirus expressed relative to Ad-Null control (n = 4/group). The asterisk indicates the significance of the difference between Ad-Null and Ad-CGL; *$p < 0.05$.

(L) Fold change in serum IGF-1 after a 2-day fast in female CGL WT and KO mice with +/−NaHS supplementation in the CGL KOs (n = 4–5/group). The asterisks indicate the significance of the difference between serum IGF-1 levels on day 2 compared with day 0; *$p < 0.05$.

(M) Relationship between diet, GH/TH, and H_2S production.

Error bars are ± SEM. See also Figure S7.

2014). Hormonally, DR alters hypothalamic activity (Dacks et al., 2013), lowers GH (Fontana et al., 2008) and TH secretion (Fontana et al., 2006; Miller et al., 2005), and reduces hepatic GHR expression (Dauncey et al., 1994).

In addition to overlap between DR and reduced TH/GH signaling, genetic models of extended longevity associated with decreased GH/IGF-1 signaling also have decreased TH activity (Gesing et al., 2012). Conversely, reduction of TH

signaling results in decreased GHR production and signaling (Dieguez et al., 1986). Re-addition of these hormones into hypopituitary dwarf mice individually or in combination reverses many of the associated phenotypes (Do et al., 2015; Panici et al., 2010).

Despite these interconnections, here we found distinct, additive contributions of TH and GH to regulation of H_2S production independent of diet. TH directly regulated CGL and CBS gene expression via TRβ acting as a transcriptional repressor, while GH via GHR controlled H_2S production in an autophagy-dependent manner suggestive of substrate-level control. In addition, TH negatively regulated protein levels of the ATF4 transcription factor, a known direct activator of CGL expression (Mistry et al., 2016). Future experiments are required to determine the importance of these mechanisms to overall regulation of H_2S production and the contribution of increased H_2S to the individual and shared phenotypes associated with DR and reduced GH and/or TH signaling. Together, they represent potential targetable pathways toward harnessing endogenous H_2S production for beneficial outcomes.

Inhibition of TH and GH Activity as a Novel Mechanism of H_2S Action

Reduced insulin/IGF-1 signaling, often accompanied by reduced circulating levels of these hormones and increased sensitivity to their action, is a feature shared by multiple rodent models of extended longevity. Our finding that H_2S is required in certain contexts for downregulation of IGF-1 production suggests control of IGF-1 as a novel mechanism by which H_2S can exert its pleiotropic effects on health and longevity.

By what mechanism could H_2S control hepatic IGF-1 production? The observation that CGL was required for lowering circulating levels of T_4 and IGF-1 in two different models of hypothyroidism, combined with the ability of TH to activate GHR/IGF-1 gene expression (Koenig et al., 1987; Tsukada et al., 1998), suggests that the compromised ability to reduce circulating T_4 may be partially responsible. Consistent with the potential of H_2S to affect TH, H_2S donors present in garlic suppress TH signaling in rats (Tahiliani and Kar, 2003), and addition of NaHS blocks the production and/or secretion of GH from pituitary-derived cells (Mustafina et al., 2015). Similarly, we found that increased CGL expression and/or exogenous H_2S addition were sufficient to decrease circulating TH and IGF-1 in vivo (Figures 7 and S7).

Finally, we note that while a reduction in circulating IGF-1, which is produced mainly by liver, correlates with extended longevity, hepatic IGF-1 expression (and hence circulating IGF-1) can be reduced without longevity benefits, as in liver-specific IGF-1KO mice (Yakar et al., 1999). Future studies are required to determine the relative importance of hepatic H_2S production on lifespan, as well as the effects of TH/GH signaling on H_2S production in other tissue types.

Implications for Human Health

While GH and TH signaling both decrease with age, it remains unclear if these changes are adaptive or maladaptive, and if preventing this decline alters healthy aging. GH supplementation results in increased lean body mass, decreased fat mass, and gains in muscle strength. However, it is also associated with edema, carpal tunnel syndrome, joint pain, and an increase in type 2 diabetes and glucose intolerance (Liu et al., 2007). Increased TH results in DNA damage and premature senescence in vitro and in vivo (Zambrano et al., 2014). Here we show that GH and TH supplementation suppresses H_2S production. Because H_2S is positively correlated with improved stress resistance and health, our findings raise concerns about the use of GH and/or TH supplementation as anti-aging or performance-enhancing therapies.

Conclusions

Previously we found that increased endogenous H_2S production is in part responsible for the pleiotropic effects of DR. Here we found that TH and GH, two endocrine hormones associated with longevity control, are regulators of hepatic H_2S production. TH and GH independently and additively suppressed H_2S production through inhibition of CGL gene expression and control of substrate availability via autophagy, respectively. Unexpectedly, CGL-dependent H_2S itself was required for feedback regulation of TH signaling via negative regulation of T_4 levels and GH signaling through negative regulation of IGF-1. Together, these data point to H_2S as a potential downstream mediator of benefits shared between decreased GH/TH signaling and DR.

STAR★METHODS

Detailed methods are provided in the online version of this paper and include the following:

- KEY RESOURCES TABLE
- CONTACT FOR REAGENT AND RESOURCE SHARING
- EXPERIMENTAL MODELS
  - In Vivo Animal Studies
  - Cell Lines and In Vitro Tissue Culture Studies
- METHOD DETAILS
  - Lead Sulfide Method for Determination of H_2S Production Capacity
  - H_2S Detection with Fluorescent Probe
  - PTU/LID Diet for Altering Thyroid State
  - Western Blots/Protein Analysis
  - qPCR/mRNA Analysis
  - ChIP of TRβ
  - Liver Metabolomics
  - Detection of Serum Hormones: IGF-1, T4, TSH
- QUANTIFICATION AND STATISTICAL ANALYSIS

SUPPLEMENTAL INFORMATION

Supplemental Information includes seven figures and one table and can be found with this article online at http://dx.doi.org/10.1016/j.cmet.2017.05.003.

AUTHOR CONTRIBUTIONS

C.H., H.K., Y.Z., E.H., A.L., M.S.M., P.R., K.T.B., J.M.A., R.M., P.P., K.Y., V.L., P.C., A.B., R.M., J.R.M., and A.N.H. designed and/or performed experiments and analyzed data. J.M.A., C.K.O., S.C., S.S., K.H.A., A.K., F.M.F., P.P., D.J.W., C.S., R.W., K.Y., V.L., P.C., A.B., J.J.K., and R.M. contributed material resources. C.H., H.K., Y.Z., E.H., J.R.M., and A.N.H. wrote the manuscript. J.M.A., F.M.F., C.S., V.L., P.C., A.B., J.J.K., and R.M. edited the manuscript.

ACKNOWLEDGMENTS

We thank Nandan Nerurkar and Constance Cepko for providing support for 2-photon microscopy; Eli Lilly and Company for providing the FGF21OE mice; Thomas Scanlan for providing GC-1; and Gokhan Hotamisligil for providing ATGKO MEFs. This work was supported by the following grants: DK090629 and AG036712 (to J.R.M.); DK098525 and DK056123 (to A.N.H.); AG050777 (to C.H.); SNSF P1LAP3_158895 (to A.L.), AG019899 (to R.A.M.); 5P01CA120964 and 5P30CA006516 (to J.M.A.); AMA16GRNT27090006 (to C.K.O.); BBSRC BB/H012850/2 and the University of Glasgow (to C.S.).

Received: June 10, 2016
Revised: January 20, 2017
Accepted: May 11, 2017
Published: June 6, 2017

REFERENCES

Abe, K., and Kimura, H. (1996). The possible role of hydrogen sulfide as an endogenous neuromodulator. J. Neurosci. *16*, 1066–1071.

Ahluwalia, A., Clodfelter, K.H., and Waxman, D.J. (2004). Sexual dimorphism of rat liver gene expression: regulatory role of growth hormone revealed by deoxyribonucleic acid microarray analysis. Mol. Endocrinol. *18*, 747–760.

Arum, O., Bonkowski, M.S., Rocha, J.S., and Bartke, A. (2009). The growth hormone receptor gene-disrupted mouse fails to respond to an intermittent fasting diet. Aging Cell *8*, 756–760.

Atzmon, G., Barzilai, N., Surks, M.I., and Gabriely, I. (2009). Genetic predisposition to elevated serum thyrotropin is associated with exceptional longevity. J. Clin. Endocrinol. Metab. *94*, 4768–4775.

Bartke, A., and Brown-Borg, H. (2004). Life extension in the dwarf mouse. Curr. Top. Dev. Biol. *63*, 189–225.

Bartke, A., Wright, J.C., Mattison, J.A., Ingram, D.K., Miller, R.A., and Roth, G.S. (2001). Extending the lifespan of long-lived mice. Nature *414*, 412.

Bian, J.S., Yong, Q.C., Pan, T.T., Feng, Z.N., Ali, M.Y., Zhou, S., and Moore, P.K. (2006). Role of hydrogen sulfide in the cardioprotection caused by ischemic preconditioning in the rat heart and cardiac myocytes. J. Pharmacol. Exp. Ther. *316*, 670–678.

Biddinger, S.B., Hernandez-Ono, A., Rask-Madsen, C., Haas, J.T., Alemán, J.O., Suzuki, R., Scapa, E.F., Agarwal, C., Carey, M.C., Stephanopoulos, G., et al. (2008). Hepatic insulin resistance is sufficient to produce dyslipidemia and susceptibility to atherosclerosis. Cell Metab. *7*, 125–134.

Blackstone, E., and Roth, M.B. (2007). Suspended animation-like state protects mice from lethal hypoxia. Shock *27*, 370–372.

Bonkowski, M.S., Rocha, J.S., Masternak, M.M., Al Regaiey, K.A., and Bartke, A. (2006). Targeted disruption of growth hormone receptor interferes with the beneficial actions of calorie restriction. Proc. Natl. Acad. Sci. USA *103*, 7901–7905.

Brown-Borg, H.M. (2015). The somatotropic axis and longevity in mice. Am. J. Physiol. Endocrinol. Metab. *309*, E503–E510.

Brown-Borg, H.M., Rakoczy, S.G., Wonderlich, J.A., Rojanathammanee, L., Kopchick, J.J., Armstrong, V., and Raasakka, D. (2014). Growth hormone signaling is necessary for lifespan extension by dietary methionine. Aging Cell *13*, 1019–1027.

Cai, W.J., Wang, M.J., Moore, P.K., Jin, H.M., Yao, T., and Zhu, Y.C. (2007). The novel proangiogenic effect of hydrogen sulfide is dependent on Akt phosphorylation. Cardiovasc. Res. *76*, 29–40.

Chiellini, G., Apriletti, J.W., Yoshihara, H.A., Baxter, J.D., Ribeiro, R.C., and Scanlan, T.S. (1998). A high-affinity subtype-selective agonist ligand for the thyroid hormone receptor. Chem. Biol. *5*, 299–306.

Coschigano, K.T., Holland, A.N., Riders, M.E., List, E.O., Flyvbjerg, A., and Kopchick, J.J. (2003). Deletion, but not antagonism, of the mouse growth hormone receptor results in severely decreased body weights, insulin, and insulin-like growth factor I levels and increased life span. Endocrinology *144*, 3799–3810.

Dacks, P.A., Moreno, C.L., Kim, E.S., Marcellino, B.K., and Mobbs, C.V. (2013). Role of the hypothalamus in mediating protective effects of dietary restriction during aging. Front. Neuroendocrinol. *34*, 95–106.

Dauncey, M.J., Burton, K.A., White, P., Harrison, A.P., Gilmour, R.S., Duchamp, C., and Cattaneo, D. (1994). Nutritional regulation of growth hormone receptor gene expression. FASEB J. *8*, 81–88.

Dieguez, C., Jordan, V., Harris, P., Foord, S., Rodriguez-Arnao, M.D., Gomez-Pan, A., Hall, R., and Scanlon, M.F. (1986). Growth hormone responses to growth hormone-releasing factor (1-29) in euthyroid, hypothyroid and hyperthyroid rats. J. Endocrinol. *109*, 53–56.

Do, A., Menon, V., Zhi, X., Gesing, A., Wiesenborn, D.S., Spong, A., Sun, L., Bartke, A., and Masternak, M.M. (2015). Thyroxine modifies the effects of growth hormone in Ames dwarf mice. Aging (Albany NY) *7*, 241–255.

Dozmorov, I., Galecki, A., Chang, Y., Krzesicki, R., Vergara, M., and Miller, R.A. (2002). Gene expression profile of long-lived Snell dwarf mice. J. Gerontol. A Biol. Sci. Med. Sci. *57*, B99–B108.

Elrod, J.W., Calvert, J.W., Morrison, J., Doeller, J.E., Kraus, D.W., Tao, L., Jiao, X., Scalia, R., Kiss, L., Szabo, C., et al. (2007). Hydrogen sulfide attenuates myocardial ischemia-reperfusion injury by preservation of mitochondrial function. Proc. Natl. Acad. Sci. USA *104*, 15560–15565.

Fan, N.Y., Powers, C.A., and Stier, C.T. (1996). Lack of antidiuretic activity of lanreotide in the diabetes insipidus rat. J. Pharmacol. Exp. Ther. *276*, 875–881.

Fontana, L., Klein, S., Holloszy, J.O., and Premachandra, B.N. (2006). Effect of long-term calorie restriction with adequate protein and micronutrients on thyroid hormones. J. Clin. Endocrinol. Metab. *91*, 3232–3235.

Fontana, L., Weiss, E.P., Villareal, D.T., Klein, S., and Holloszy, J.O. (2008). Long-term effects of calorie or protein restriction on serum IGF-1 and IGFBP-3 concentration in humans. Aging Cell *7*, 681–687.

Gesing, A., Bartke, A., Masternak, M.M., Lewiński, A., and Karbownik-Lewińska, M. (2012). Decreased thyroid follicle size in dwarf mice may suggest the role of growth hormone signaling in thyroid growth regulation. Thyroid Res. *5*, 7.

Gesing, A., Al-Regaiey, K.A., Bartke, A., and Masternak, M.M. (2014). Growth hormone abolishes beneficial effects of calorie restriction in long-lived Ames dwarf mice. Exp. Gerontol. *58*, 219–229.

Gu, L., Liao, Z., Hoang, D.T., Dagvadorj, A., Gupta, S., Blackmon, S., Ellsworth, E., Talati, P., Leiby, B., Zinda, M., et al. (2013). Pharmacologic inhibition of Jak2-Stat5 signaling By Jak2 inhibitor AZD1480 potently suppresses growth of both primary and castrate-resistant prostate cancer. Clin. Cancer Res. *19*, 5658–5674.

Guevara-Aguirre, J., Balasubramanian, P., Guevara-Aguirre, M., Wei, M., Madia, F., Cheng, C.W., Hwang, D., Martin-Montalvo, A., Saavedra, J., Ingles, S., et al. (2011). Growth hormone receptor deficiency is associated with a major reduction in pro-aging signaling, cancer, and diabetes in humans. Sci. Transl. Med. *3*, 70ra13.

Harputlugil, E., Hine, C., Vargas, D., Robertson, L., Manning, B.D., and Mitchell, J.R. (2014). The TSC complex is required for the benefits of dietary protein restriction on stress resistance in vivo. Cell Rep. *8*, 1160–1170.

Hauck, S.J., Hunter, W.S., Danilovich, N., Kopchick, J.J., and Bartke, A. (2001). Reduced levels of thyroid hormones, insulin, and glucose, and lower body core temperature in the growth hormone receptor/binding protein knockout mouse. Exp. Biol. Med. (Maywood) *226*, 552–558.

Hine, C., Harputlugil, E., Zhang, Y., Ruckenstuhl, C., Lee, B.C., Brace, L., Longchamp, A., Treviño-Villarreal, J.H., Mejia, P., Ozaki, C.K., et al. (2015). Endogenous hydrogen sulfide production is essential for dietary restriction benefits. Cell *160*, 132–144.

Inagaki, T., Lin, V.Y., Goetz, R., Mohammadi, M., Mangelsdorf, D.J., and Kliewer, S.A. (2008). Inhibition of growth hormone signaling by the fasting-induced hormone FGF21. Cell Metab. *8*, 77–83.

Kabil, O., Vitvitsky, V., Xie, P., and Banerjee, R. (2011). The quantitative significance of the transsulfuration enzymes for H2S production in murine tissues. Antioxid. Redox Signal. *15*, 363–372.

Kato, Y., Ying, H., Willingham, M.C., and Cheng, S.Y. (2004). A tumor suppressor role for thyroid hormone beta receptor in a mouse model of thyroid carcinogenesis. Endocrinology *145*, 4430–4438.

Kharitonenkov, A., Shiyanova, T.L., Koester, A., Ford, A.M., Micanovic, R., Galbreath, E.J., Sandusky, G.E., Hammond, L.J., Moyers, J.S., Owens, R.A., et al. (2005). FGF-21 as a novel metabolic regulator. J. Clin. Invest. *115*, 1627–1635.

Kimura, Y., and Kimura, H. (2004). Hydrogen sulfide protects neurons from oxidative stress. FASEB J. *18*, 1165–1167.

Koenig, R.J., Brent, G.A., Warne, R.L., Larsen, P.R., and Moore, D.D. (1987). Thyroid hormone receptor binds to a site in the rat growth hormone promoter required for induction by thyroid hormone. Proc. Natl. Acad. Sci. USA *84*, 5670–5674.

Kuhn, J.M., Legrand, A., Ruiz, J.M., Obach, R., De Ronzan, J., and Thomas, F. (1994). Pharmacokinetic and pharmacodynamic properties of a long-acting formulation of the new somatostatin analogue, lanreotide, in normal healthy volunteers. Br. J. Clin. Pharmacol. *38*, 213–219.

Lee, J.I., Dominy, J.E., Sikalidis, A.K., Hirschberger, L.L., Wang, W., and Stipanuk, M.H. (2008). HepG2/C3A cells respond to cysteine deprivation by induction of the amino acid deprivation/integrated stress response pathway. Physiol. Genomics *33*, 218–229.

Lee, Z.W., Zhou, J., Chen, C.S., Zhao, Y., Tan, C.H., Li, L., Moore, P.K., and Deng, L.W. (2011). The slow-releasing hydrogen sulfide donor, GYY4137, exhibits novel anti-cancer effects in vitro and in vivo. PLoS One *6*, e21077.

Lee, C., Wan, J., Miyazaki, B., Fang, Y., Guevara-Aguirre, J., Yen, K., Longo, V., Bartke, A., and Cohen, P. (2014). IGF-I regulates the age-dependent signaling peptide humanin. Aging Cell *13*, 958–961.

Li, W., Li, X., and Miller, R.A. (2014). ATF4 activity: a common feature shared by many kinds of slow-aging mice. Aging Cell *13*, 1012–1018.

Liang, L., Zhou, T., Jiang, J., Pierce, J.H., Gustafson, T.A., and Frank, S.J. (1999). Insulin receptor substrate-1 enhances growth hormone-induced proliferation. Endocrinology *140*, 1972–1983.

Liu, H., Bravata, D.M., Olkin, I., Nayak, S., Roberts, B., Garber, A.M., and Hoffman, A.R. (2007). Systematic review: the safety and efficacy of growth hormone in the healthy elderly. Ann. Intern. Med. *146*, 104–115.

Liu, Y., Yang, R., Liu, X., Zhou, Y., Qu, C., Kikuiri, T., Wang, S., Zandi, E., Du, J., Ambudkar, I.S., et al. (2014). Hydrogen sulfide maintains mesenchymal stem cell function and bone homeostasis via regulation of Ca(2+) channel sulfhydration. Cell Stem Cell *15*, 66–78.

Majtan, T., Pey, A.L., Fernández, R., Fernández, J.A., Martínez-Cruz, L.A., and Kraus, J.P. (2014). Domain organization, catalysis and regulation of eukaryotic cystathionine beta-synthases. PLoS One *9*, e105290.

Miller, D.L., and Roth, M.B. (2007). Hydrogen sulfide increases thermotolerance and lifespan in *Caenorhabditis elegans*. Proc. Natl. Acad. Sci. USA *104*, 20618–20622.

Miller, R.A., Buehner, G., Chang, Y., Harper, J.M., Sigler, R., and Smith-Wheelock, M. (2005). Methionine-deficient diet extends mouse lifespan, slows immune and lens aging, alters glucose, T4, IGF-I and insulin levels, and increases hepatocyte MIF levels and stress resistance. Aging Cell *4*, 119–125.

Mistry, R.K., Murray, T.V., Prysyazhna, O., Martin, D., Burgoyne, J.R., Santos, C., Eaton, P., Shah, A.M., and Brewer, A.C. (2016). Transcriptional regulation of cystathionine-γ-lyase in endothelial cells by NADPH oxidase 4-dependent signaling. J. Biol. Chem. *291*, 1774–1788.

Mustafina, A.N., Yakovlev, A.V., Gaifullina, A.Sh., Weiger, T.M., Hermann, A., and Sitdikova, G.F. (2015). Hydrogen sulfide induces hyperpolarization and decreases the exocytosis of secretory granules of rat GH3 pituitary tumor cells. Biochem. Biophys. Res. Commun. *465*, 825–831.

Nakano, S., Ishii, I., Shinmura, K., Tamaki, K., Hishiki, T., Akahoshi, N., Ida, T., Nakanishi, T., Kamata, S., Kumagai, Y., et al. (2015). Hyperhomocysteinemia abrogates fasting-induced cardioprotection against ischemia/reperfusion by limiting bioavailability of hydrogen sulfide anions. J. Mol. Med. (Berl.) *93*, 879–889.

Olson, K.R., Dombkowski, R.A., Russell, M.J., Doellman, M.M., Head, S.K., Whitfield, N.L., and Madden, J.A. (2006). Hydrogen sulfide as an oxygen sensor/transducer in vertebrate hypoxic vasoconstriction and hypoxic vasodilation. J. Exp. Biol. *209*, 4011–4023.

Panici, J.A., Harper, J.M., Miller, R.A., Bartke, A., Spong, A., and Masternak, M.M. (2010). Early life growth hormone treatment shortens longevity and decreases cellular stress resistance in long-lived mutant mice. FASEB J. *24*, 5073–5079.

Papapetropoulos, A., Pyriochou, A., Altaany, Z., Yang, G., Marazioti, A., Zhou, Z., Jeschke, M.G., Branski, L.K., Herndon, D.N., Wang, R., et al. (2009). Hydrogen sulfide is an endogenous stimulator of angiogenesis. Proc. Natl. Acad. Sci. USA *106*, 21972–21977.

Paul, B.D., Sbodio, J.I., Xu, R., Vandiver, M.S., Cha, J.Y., Snowman, A.M., and Snyder, S.H. (2014). Cystathionine γ-lyase deficiency mediates neurodegeneration in Huntington's disease. Nature *509*, 96–100.

Ramadoss, P., Abraham, B.J., Tsai, L., Zhou, Y., Costa-e-Sousa, R.H., Ye, F., Bilban, M., Zhao, K., and Hollenberg, A.N. (2014). Novel mechanism of positive versus negative regulation by thyroid hormone receptor β1 (TRβ1) identified by genome-wide profiling of binding sites in mouse liver. J. Biol. Chem. *289*, 1313–1328.

Rozing, M.P., Houwing-Duistermaat, J.J., Slagboom, P.E., Beekman, M., Frölich, M., de Craen, A.J., Westendorp, R.G., and van Heemst, D. (2010). Familial longevity is associated with decreased thyroid function. J. Clin. Endocrinol. Metab. *95*, 4979–4984.

Selman, C., Lingard, S., Choudhury, A.I., Batterham, R.L., Claret, M., Clements, M., Ramadani, F., Okkenhaug, K., Schuster, E., Blanc, E., et al. (2008). Evidence for lifespan extension and delayed age-related biomarkers in insulin receptor substrate 1 null mice. FASEB J. *22*, 807–818.

Selman, C., Partridge, L., and Withers, D.J. (2011). Replication of extended lifespan phenotype in mice with deletion of insulin receptor substrate 1. PLoS One *6*, e16144.

Sikalidis, A.K., and Stipanuk, M.H. (2010). Growing rats respond to a sulfur amino acid-deficient diet by phosphorylation of the alpha subunit of eukaryotic initiation factor 2 heterotrimeric complex and induction of adaptive components of the integrated stress response. J. Nutr. *140*, 1080–1085.

Sikalidis, A.K., Lee, J.I., and Stipanuk, M.H. (2011). Gene expression and integrated stress response in HepG2/C3A cells cultured in amino acid deficient medium. Amino Acids *41*, 159–171.

Singha, S., Kim, D., Moon, H., Wang, T., Kim, K.H., Shin, Y.H., Jung, J., Seo, E., Lee, S.J., and Ahn, K.H. (2015). Toward a selective, sensitive, fast-responsive, and biocompatible two-photon probe for hydrogen sulfide in live cells. Anal. Chem. *87*, 1188–1195.

Szabó, C., and Papapetropoulos, A. (2011). Hydrogen sulphide and angiogenesis: mechanisms and applications. Br. J. Pharmacol. *164*, 853–865.

Tahiliani, P., and Kar, A. (2003). The combined effects of Trigonella and Allium extracts in the regulation of hyperthyroidism in rats. Phytomedicine *10*, 665–668.

Tsukada, A., Ohkubo, T., Sakaguchi, K., Tanaka, M., Nakashima, K., Hayashida, Y., Wakita, M., and Hoshino, S. (1998). Thyroid hormones are involved in insulin-like growth factor-I (IGF-I) production by stimulating hepatic growth hormone receptor (GHR) gene expression in the chicken. Growth Horm. IGF Res. *8*, 235–242.

Vella, K.R., Ramadoss, P., Lam, F.S., Harris, J.C., Ye, F.D., Same, P.D., O'Neill, N.F., Maratos-Flier, E., and Hollenberg, A.N. (2011). NPY and MC4R signaling regulate thyroid hormone levels during fasting through both central and peripheral pathways. Cell Metab. *14*, 780–790.

Wang, R. (2012). Physiological implications of hydrogen sulfide: a whiff exploration that blossomed. Physiol. Rev. *92*, 791–896.

Wang, M., and Miller, R.A. (2012). Fibroblasts from long-lived mutant mice exhibit increased autophagy and lower TOR activity after nutrient deprivation or oxidative stress. Aging Cell *11*, 668–674.

Yakar, S., Liu, J.L., Stannard, B., Butler, A., Accili, D., Sauer, B., and LeRoith, D. (1999). Normal growth and development in the absence of hepatic insulin-like growth factor I. Proc. Natl. Acad. Sci. USA *96*, 7324–7329.

Yang, W., Yang, G., Jia, X., Wu, L., and Wang, R. (2005). Activation of KATP channels by H2S in rat insulin-secreting cells and the underlying mechanisms. J. Physiol. *569*, 519–531.

Yang, G., Wu, L., Jiang, B., Yang, W., Qi, J., Cao, K., Meng, Q., Mustafa, A.K., Mu, W., Zhang, S., et al. (2008). H2S as a physiologic vasorelaxant: hypertension in mice with deletion of cystathionine gamma-lyase. Science *322*, 587–590.

Yang, L., Li, P., Fu, S., Calay, E.S., and Hotamisligil, G.S. (2010). Defective hepatic autophagy in obesity promotes ER stress and causes insulin resistance. Cell Metab. *11*, 467–478.

Yuan, M., Breitkopf, S.B., Yang, X., and Asara, J.M. (2012). A positive/negative ion-switching, targeted mass spectrometry-based metabolomics platform for bodily fluids, cells, and fresh and fixed tissue. Nat. Protoc. *7*, 872–881.

Zambrano, A., García-Carpizo, V., Gallardo, M.E., Villamuera, R., Gómez-Ferrería, M.A., Pascual, A., Buisine, N., Sachs, L.M., Garesse, R., and Aranda, A. (2014). The thyroid hormone receptor β induces DNA damage and premature senescence. J. Cell Biol. *204*, 129–146.

Zanardo, R.C., Brancaleone, V., Distrutti, E., Fiorucci, S., Cirino, G., and Wallace, J.L. (2006). Hydrogen sulfide is an endogenous modulator of leukocyte-mediated inflammation. FASEB J. *20*, 2118–2120.

Zhang, Y., Xie, Y., Berglund, E.D., Coate, K.C., He, T.T., Katafuchi, T., Xiao, G., Potthoff, M.J., Wei, W., Wan, Y., et al. (2012). The starvation hormone, fibroblast growth factor-21, extends lifespan in mice. Elife *1*, e00065.

Zhao, W., Zhang, J., Lu, Y., and Wang, R. (2001). The vasorelaxant effect of H(2)S as a novel endogenous gaseous K(ATP) channel opener. EMBO J. *20*, 6008–6016.

Zhao, L., Zhu, X., Won Park, J., Fozzatti, L., Willingham, M., and Cheng, S.Y. (2012). Role of TSH in the spontaneous development of asymmetrical thyroid carcinoma in mice with a targeted mutation in a single allele of the thyroid hormone-β receptor. Endocrinology *153*, 5090–5100.

Zhao, K., Li, H., Li, S., and Yang, G. (2014). Regulation of cystathionine gamma-lyase/H_2S system and its pathological implication. Front. Biosci. (Landmark Ed.) *19*, 1355–1369.

Zhou, L., Ding, S., Li, Y., Wang, L., Chen, W., Bo, T., Wu, K., Li, C., Liu, X., Zhao, J., et al. (2016). Endoplasmic reticulum stress may play a pivotal role in lipid metabolic disorders in a novel mouse model of subclinical hypothyroidism. Sci. Rep. *6*, 31381.

STAR★METHODS

KEY RESOURCES TABLE

REAGENT or RESOURCE	SOURCE	IDENTIFIER
Antibodies		
Anti-CGL (Anti-Cystathionase)	Abcam	Ab151769
Anti-CBS	Abcam	Ab135626
Anti-3MST (Anti-MPST)	Sigma	HPA001240
Anti-Stat5	Santa Cruz	Sc-835
Anti-p-Stat5	Cell Signaling Technology	9359
Anti-GNMT	Aviva	ARP43565_P050
Anti-AHCY	Abcam	Ab56146
Anti-ATF4 (Anti-CREB-2)	Santa Cruz	Sc-200
Anti ATG5	Novus	NB110-53818
Anti ATG7	Sigma	A2856
Anti-beta Tubulin	Cell Signaling	2128
Anti-Actin	Cell Signaling	4970
HRP conjugated anti-rabbit	Dako	P044801-2
Bacterial and Virus Strains		
Ad-CMV-CGL (Ad-mCTH)	Vector Biolabs	ADV-256305
Ad-CMV-Null	Vector Biolabs	1300
Lentiviral sh-GFP	Laboratory of Dr. Alec Kimmelman	N/A
Lentiviral sh-ATG5	Laboratory of Dr. Alec Kimmelman	N/A
Lentiviral sh-ATG7	Laboratory of Dr. Alec Kimmelman	N/A
Biological Samples		
Livers (frozen) taken from experimental mouse strains listed in the Experimental Models: Organisms/Strains section	See Experimental Models: Organisms/Strains section	See Experimental Models: Organisms/Strains section
Serum/Plasma (frozen) taken from experimental mouse strains listed in the Experimental Models: Organisms/ Strains section	See Experimental Models: Organisms/Strains section	See Experimental Models: Organisms/Strains section
Chemicals, Peptides, and Recombinant Proteins		
NaHS	Sigma	161527
GYY4137	Sigma	SML0100
Lanreotide	Sigma	SML0132
PTU/LID diet	Harlan Teklad	TD 95125
T3 (Triiodo-L—thyronine)	Sigma	T2752
GC-1	Laboratory of Dr. Thomas Scanlan	Chiellini et al., 1998
GH (growth hormone)	Sigma	S8648
FGF21	Genscript	Z03290
Bafilomycin	Sigma	B1793
Chloroquine	Sigma	C6628
DL-Propargylglycine	Sigma	P7888
Aminooxyacetic acid	Sigma	C13408
AZD1480	Selleckchem	S2162
Passive Lysis Buffer (5x)	Promega	E1941
PLP (Pyridoxal 5′-phosphate)	Sigma	P9255
L-cysteine	Sigma	C7352
lead (II) acetate trihydrate	Sigma	316512

(*Continued on next page*)

Continued		
REAGENT or RESOURCE	SOURCE	IDENTIFIER
P3 H2S Detection Probe	From the lab of Prof. K.H. Ahn	Singha et al., 2015
Critical Commercial Assays		
Mouse/Rat IGF-1 ELISA kit	R&D Systems	SMG100
T4 ELISA kit	Diagnostic Automation/ Cortez Diagnostics, Inc	3149-18
MILLIPLEX MAP Mouse Endocrine (TSH) Assay	EMD Millipore	MPTMAG-49K
Experimental Models: Cell Lines		
Hepa1-6 2Cl BirA/TRbeta	Laboratory of Dr. Anthony N. Hollenberg	N/A
Hepa1-6 (mouse liver hepatoma)	ATCC	CRL-1830
Primary mouse hepatocytes prepared from C57BL/6 mice (freshly isolated in the lab of Dr. James Mitchell for each experiment)	Jackson Laboratories and laboratory of Dr. James R. Mitchell	000664 and this paper
ATG5 Knockout mouse embryonic fibroblasts	From the laboratory of Dr. Gokhan Hotamisligil	Yang et al., 2010
ATG7 Knockout mouse embryonic fibroblasts	From the laboratory of Dr. Gokhan Hotamisligil	Yang et al., 2010
Primary CGL WT and KO mouse tail dermal fibroblasts	From the laboratory of Dr. James R. Mitchell	This paper
Experimental Models: Organisms/Strains		
129/C57BL/6 background WT and KO CGL Male and Female Mice	Laboratories of Dr. Rui Wang and Dr. James R. Mitchell	Hine et al., 2015; Yang et al., 2008
WT and LirKO Female Mice	Laboratory of Dr. James R. Mitchell	Harputlugil et al., 2014
Male and Female WT and Snell Dwarf mice	Laboratory of Dr. Richard Miller	Dozmorov et al., 2002
Female WT and Ames Dwarf mice	Laboratory of Dr. Andrzej Bartke	Panici et al., 2010
Male and Female WT and GHRKO Mice	Laboratory of Dr. Richard Miller	Wang and Miller, 2012
Male and Female WT and IRS-1 KO mice	Laboratory of Dr. Colin Selman	Selman et al., 2008
Male WT and FGF21 overexpressing mice	Laboratory of Dr. Pavlos Pissios	Kharitonenkov et al., 2005
TRbeta NI/NI, NI/PV, and mutant PV/PV mice	Laboratory of Dr. Sheu-yann Cheng	(Kato et al., 2004
C57BL/6 mice	Jackson Laboratories	000664
B6D2F1 hybrid mice	Jackson Laboratories	100006
Oligonucleotides		
IGF-1 F: TGCTTGCTCACCTTCACCA IGF-1 R: CAACACTCATCCACAATGCC	N/A	N/A
GHR F: ATTCACCAAGTGTCGTTCCC GHR R: TCCATTCCTGGGTCCATTCA	N/A	N/A
CGL F: TTGGATCGAAACACCCACAAA CGL R: AGCCGACTATTGAGGTCATCA	N/A	N/A
CBS F: GGGACAAGGATCGAGTCTGGA CBS R: AGCACTGTGTGATAATGTGGG	N/A	N/A
HPRT F:TTTCCCTGGTTAAGCAGTACA GCCC HPRT R:TGGCCTGTATCCAACAC TTCGAGA	N/A	N/A
RPL13 F:TTCGGCTGAAGCCTACCA GAAAGT RPL13 R:TCTTCCGATAGT GCATCTTGGCCT	N/A	N/A
MAT1A F: GATAGCAGATCTGAGGCGCT MAT1A R: TGCACCATTATCCTGCATGT	N/A	N/A
GNMT F: AAGAGGGCTTCAGCGTGATG GNMT R: CTGGCAAGTGAGCAAAACTGT	N/A	N/A

(*Continued on next page*)

Continued		
REAGENT or RESOURCE	SOURCE	IDENTIFIER
AHCY F: CGCCAGCATGTCTGATAAAC<br>AHCY R: CCTGGCATCTCATTCTCAGC	N/A	N/A
BHMT F: TTAGAACGCTTAAATGCCGGAG<br>BHMT R: GATGAAGCTGACGAACTGCCT	N/A	N/A
For a full list of all primers used, please see Table S1		
Software and Algorithms		
ImageJ	National Institutes of Health	Windows version, https://imagej.nih.gov/ij/download.html

CONTACT FOR REAGENT AND RESOURCE SHARING

Further information and requests for resources and reagents should be directed to and will be fulfilled by the Lead Contact, James R. Mitchell (jmitchel@hsph.harvard.edu)

EXPERIMENTAL MODELS

In Vivo Animal Studies

All experiments were performed with the approval of the Institutional Animal Care and Use Committee (IACUC) from the respective institutions. Except where indicated in this paper or cited in the respective references, animals were bred and maintained under standard housing conditions with *ad libitum* access to food (Purina 5058) and water, 12-hour light/12-hour dark cycles, temperature between 20–23°C with 30%–70% relative humidity, and weaned from their mothers between 3-4 weeks of age. Mice used included: young adult (10-15 week old) male and (1-year old) female CGLKO and WT control mice on a mixed 129/C57BL/6 background (Hine et al., 2015; Yang et al., 2008); female (10-16 week old) LIrKO and control mice generated by crossing $Ir^{fl/fl}$ (WT) mice with $Ir^{fl/fl}$|$Albumin\text{-}Cre^{+/-}$ (LIrKO) mice as previously described (Harputlugil et al., 2014); male and female Snell Dwarf and WT littermates aged 4-5 months (Dozmorov et al., 2002); 18-month female Ames Dwarf and WT littermates treated daily with recombinant growth hormone during a 6-week period early in life (weeks 2-8) as described (Panici et al., 2010), male and female GHRKO and WT littermates aged 7-8 months (Wang and Miller, 2012), sixteen week old male and female IRS-1 KO and WT littermates (Selman et al., 2008); male FGF21 overexpressing (OE) transgenic and WT littermates (Kharitonenkov et al., 2005) and TRβ WT (NI/NI), heterozygote (NI/PV) and Mutant (PV/PV) mice as previously described (Kato et al., 2004).

Lanreotide (Sigma) was administered to 10 week old male WT B6D2F1 hybrids (Jackson Labs) via daily sub-cutaneous injection at 0.4 mg/kg (Fan et al., 1996) in sterile saline once/day for 7 days prior to euthanasia and organ harvest. CGL was overexpressed by IV injection of 10^{10} PFUs of Ad-CMV-CGL (ADV-256305) or control Ad-CMV-Null virus (Vector Biolabs) into 10 week old male WT B6D2F1 hybrids as previously described (Hine et al., 2015) 7 days prior to blood serum collection for IGF-1 determination. Hydrogen sulfide supplementation was performed in male C57BL/6 mice (Jackson) using NaHS (Sigma) (1mM) and GYY4137 (Sigma) (260μM) in the drinking water starting at 10 weeks of age for 2 weeks, with supplementation of additional NaHS every two days and GYY4137 after the first week and with blood taken for analysis on day 0, 7, and 14. Young adult (10 to 15 weeks of age) male CGL WT and KO mice were fasted of food for three days with *ad libitum* access to drinking water and blood/serum sampled at Day 0 and Day 3. 1-year old female CGL WT and KO mice were fasted of food for two-days and given *ad libitum* access to drinking water, and then blood taken on Day 0 and Day 2, with one of the two CGL KO groups receiving NaHS injection in sterile saline at a dose of 5mg/kg per injection after the first blood draw on day 0, day 1, and just prior to harvest on day 2. 10-week old male B6D2F1 hybrids were kept on an *ad libitum* rodent diet and injected with NaHS in sterile saline at 5mg/kg and blood/serum taken 24-hours later for analysis. Recombinant human IGF-1 (rhIGF-1) at 500 μg/kg/d or recombinant human growth hormone (rhGH) at 2 mg kg/day (BID) were administered by intraperitoneal injection for 20 days in 12-week-old male C57BL/6 mice as described (Lee et al., 2014). Modulation of thyroid hormone levels on a hypothyroid background was accomplished in 9-week old male C57BL6 mice maintained for 3 weeks on a PTU/LID diet (Harlan Teklad formula # TD 95125) to induce hypothyroidism followed by saline or T3 (Sigma, St. Louis, MO) intraperitoneal injection once a day for 4 days to create hypothyroid (saline), euthyroid (0.5 μg/100g bodyweight of T3) or hyperthyroid (25 μg/100g bodyweight of T3) mice. For modulation of thyroid hormone levels on a euthyroid background, 10 week old male mice fed a standard rodent diet (Labdiet Chow) were injected intraperitoneally with saline or 25 μg/100g bodyweight of T3 once a day for 4 days. GC-1 (Chiellini et al., 1998) was administered to 10-week old male C57BL6 mice fed a standard rodent diet (Labdiet Chow) by intraperitoneal injection (3 μg/100g bodyweight) once a day for 4 days.

Cell Lines and In Vitro Tissue Culture Studies

Mouse primary hepatocytes from 8-10 week old female WT C57BL/6 mice (Jackson Laboratories) were isolated via portal vein collagenase treatment (Liberase, Roche) followed by Percoll gradient centrifugation and culturing in William's E media with 5% FBS at

37°C, 20% O_2 and 5% CO_2. The Hepa1-6 cell line, originally obtained from a C57L mouse (sex unknown), was maintained in DMEM media with 10% FBS. Hepa1-6 2Cl BirA/TRβ cells, which were generated by sequentially transfecting BirA And TRβ into Hepa 1-6 cells and selecting clones using geneticin and puromycin, were maintained in DMEM+Glutamax with 10% vol/vol FBS supplemented with Gibco Antibiotic-Antimycotic, Geneticin, and Puromycin at 37°C, 20% O_2 and 5% CO_2. Lentiviral infections of Hepa1-6 cells with sh-GFP, sh-ATG5 or sh-ATG7 were performed in the presence of 10% FBS and 8μg/ml Polybrene on two consecutive days; infected Hepa1-6 cells were selected with 3μg/ml puromycin for 2 weeks before use and maintained with 3μg/ml puromycin. Genetically deficient ATG5 fibroblasts originally from 129/C57BL mixed background embryos (sex unknown) and ATG7 fibroblasts originally from C57BL/6 embryos (sex unknown) were a gift from the lab of Dr. Gokhan Hotamisligil and previously reported (Yang et al., 2010). CGL WT and KO mouse dermal fibroblasts from tail skin were prepared from female CGL WT and KO mice and maintained in 20% FBS in DMEM +Penicillin/Streptomycin. For differential H_2S determination, an equal number of cells were seeded onto 12-well plates and incubated overnight in media +/- serum with or without the following drugs/hormones: GH (0.1-1μg/mL, Sigma), FGF21 (100nM, Genscript), T3 (10&100nM, Sigma), Bafilomycin (1μM, Sigma), Chloroquine (10μM, Sigma), DL-Propargylglycine (PAG) (100μM, Sigma), Aminooxyacetic acid (AOAA) (100μM, Sigma), AZD1480 (10μM, Selleckchem).

METHOD DETAILS

Lead Sulfide Method for Determination of H_2S Production Capacity

H_2S production capacity in liver homogenates was measured as previously described (Hine et al., 2015). Briefly, fresh or flash frozen liver was homogenized in passive lysis buffer (Promega) and volume normalized to protein content. An equal volume/protein amount was added to a reaction master mix containing PBS, 1mM Pyridoxal 5′-phosphate (PLP) (Sigma) and 10mM Cys (Sigma) and placed in a well-format (12-well to 96-well) plate. H_2S detection paper, saturated with lead acetate and then dried, was placed above the plate and incubated 1-2hrs at 37°C until H_2S in the gas phase reacted with the paper to form dark lead sulfide. In live primary hepatocytes, growth media was supplemented with 10mM Cys and 10μM PLP, and a lead acetate H_2S detection paper placed over the plate for 2-24hrs at 37°C in a CO_2 incubator.

H_2S Detection with Fluorescent Probe

Cultured Cells

The chemical H_2S probe P3 (Singha et al., 2015) was added at 10μM final concentration directly to the cell culture media following overnight treatment of cells (e.g. +/- serum, +/- hormones/drugs). One hour after P3 addition, H_2S-activated P3 fluorescence was quantitated on a BioTek plate reader (excitation 360nm, emission 528nm). Alternately, cells were washed 1x with PBS, fixed in ice-cold methanol for 10 minutes, dried, and stored at -20°C until imaging via 2-photon microscopy (Mai Tai, Spectra-Physics) with excitation at 880 nm and emission 520-530nm using a 10x objective following rehydration in 1x PBS. Images were analyzed using the Integrated Density (IntDen) function of ImageJ software to determine the total florescence of the entire image.

Liver Ex Vivo *Analysis*

Flash frozen liver from adult male WT or Snell Dwarf mice was embedded in OCT media and cryosectioned onto glass slides, thawed to room temp and incubated in PBS containing 10μM P3 at room temperature for 30 minutes prior to fixation and then subsequent imaging via 2-photon microscopy using a 60x objective. The fluorescence signal was determined using the Integrated Density (IntDen) function of ImageJ obtained from analyzing the entire image and averaging three images per animal and three animals/group. These values were then normalized to the average wildtype control value (set to 1).

PTU/LID Diet for Altering Thyroid State

10-15 week old WT or CGLKO male mice were given *ad libitum* access to the Normal control 5058 diet (Purina), or the low iodine diet with 0.15% PTU; PTU/LID (Harlan Teklad) for three weeks prior to assay or harvest.

Western Blots/Protein Analysis

Protein analysis was performed via western blot on tissue and cell homogenates in passive lysis buffer (Promega), separated by SDS-PAGE, transferred to PVDF membrane (Whatman) and blotted for CGL (ab151769 Abcam), CBS (ab135626 Abcam), 3MST (HPA001240 Sigma), Stat5 (sc-835 Santa Cruz), p-Stat5 (#9359 Cell Signaling Technology), GNMT (Aviva), AHCY (Abcam ab56146), ATF4 (aka CREB-2 C-20, Santa Cruz Biotechnology sc-200), ATG5 (Novus, NB110-53818), ATG7 (Sigma, A2856), β-Tubulin 9F3 (#2128 Cell Signaling) or Actin (#4970 Cell Signaling) followed by HRP conjugated secondary anti-rabbit antibody (Dako).

qPCR/mRNA Analysis

Total RNA was isolated from tissues and cells using standard phenol-chloroform/isopropanol extraction and cDNA synthesized by random hexamer priming with the Verso cDNA kit (Thermo). qRT-PCR was performed with SYBR green dye (Lonza) and TaqPro DNA polymerase (Denville) or Taqman Universal PCR Master Mix (Thermo Fisher Scientific). Fold changes were calculated by the $\Delta\Delta C_t$ method using the genes Hprt and/or Rpl13 as controls and ultimately normalized to the control for each respective experiment, or normalized to a standard curve utilizing cyclophilin as an internal control. Primer sets used for PCR are listed in the Key Resources Table and in Table S1.

ChIP of TRβ

TRβ1-binding sites in gnmt, cth, cbs, mat1a and bhmt were identified in a previous study that characterized the cistrome of TRβ1 in mouse liver (Ramadoss et al., 2014). ChIP-qPCR was performed as previously described. Briefly, hypothyroid mice that expressed BirA ubiquitously were transduced with an adenovirus expressing wither GFP alone or Blrp-TRβ1 and GFP together. Subsequently half the hypothyroid mice in each group were given T3 injections to render them hyperthyroid. Livers from these mice were collected for chromatin affinity precipitation using streptavidin-agarose beads and qPCR was performed using primer sequences directed against genomic sites identified by ChIP-seq analysis.

Liver Metabolomics

Ex vivo mouse liver polar metabolomics was performed using targeted tandem mass spectrometry (LC-MS/MS) with polarity switching and selected reaction monitoring (SRM) with a AB/SCIEX 5500 QTRAP Mass spectrometer as previously described in (Yuan et al., 2012).

Detection of Serum Hormones: IGF-1, T4, TSH

Serum IGF-1 was detected using the IGF-1 Mouse/Rat ELISA kit (R&D Systems) following the manufacturer's recommendations. Total plasma T4 levels were measured using a commercially available ELISA Kit (Diagnostic Automation/ Cortez Diagnostics, Inc Calabasas, CA). Thyroid-stimulating hormone (TSH) was measured in plasma via Milliplex MAP (mutianalyte panels) (mouse thyroid hormone TSH panel; EMD Millipore, Billerica, MA).

QUANTIFICATION AND STATISTICAL ANALYSIS

Data are displayed as means +/- standard error of the mean (SEM) and statistical significance assessed in GraphPad Prism and/or Microsoft Excel using Student's t tests to compare values between two specific groups, and one-way or two-way ANOVA followed by Tukey's or Sidak's Multiple Comparisons Tests when comparing more than two groups/variables at a given time. A P-value of 0.05 or less was deemed statistically significant in all of these statistical tests. Statistical details and results of experiments are found in the figures and figure legends. Quantification of western blot images, lead sulfide H_2S production capacity assays, and 2-photon H_2S production images was done using the IntDen measurement in ImageJ software and normalized to the respective control group in each experiment when applicable. All experiments examining hepatic H_2S production were initially performed in a blinded fashion and technical repeats were done at least twice. Snell Dwarf and GHRKO experiments were repeated twice independently for a total of 6/genotype per sex or 12 total/genotype, and PTU/LID diet experiments in CGL WT and KO mice were repeated independently three times for a total of 11-12 animals/group.

Cell Metabolism, Volume *25*

Supplemental Information

Hypothalamic-Pituitary Axis Regulates Hydrogen Sulfide Production

Christopher Hine, Hyo-Jeong Kim, Yan Zhu, Eylul Harputlugil, Alban Longchamp, Marina Souza Matos, Preeti Ramadoss, Kevin Bauerle, Lear Brace, John M. Asara, C. Keith Ozaki, Sheue-yann Cheng, Subhankar Singha, Kyo Han Ahn, Alec Kimmelman, Ffolliott M. Fisher, Pavlos Pissios, Dominic J. Withers, Colin Selman, Rui Wang, Kelvin Yen, Valter D. Longo, Pinchas Cohen, Andrzej Bartke, John J. Kopchick, Richard Miller, Anthony N. Hollenberg, and James R. Mitchell

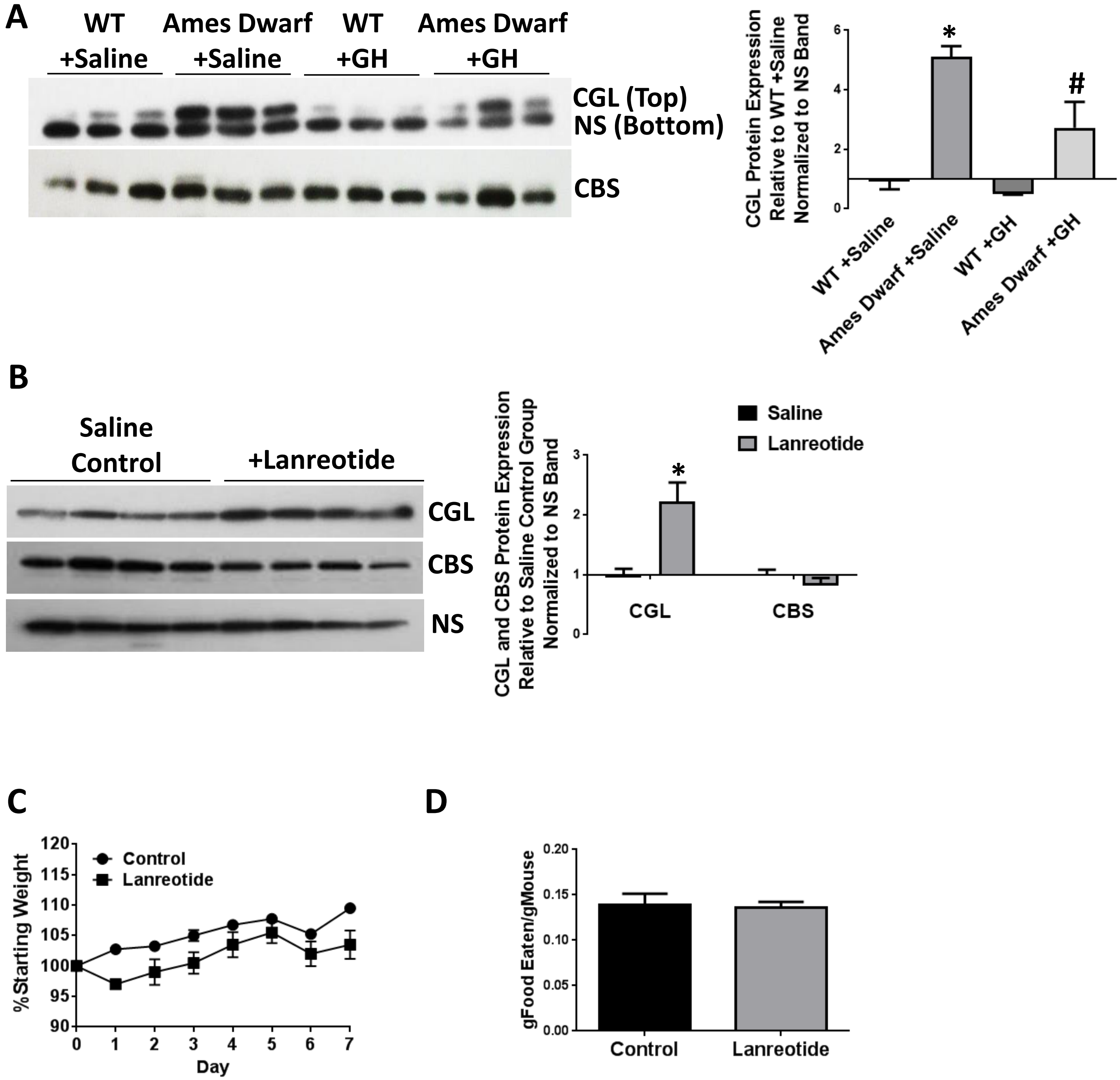
A
WT +Saline
Ames Dwarf +Saline
WT +GH
Ames Dwarf +GH
CGL (Top)
NS (Bottom)
CBS
CGL Protein Expression Relative to WT +Saline Normalized to NS Band
*
#
WT +Saline
Ames Dwarf +Saline
WT +GH
Ames Dwarf +GH
B
Saline Control
+Lanreotide
CGL
CBS
NS
CGL and CBS Protein Expression Relative to Saline Control Group Normalized to NS Band
Saline
Lanreotide
CGL
CBS
C
Control
Lanreotide
%Starting Weight
Day
D
gFood Eaten/gMouse
Control
Lanreotide

Figure S1

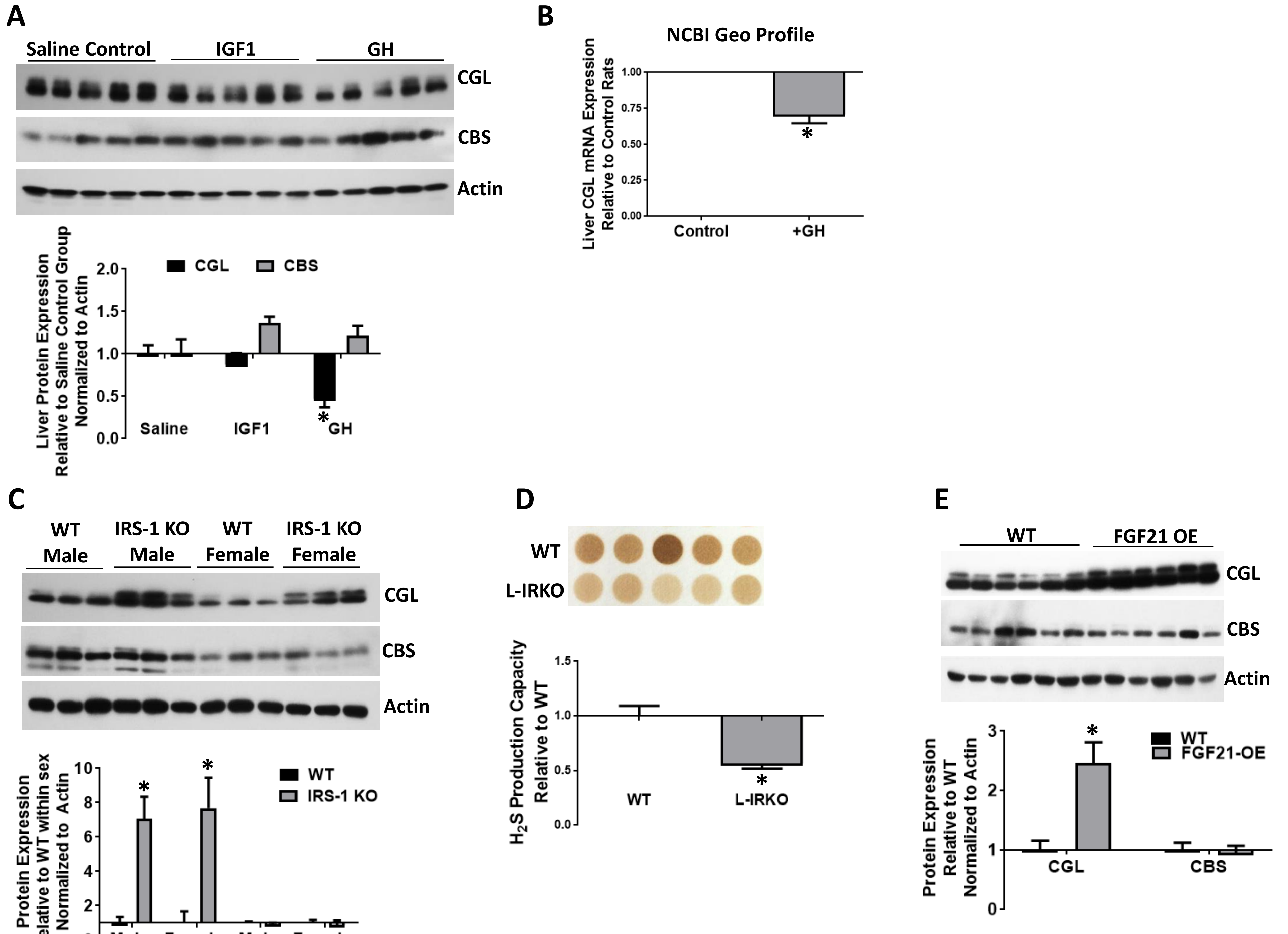
A
Saline Control
IGF1
GH
CGL
CBS
Actin
Liver Protein Expression Relative to Saline Control Group Normalized to Actin
CGL
CBS
Saline
IGF1
GH
B
NCBI Geo Profile
Liver CGL mRNA Expression Relative to Control Rats
Control
+GH
C
WT Male
IRS-1 KO Male
WT Female
IRS-1 KO Female
CGL
CBS
Actin
Protein Expression Relative to WT within sex Normalized to Actin
WT
IRS-1 KO
Male CGL
Female CGL
Male CBS
Female CBS
D
WT
L-IRKO
H2S Production Capacity Relative to WT
WT
L-IRKO
E
WT
FGF21 OE
CGL
CBS
Actin
Protein Expression Relative to WT Normalized to Actin
WT
FGF21-OE
CGL
CBS

Figure S2

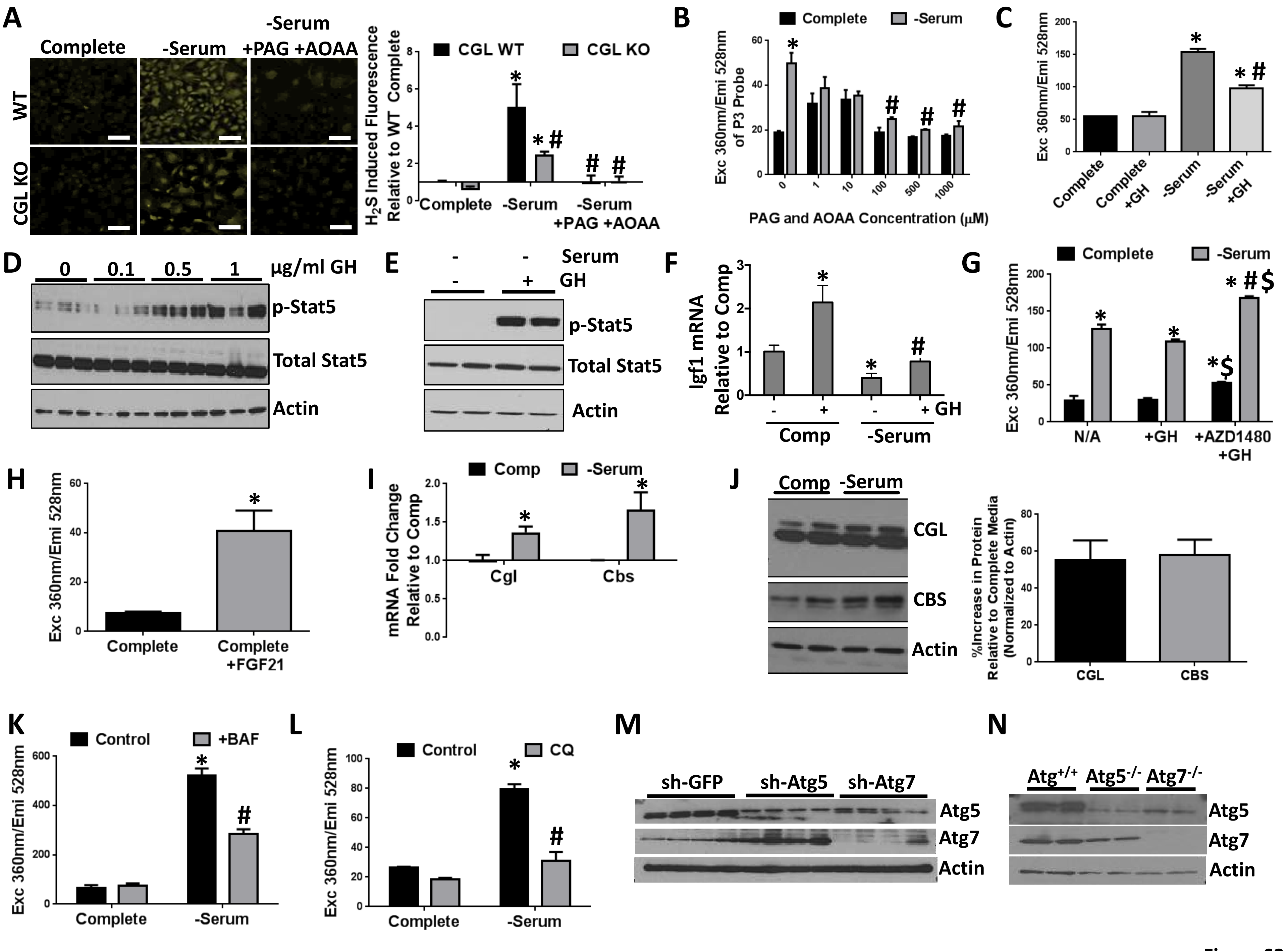
A
Complete
-Serum
-Serum +PAG +AOAA
WT
CGL KO
CGL WT
CGL KO
H2S Induced Fluorescence Relative to WT Complete
Complete
-Serum
-Serum +PAG +AOAA
B
Complete
-Serum
Exc 360nm/Emi 528nm of P3 Probe
PAG and AOAA Concentration (μM)
C
Exc 360nm/Emi 528nm
Complete
Complete +GH
-Serum
-Serum +GH
D
0
0.1
0.5
1
μg/ml GH
p-Stat5
Total Stat5
Actin
E
Serum
GH
p-Stat5
Total Stat5
Actin
F
Igf1 mRNA Relative to Comp
Comp
-Serum
GH
G
Complete
-Serum
Exc 360nm/Emi 528nm
N/A
+GH
+AZD1480 +GH
H
Exc 360nm/Emi 528nm
Complete
Complete +FGF21
I
Comp
-Serum
mRNA Fold Change Relative to Comp
Cgl
Cbs
J
Comp
-Serum
CGL
CBS
Actin
%Increase in Protein Relative to Complete Media (Normalized to Actin)
CGL
CBS
K
Control
+BAF
Exc 360nm/Emi 528nm
Complete
-Serum
L
Control
CQ
Exc 360nm/Emi 528nm
Complete
-Serum
M
sh-GFP
sh-Atg5
sh-Atg7
Atg5
Atg7
Actin
N
Atg+/+
Atg5-/-
Atg7-/-
Atg5
Atg7
Actin

Figure S3

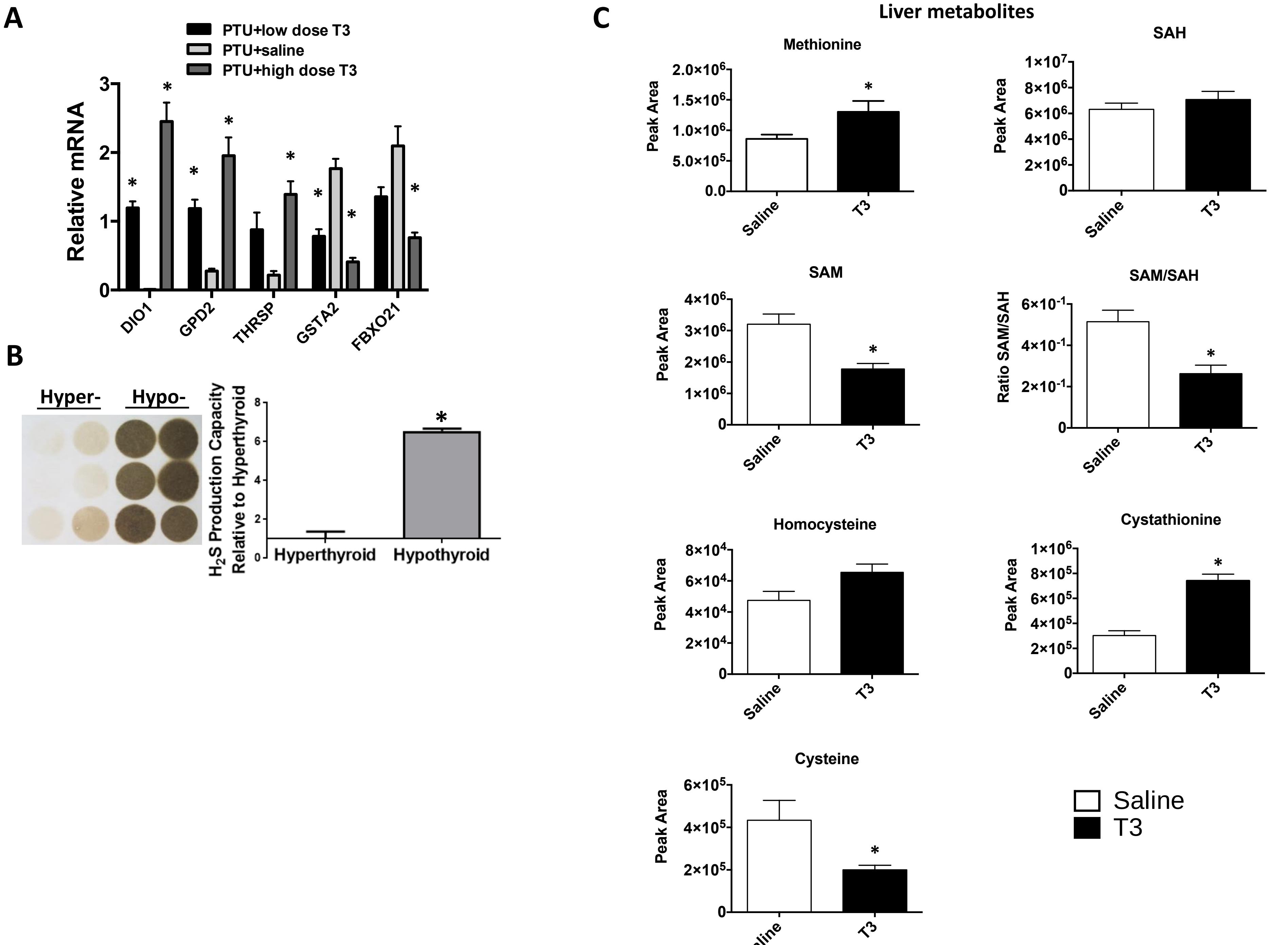
A
PTU+low dose T3
PTU+saline
PTU+high dose T3
Relative mRNA
DIO1
GPD2
THRSP
GSTA2
FBXO21
B
Hyper-
Hypo-
H2S Production Capacity Relative to Hyperthyroid
Hyperthyroid
Hypothyroid
C
Liver metabolites
Methionine
SAH
SAM
SAM/SAH
Homocysteine
Cystathionine
Cysteine
Peak Area
Ratio SAM/SAH
Saline
T3

Figure S4

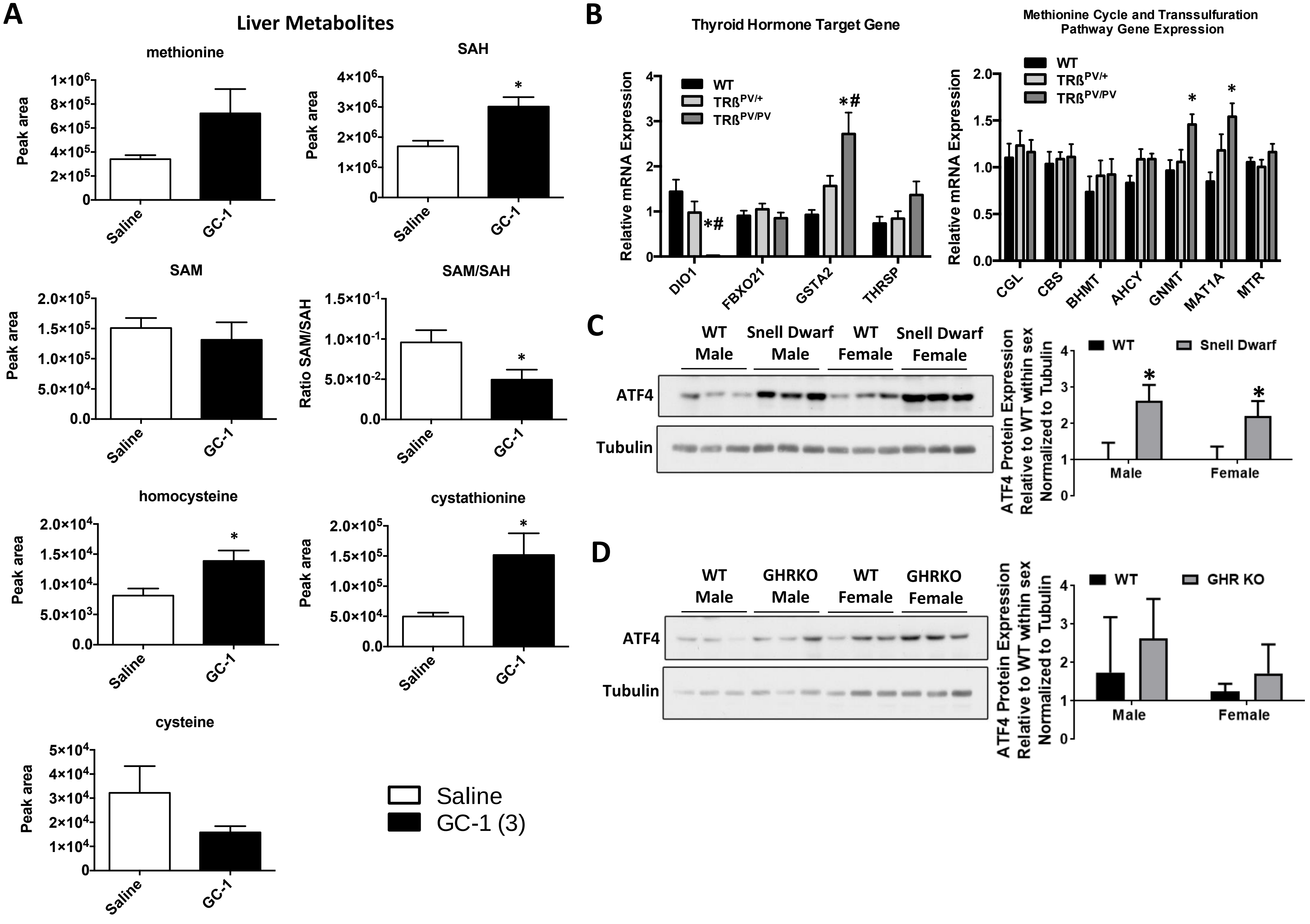
A
Liver Metabolites
methionine
SAH
SAM
SAM/SAH
homocysteine
cystathionine
cysteine
Peak area
Ratio SAM/SAH
Saline
GC-1
Saline
GC-1 (3)
B
Thyroid Hormone Target Gene
Methionine Cycle and Transsulfuration Pathway Gene Expression
Relative mRNA Expression
WT
TRß PV/+
TRß PV/PV
DIO1
FBXO21
GSTA2
THRSP
CGL
CBS
BHMT
AHCY
GNMT
MAT1A
MTR
C
WT Male
Snell Dwarf Male
WT Female
Snell Dwarf Female
ATF4
Tubulin
ATF4 Protein Expression Relative to WT within sex Normalized to Tubulin
WT
Snell Dwarf
Male
Female
D
WT Male
GHRKO Male
WT Female
GHRKO Female
ATF4
Tubulin
ATF4 Protein Expression Relative to WT within sex Normalized to Tubulin
WT
GHR KO
Male
Female

Figure S5

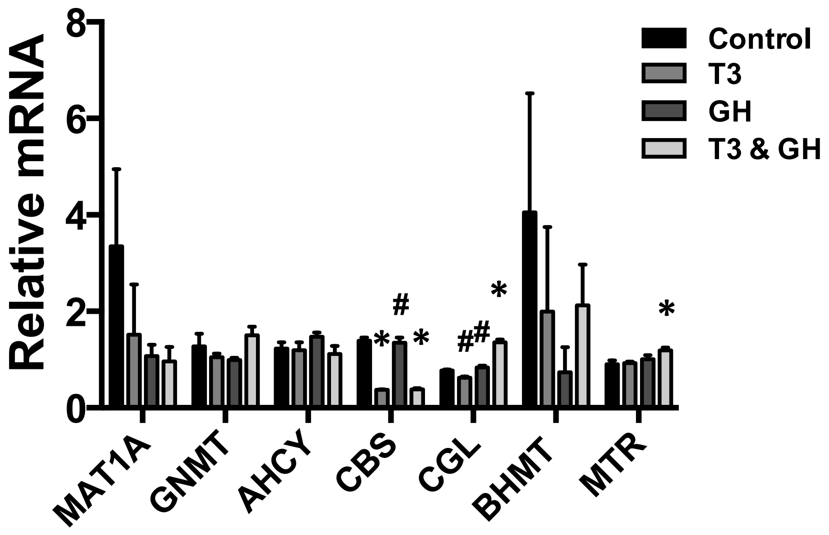

Relative mRNA
8
6
4
2
0
Control
T3
GH
T3 & GH
MAT1A
GNMT
AHCY
CBS
CGL
BHMT
MTR
*
#
*
#
#
*
*

Figure S6

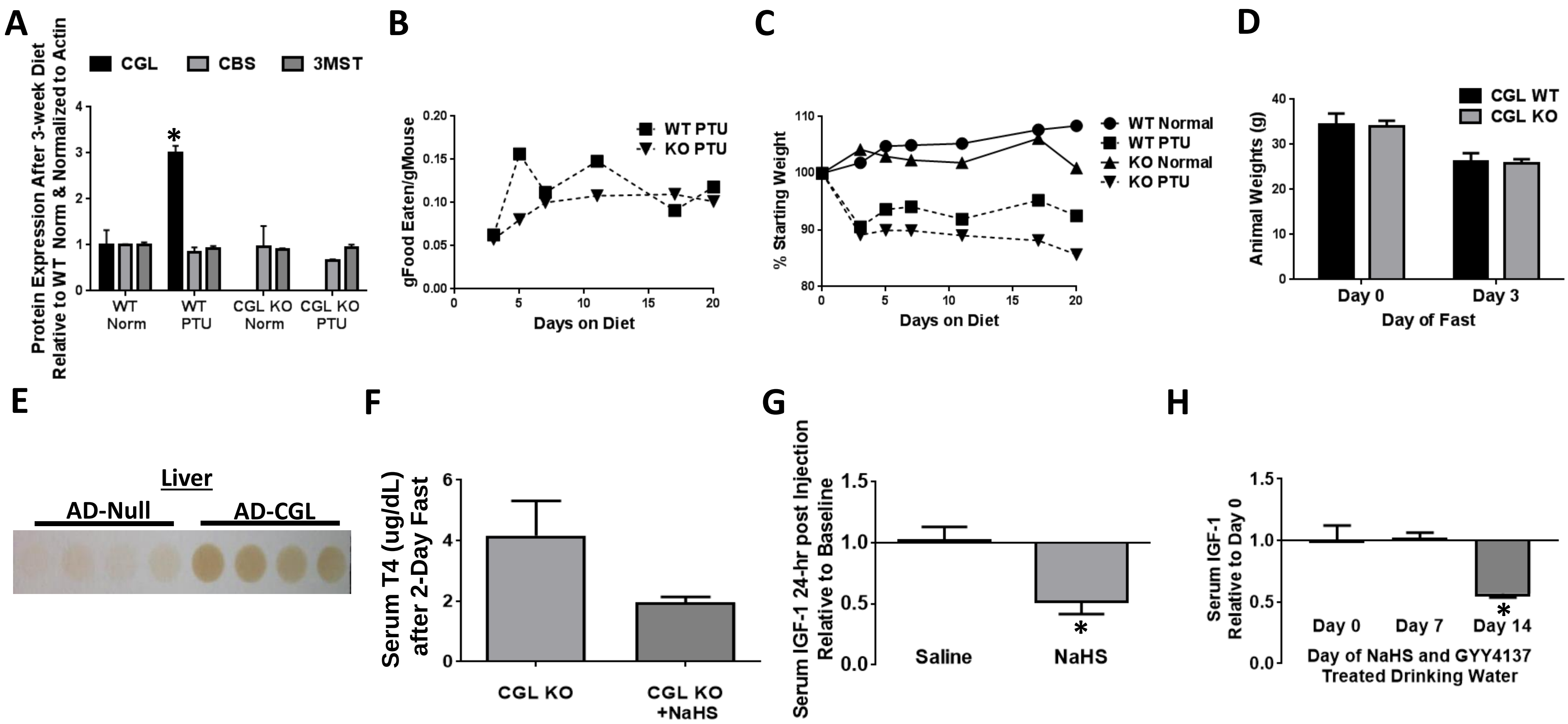
A
CGL
CBS
3MST
Protein Expression After 3-week Diet Relative to WT Norm & Normalized to Actin
WT Norm
WT PTU
CGL KO Norm
CGL KO PTU
B
WT PTU
KO PTU
gFood Eaten/gMouse
Days on Diet
C
WT Normal
WT PTU
KO Normal
KO PTU
% Starting Weight
Days on Diet
D
CGL WT
CGL KO
Animal Weights (g)
Day 0
Day 3
Day of Fast
E
Liver
AD-Null
AD-CGL
F
Serum T4 (ug/dL) after 2-Day Fast
CGL KO
CGL KO +NaHS
G
Serum IGF-1 24-hr post Injection Relative to Baseline
Saline
NaHS
H
Serum IGF-1 Relative to Day 0
Day 0
Day 7
Day 14
Day of NaHS and GYY4137 Treated Drinking Water

Figure S7

Supplemental Figure Legends:

Figure S1: Pharmacological impact on hepatic CGL and CBS expression in WT and Dwarf mice. (A) Liver CGL and CBS protein expression in18-month old Ames Dwarf and WT mice after early life saline or growth hormone (GH) injection (n=3/group). Asterisk indicates the significance of the difference between WT +Saline and Ames Dwarf +Saline and pound indicates the significance of the difference between +Saline and +GH in the Ames Dwarf groups, */#p<0.05. **(B)** Liver CGL and CBS protein expression in WT male mice after one-week of saline or lanreotide treatment (n=4/group). Asterisk indicates the significance of the difference between Saline and Lanreotide group, *p<0.05. **(C)** %Change in body mass relative to starting weight (n=4/group) and (**D**) gram of food eaten per gram of mouse body weight (n=4) during the one-week lanreotide treatment. Error bars are +/- standard error of the mean (SEM). *Related to Figure 1*

Figure S2: Growth Hormone Signaling, but Not Insulin, Represses H_2S Production Pathways *in vivo.* (A) Hepatic CGL and CBS protein expression in male mice injected for two weeks with saline, IGF-1, or GH (n=5/group). Asterisk indicates the significance of the difference between the experimental groups and the saline control group, *p<0.05. **(B)** Hepatic CGL mRNA expression in control or GH injected rats. Original source data from NCBI GeoProfile GDS862 / 8.2.2.10 / Cth (Ahluwalia, Clodfelter, & Waxman, 2004). Asterisk indicates the significance of the difference between Control and +GH, *p<0.05. **(C)** Liver CGL and CBS protein expression from male and female WT and IRS-1 KO mice (n=3/group). Asterisk indicates the significance of the difference between WT and KO within sex, *p<0.05. (**D**) Hepatic H_2S production capacity in WT or liver specific insulin receptor knockout; L-IRKO, mice (n=5). Asterisk indicates the significance of the difference between WT and L-IRKO, *p<0.05. (**E**) Analysis of H_2S producing enzymes CGL and CBS in the livers of WT or FGF21 Over Expressing; OE, mice (n=6). Asterisk indicates the significance of the difference between WT and FGF21 OE, *p<0.05. Error bars are +/- standard error of the mean (SEM). *Related to Figure 2*

Figure S3: Growth Hormone Signaling Represses H_2S Production Pathways *in vitro.* (A-B) Endogenous H_2S production in WT or CGL KO primary mouse dermal fibroblasts as measured via florescent probe and 2-photon florescent microscopy **(A)** or spectrophotometry **(B)** under different media conditions +/-PAG&AOAA (n=3). Asterisk indicates the significance of the difference between Complete and –Serum, and pound indicates the significance of the difference between CGL WT–Serum and CGL KO-Serum or the –Serum+PAG+AOAA groups, */#p<0.05. Scale bars indicate 100μm. (**C**) Measurement of endogenous H_2S production via florescent probe and spectrophotometry (n=3) in mouse primary hepatocytes under various growth conditions +/-Serum+/-GH. Asterisk indicates the significance of the difference between Complete and pound indicates the significance of the difference between –Serum and –Serum+GH; $^{*/\#}$p<0.05. (**D-E**) Western blot analysis of phospho- and total- Stat5 protein expression in primary hepatocytes grown w/ serum (**D**) and w/o serum (**E**) as a function of GH addition. (**F**) *IGF-I* mRNA expression in mouse primary hepatocytes as a function of +/-Serum and +/-GH in the media (n=3). Asterisk indicates the significance of the difference between Comp-GH, and pound indicates the significance of the difference between -Serum-GH and -Serum+GH; */#p<0.05. (**G-H**) Measurement of endogenous H_2S production via P3 florescent probe and spectrophotometry in mouse primary hepatocytes under various growth conditions: (**G**) +/-Serum +/-GH +/-AZD1480, (**H**) +/-FGF21. Asterisks indicates the significance of the difference between Complete, pound indicates the significance of the differences between -Serum, and dollar sign indicates the significance of the difference between +GH; */#/$p<0.05. (**I**) CGL and CBS mRNA expression in primary mouse hepatocytes grown in complete (Comp) media or in media lacking serum (-Serum), (n=3). (**J**) Western blot analysis of CGL and CBS expression in primary hepatocytes grown in Complete or –Serum media (n=2). (**K-L**) Measurement of endogenous H_2S production via florescent probe and spectrophotometry in mouse primary hepatocytes grown in media +/- Serum and +/- the autophagy inhibitor BAF (n=3) **(K)** or CQ (n=3) **(L)**. Asterisks indicates the significance of the difference between Complete and -Serum and pound sign indicates the significance of the difference between -Serum-BAF and -Serum+BAF **(K)**, or –Serum-CQ and –Serum+CQ **(L)**, */#<0.05. (**M-N**) Western blot analysis showing knockdown of ATG5 or ATG7 in hepa1-6 cells with lentiviral shRNA **(M)** or ATG5 and ATG7 knockout in MEF cells **(N)**. Error bars are +/- standard error of the mean (SEM). *Related to Figure 3*

Figure S4: Hypothyroidism Boosts and Thyroid Hormone Represses Hepatic H_2S Production and alters TSP Pathway *in vivo.* (**A**) Thyroid hormone receptor target gene mRNA expression (n=4) and (**B**) fresh homogenate H_2S production capacity (n=6) in livers from mice under various hypo-, hyper-, or eu-thyroid states. Asterisk indicates the significance of the difference from the hypothyroid (PTU+Saline) state; *p<0.05. **(C)** Liver metabolites related to sulfur amino acid metabolism in mice treated with T3 vs. vehicle control (saline) as indicated (n=5/group). Asterisk indicates the significance of the difference between vehicle and +T3; *p<0.05. Error bars are +/- standard error of the mean (SEM). *Related to Figure 4*

Figure S5: Hyperthyroidism Modifies the Hepatic Methionine Cycle and Transsulfuration Pathway *in vivo*. (A) Liver metabolites related to sulfur amino acid metabolism in mice treated with GC-1 vs. vehicle control (saline) as indicated (n=6-8). Asterisk indicates the significance of the difference between vehicle and +GC-1; *p<0.05. **(B)** Liver mRNA expression of thyroid hormone target related and sulfur amino acid metabolism related genes as a function of liver thyroid hormone receptor beta (TRb) status (n=4-5/group). Asterisks indicates the significance of the difference from WT and pound indicates the significance of the differences between Het ($TRb^{PV/+}$) and homo ($TRb^{PV/PV}$); $^{*/\#}$p<0.05. **(C,D)** Liver ATF4 protein expression from male and female Snell Dwarf and WT **(C**, n=3/group**)** and GHRKO and WT **(D**, n=3/group**)** mice. Asterisks indicate the significance of the difference between WT and experimental group within sex. *p<0.05. Error bars are +/- standard error of the mean (SEM). *Related to Figure 5*

Figure S6: Thyroid Hormone and Growth Hormone Effects on Hepa1 Cell Sulfur Amino Acid Metabolism Genes mRNA Expression. Analysis of mRNA expression in Hepa1 cells after overnight treatment with T3, GH, or T3 and GH (n=3). Asterisk indicates the significance of the difference from the Control group and pound indicates the significance of the difference from the T3 & GH group; $^{*/\#}$p<0.05. Error bars are +/- standard error of the mean (SEM). *Related to Figure 6*

Figure S7: CGL Status Alters the Effects of Experimental Hypothyroidism. (**A**) Quantitation of Western blot data on H_2S producing enzymes normalized to actin (n=3) in the livers of CGL WT and KO mice after 3-weeks of Normal or PTU diet. Asterisk indicates the significance of the difference between Normal and PTU diet groups within genotype; *p<0.05. (**B, C**) Food eaten per gram of mouse (n=4-5, **B**) and changes in body mass (n=4-5, **C**) in CGL WT and KO mice on Normal or PTU diets for 3-weeks. (**D**) Body mass of CGL WT and KO mice before and after a 3-day fast (n=3-4/group). **(E)** Liver H_2S production capacity from mice injected with AD-Null (control) or AD-CGL (CGL overexpression) 1-week prior (n=4/group). **(F)** Serum T4 (ug/dL) after a two day fast in CGL KO mice +/- NaHS supplementation (n=4/group). (**G-H**) Serum IGF-1 in mice treated with a single NaHS injection 24-hours prior (n=4/group , **G**) and in mice given drinking water supplemented with NaHS and GYY4137 over a two week period (n=4, **H**). Asterisks indicate the significance of the difference compared to baseline serum IGF-1 levels; *p<0.05. Error bars are +/- standard error of the mean (SEM). *Related to Figure 7*

REAGENT or RESOURCE	SOURCE	IDENTIFIER
**Oligonucleotides**		
IGF-1 F: TGCTTGCTCACCTTCACCA IGF-1 R: CAACACTCATCCACAATGCC	N/A	N/A
GHR F: ATTCACCAAGTGTCGTTCCC GHR R: TCCATTCCTGGGTCCATTCA	N/A	N/A
CGL F: TTGGATCGAAACACCCACAAA CGL R: AGCCGACTATTGAGGTCATCA	N/A	N/A
CBS F: GGGACAAGGATCGAGTCTGGA CBS R: AGCACTGTGTGATAATGTGGG	N/A	N/A
HPRT F:TTTCCCTGGTTAAGCAGTACAGCCC HPRT R:TGGCCTGTATCCAACACTTCGAGA	N/A	N/A
RPL13 F:TTCGGCTGAAGCCTACCAGAAAGT RPL13 R:TCTTCCGATAGTGCATCTTGGCCT	N/A	N/A
MAT1A F: GATAGCAGATCTGAGGCGCT MAT1A R: TGCACCATTATCCTGCATGT	N/A	N/A
GNMT F: AAGAGGGCTTCAGCGTGATG GNMT R: CTGGCAAGTGAGCAAAACTGT	N/A	N/A
AHCY F: CGCCAGCATGTCTGATAAAC AHCY R: CCTGGCATCTCATTCTCAGC	N/A	N/A
BHMT F: TTAGAACGCTTAAATGCCGGAG BHMT R: GATGAAGCTGACGAACTGCCT	N/A	N/A
Cyclophilin F: GGTGGAGAGCACCAAGACAGA Cyclophilin R: GCCGGAGTCGACAATGATG	N/A	N/A
CGL	N/A	Mm00461247_m1
CBS	N/A	Mm00460654_m1
MTR	N/A	Mm01340053_m1
DIO1	N/A	Mm00839358_m1
GPD2	N/A	Mm00439082_m1
THRSP	N/A	Mm01273967_m1
GSTA2	N/A	Mm00833353_mH
FBXO21	N/A	Mm00523921_m1

TSH alpha	N/A	Mm01209400_m1
TSH beta	N/A	Mm00437190_m1
PGC-1a	N/A	Mm01208835_m1
FGF21	N/A	Mm00840165_g1
MAT1A Promoter F: CTCCTCCACTGTCCTTGCTTG<br>MAT1A Promoter R: GGCAGATCTTTGGCAGAATCC	N/A	N/A
MAT1A intron 4 F: CACGTGCATGGGTAGAGGGAC<br>MAT1a intron 4 R: CTTTTCCACCTCCCCGAGGTC	N/A	N/A
GNMT exon 5 F: GGCTCAGGGAATGACGCAACC<br>GNMT exon 5 R: CTCTCCTGCCAAACCACGTCA	N/A	N/A
CBS intron 1 F: CCTTGTGCGGGACCCAGTTGG<br>CBS intron 1 R: GCGAATGTGGCCCAGGGTATC	N/A	N/A
CBS intron 2 F: GCACACCTGACCCTGTATCC<br>CBS intron 2 R: CTGCTGTCCTTGGTTGGACT	N/A	N/A
CGL promoter F: GGGCCTTAAGGCCTGATCTTG<br>CGL promoter R: CAGCACTGAGGTGCAGCACTC	N/A	N/A
BHMT promoter F: CGCATCCTCATGCAATGATC<br>BHMT promoter R: CTGAGGTCGCTGCTAGCTCGTC	N/A	N/A

Supplemental Table S1: List of DNA Oligos (Related to STAR Methods Key Resources Table)
